# Visible-Light-Controlled Formation of G‑Quadruplexes

**DOI:** 10.1021/acs.orglett.6c02618

**Published:** 2026-07-09

**Authors:** Jorge S. Valera, Jorge Rodríguez-Durán, Jorge J. Cabrera-Trujillo, David González-Rodríguez

**Affiliations:** † Nanostructured Molecular Systems and Materials Group, Organic Chemistry Department, Science Faculty, 16722 Universidad Autónoma de Madrid, 28049 Madrid, Spain; ‡ Departamento de Química Orgánica, Facultad de Ciencias, Universidad de La Laguna (ULL), 38206 La Laguna, Spain; § Instituto Universitario de Bio-Orgánica Antonio González (IUBO-AG), Universidad de La Laguna (ULL), 38206 La Laguna, Spain; ∥ Institute for Advanced Research in Chemical Sciences (IAdChem), Universidad Autónoma de Madrid, 28049 Madrid, Spain

## Abstract

Guanine (G)-quadruplexes are robust supramolecular assemblies formed by the π-stacking of hydrogen-bonded G-quartets around a metal cation. We report visible light control of G-quadruplex formation through direct functionalization of guanosine with *ortho*-substituted azobenzene (osAZO) and arylazopyrazole (AAP) photoswitches. The *E* isomers form *D*_4_-symmetric G-octamers, while the *Z* isomers induce disassembly. Spectroscopic and theoretical analyses unravel distinct switching efficiencies, with the AAP derivative enabling near-quantitative reversible control. This is the first visible-light-controlled G-quadruplex assembly via direct guanine modification.

## Graphical abstract

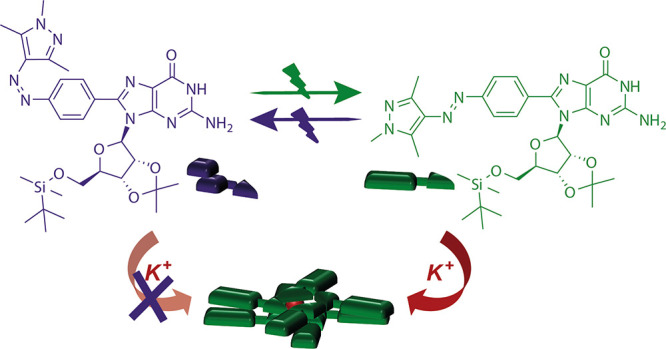

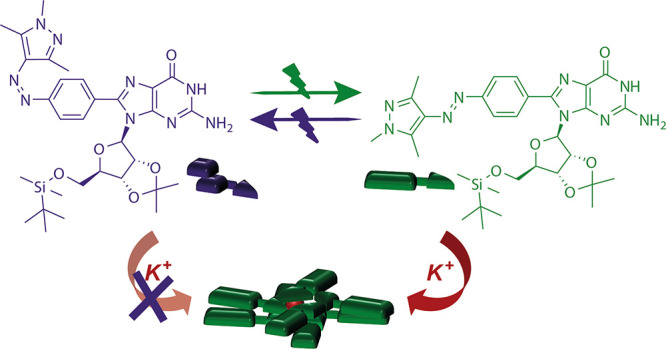

Guanine (G) and related nucleobases constitute powerful building blocks for the construction of well-defined supramolecular architectures through programmed self-association.^1–5^ Among these, G-quadruplexes are unique assemblies formed by the π-stacking of H-bonded G-quartets (G_4_) around a coordinated metal ion, like Na^+^ or K^+^.^6–8^ In organic solvents, G derivatives typically organize into discrete octameric, dodecameric, or hexadecameric species (G_8_, G_12_, or G_16_), whose strong cooperativity endows them with exceptional robustness and kinetic stability.^8–10^ Furthermore, chemical modification at the N^2^ or C^8^ positions provides access to G-quadruplexes with tunable structural and functional properties.^11–19^

Despite these advantages, the high stability and kinetic persistence of G-quadruplexes hamper their implementation in dynamic materials, where reversible and externally controlled assembly would be advantageous.^20–22^ Light is particularly attractive as a stimulus owing to its non-invasive character and high spatiotemporal precision.^23–27^ Although light has been employed to regulate G-quadruplex formation through a variety of strategies,^28–30^ only one example has achieved such control through direct covalent modification of the guanine building block.^31^ Notably, this system relies on UV irradiation, thereby limiting its applicability in material and biological contexts.

Visible-light-responsive photoswitches, like *o*-substituted azobenzenes (osAZO)^32^ and arylazopyrazoles (AAPs),^33^ offer an appealing alternative, since they combine long-lived metastable states with efficient bidirectional switching using visible light. These characteristics make them highly promising candidates for expanding the toolbox of responsive G-quadruplex systems and enabling their integration into advanced functional materials.^21^

Herein, we report the visible-light-controlled formation of G-quadruplexes through direct N^2^ (**G1**) and C^8^ functionalization (**G2** and **G3**) of guanosine with osAZO (**G1** and **G2**) and AAP (**G3**) photoswitches (Figure 1). Whereas **G1** does not form G-quadruplexes, the *E* isomer of **G2** and **G3** assemble into discrete *D*_4_-symmetric G-octamers that undergo light-triggered disassembly upon *E* → *Z* photoisomerization. Notably, the AAP derivative **G3** exhibits nearly quantitative visible-light-controlled G-octamer formation. To the best of our knowledge, this study constitutes the first example of visible-light-regulated G-quadruplex formation achieved via direct covalent modification of the guanine scaffold and provides a foundation for the development of photoresponsive supramolecular materials.

**Figure 1:**
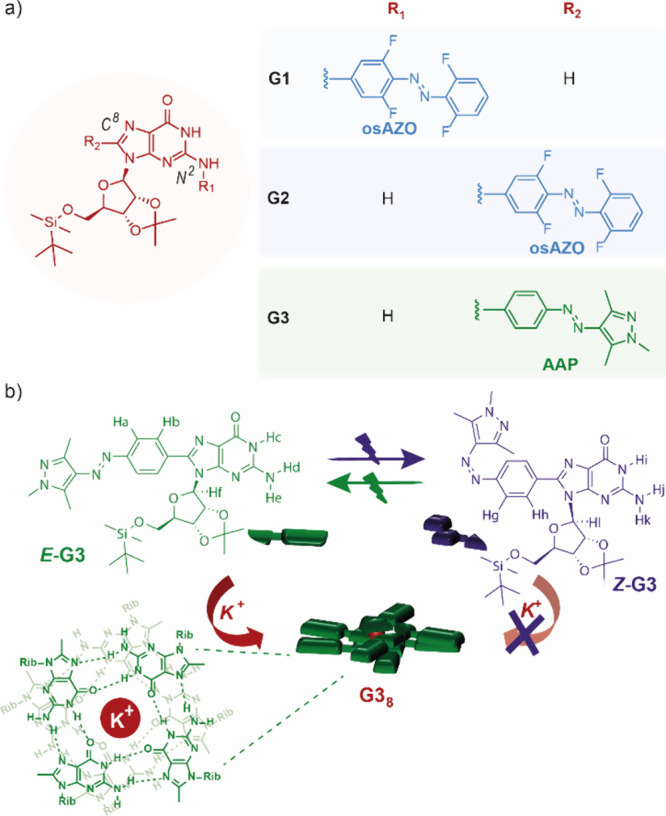
(a) Chemical structures of the photoswitchable G derivatives **G1**, **G2**, and **G3**. (b) Photoisomerization and visible-light-controlled formation of G-quadruplexes by **G3**, with a schematic depiction of the H-bonding pattern in the formation of G-quadruplexes.

The photoactive guanosine derivatives **G1**, **G2**, and **G3** were synthesized through C–N or C–C cross-coupling reactions from adequately substituted azo-switches and G derivatives, adapting previously reported protocols.^15,34^ Full synthetic details and characterization data can be found in the Supporting Information accompanying this paper.

The photoswitching properties of **G1** were first investigated in the molecularly dissolved state by ^1^H and ^19^F NMR spectroscopies using DMSO-*d*_6_ as the solvent (Figures S1 and S2).^32,35^ Initially, a mixture of both isomers is observed in a 79:21 *E*/*Z* proportion. Whereas irradiation at 525 nm afforded a PSS with an *E*/*Z* composition of 23:77, irradiation with 427 nm provides a PSS with a 74:26 *E*/*Z* ratio. However, none of these isomer compositions resulted in the formation of G-quadruplexes upon the addition of potassium salts, likely due to steric congestion at the Watson–Crick edge. These results, together with the modest photoswitching efficiencies, prompted us to investigate C^8^-functionalization instead.

**G2** presents similar photoswitching behavior, reaching PSS compositions of 20:80 and 52:48 *E*/*Z* ratios upon irradiation at 525 and 427 nm, respectively (Figures S3 and S4). The lower efficiency of the *Z* → *E* photoisomerization is consistent with the stronger absorption of the *E* isomer throughout the visible region (Figure S5).

In contrast, **G3** shows an improved photoswitching performance (Figure 2a).^33,36,37^ Irradiation at 390 nm generated a PSS with an *E*/*Z* ratio of 14:86, whereas irradiation at 525 nm restored almost quantitatively the *E* isomer (93:7). UV–vis spectroscopy unraveled distinct absorption profiles for the isomers, with maxima at 450 and 370 nm for the *Z* and *E* forms, respectively (Figure 2b). Thermal isomerization studies at 293 K disclose long-lived metastable states for both derivatives, with half-lives of 149 days for **G2** and 2.1 days for **G3**, in agreement with related systems^35,38^ (Figures S6–S9 and Table S1).

**Figure 2:**
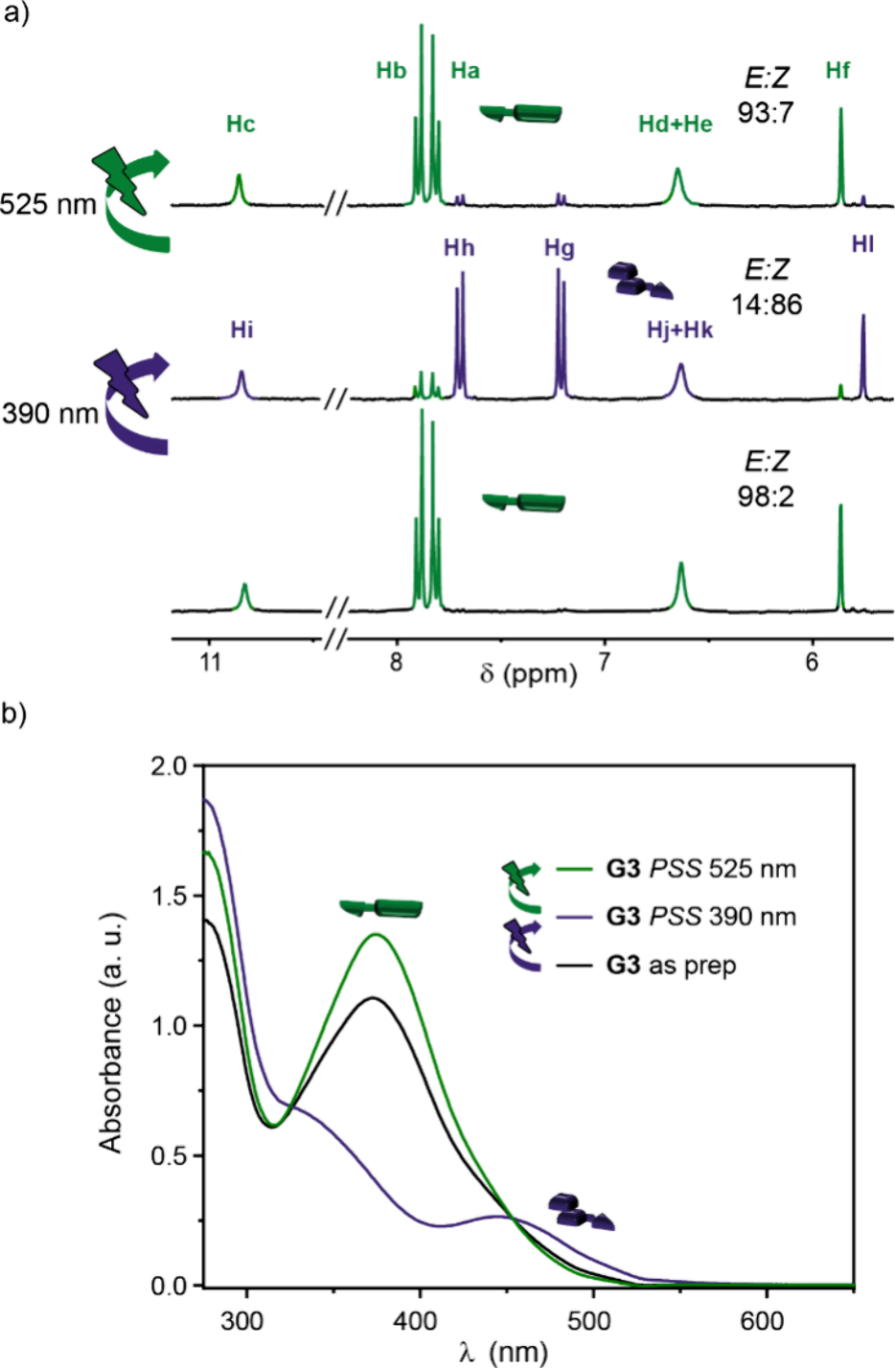
(a) Partial ^1^H NMR spectra of **G3** in DMSO-*d*_6_ as prepared and after irradiation with 390 and 525 nm. [**G3**] = 10 mM. For proton labeling, see Figure 1. (b) UV–vis spectra of **G3** in DMSO as prepared and after irradiation with 390 and 525 nm. [**G3**] = 50 μM, and *l* = 1 cm.

We evaluated the formation of G-quadruplexes in THF-*d*_8_ in the presence of potassium salts (KPF_6_ and KBPh_4_). For **G2**, the initial solution without salt shows a 39:61 *E*/*Z* composition, as revealed by ^1^H and ^19^F NMR spectroscopies (Figures S10 and S11). The addition of KPF_6_ leads to the emergence of a new set of ^19^F and ^1^H NMR signals, with two new peaks in ^19^F NMR and the shift of the G-amide N–H proton from a broad resonance centered at δ = 10.7 ppm to a sharp signal at δ = 12.5 ppm, which are attributes for the formation of a *D*_4_-symmetric octamer (*D*_4_-**G2**_8_).^6,7,10,15,16^ However, this quadruplex is not formed quantitatively, presumably due to low stability. Interestingly, the quadruplex seems to be constituted solely by the *E* isomer (with an estimated distribution *E*/*Z*/G_8_ of 25:68:7). This is supported by the disappearance of the H-bonded N–H signal in the complex at δ = 12.5 ppm after irradiation with 525 nm, which affords a PSS with an 11:89 *E*/*Z* proportion. Subsequent irradiation with 427 nm provides a PSS richer in the *E* state and thus in the *D*_4_-**G2**_8_ complex (*E*/*Z*/G_8_ composition of 31:44:25). However, the low *E* isomer content in this PSS precludes the quantitative formation of **G2**_8_ in all the conditions explored.

This promising but not ideal situation improves drastically for **G3** (Figure 3a and Figures S12 and S13). The pristine solution of this molecule in THF-*d*_8_ already showcases a major *E* isomer composition (88:12 *E*/*Z*). The addition of 0.25 equiv of KBPh_4_ or KPF_6_ results in the almost quantitative transformation to the *D*_4_-symmetric *E*-**G3**_8_ complex with the shift of G-amide N–H from δ = 10.9 to 12.7 ppm (Figure S12). Subsequent irradiation with 390 nm leads to a PSS with a ≈10:90 ratio, where the octameric N–H signal at δ = 12.7 ppm vanishes. Notably, the spectrum of the *Z* isomer does not change significantly upon the addition of K salts (Figure S13). Further irradiation with 525 nm restores the *E* isomer almost quantitatively and hence the *D*_4_-symmetric *E*-**G3**_8_ quadruplex, with the recovery of the signal at δ = 12.7 ppm. Exceptionally, this species is remarkably stable, remaining the predominant species at concentrations as low as [**G3**] = 5.0 × 10^–4^ M (Figure S14).

**Figure 3:**
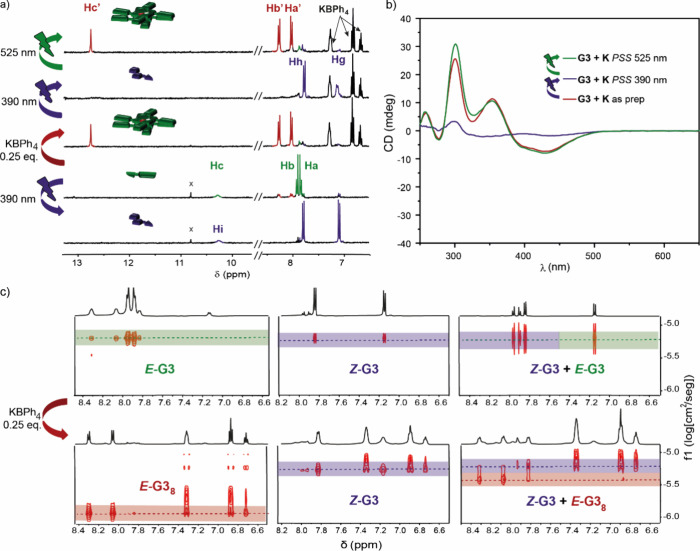
(a) Partial ^1^H NMR spectra of **G3** in THF-*d*_8_ after irradiation with 390 nm, as-prepared sample, after the addition of 0.25 equiv in KBPh_4_, and after irradiation with 390 and 525 nm to the solution with KBPh_4_. Protons marked in red, green, and purple denote the formation of *E*-**G3**_8_, *E*-**G3**, and *Z*-**G3**, respectively. [**G3**] = 2.5 mM. For proton labeling, see Figure 1. (b) CD spectra of **G3** in THF in the presence of 0.25 equiv of KPF_6_ as prepared and after irradiation with 525 and 390 nm. [**G3**] = 500 μM, and *l* = 1 mm. (c) Amplification of the DOSY spectra of *E*-**G3**, *Z*-**G3**, and an *E*/*Z*-**G3** mixture (from left to right, upper panels) and *E*-**G3**, *Z*-**G3**, and an *E*/*Z*-**G3** mixture with KBPh_4_ (from left to right, lower panels) in THF-*d*_8_ at 298 K. [**G3**]_tot_ = 2.5 mM.

We further explored the features of the discrete assemblies by ^1^H DOSY NMR (Figure 3c, Figures S15–S20, and Table S2). The DOSY NMR spectrum of *E*-**G3** in the monomeric state affords an average diffusion coefficient of *D*_Av_ = (5.8 ± 0.4) × 10^–10^ m^2^ s^–1^. The addition of KBPh_4_ leads to the emergence of a species with *D*_Av_ = (3.7 ± 0.1) × 10^–10^ m^2^ s^–1^, which is consistent with the formation of the *D*_4_-symmetric *E*-**G3**_8_ quadruplex. In the case of *Z*-**G3**, we did not observe significant changes in the diffusion coefficients after the addition of the K salt. We also performed a control experiment with a 1:1 mixture of *E*-**G3** and *Z*-**G3** in the monomeric state and in the presence of KBPh_4_, revealing the formation of the *E*-**G3**_8_ complex in the presence of K, whereas *Z*-**G3** remained in the monomeric state, which further supports the formation of the octameric quadruplex solely by the *E* isomer. NOESY NMR experiments of *E*-**G3** with KBPh_4_ enable us to identify mostly contacts that are assignable to intramolecular cross-peaks, with an additional contact between the G-amide proton and one of the protons of the *para*-substituted system in the AAP, which correlates with the *D*_4_-G_8_ structure (Figure S21).

We also performed studies of the **G3** self-assembly by UV–vis and CD spectroscopies (Figure 3b and Figures S22 and S23). In UV–vis, the pristine solution of **G3** exhibits an absorption band centered at 370 nm, mostly attributable to the *E* isomer. Irradiation with 390 nm yields a spectrum mainly corresponding to the *Z* isomer, with a maximum centered at 450 nm, which after 525 nm irradiation affords again mainly the *E* isomer absorption pattern. The addition of the salt maintains this photoswitching behavior with a slight hypsochromic shift from 370 to 365 nm in the *E* isomer, which could be indicative of a weak H-type coupling (Figure S22). In CD spectroscopy, the changes were more marked. Without salt, the transition between the *E* and *Z* isomers leads to an inversion of the weak chiroptical features (Figure S23). However, in the presence of KPF_6_, an intense new chiroptical signature arises in the *E* isomer form, whereas the spectrum mainly attributable to the *Z* isomer retains the features observed in the absence of salt (Figure 3b). The observed negative bisignate Cotton effect has been ascribed to the formation of head-to-head (H-to-H) heteropolar stacking.^39^ Thus, spectroscopic measurements are consistent with the formation of *D*_4_-symmetric *E*-G_8_ supramolecular structures.

We performed computational studies on both the monomeric and octameric species of **G3**. *E*-**G3** and *Z*-**G3** monomers were first optimized by DFT calculations (Figure S24; see the Supporting Information for complete computational details). *E*-**G3** adopts an extended and comparatively planar arrangement that is more compatible with the formation of stacked G-quartets, whereas *Z*-**G3** displays a twisted geometry that is less favorable for supramolecular packing. The reliability of these optimized geometries was further supported by TD-DFT simulations of the UV–vis absorption spectra, which for both cases reproduced the main features of the experimental profiles (Figure S25).

We next optimized the G_8_ assemblies using the semi-empirical GFN2-xTB method, which is suitable for large molecular systems (ca. 1000 atoms).^40^ We evaluated two arrangements for *E*-**G3**_8_, namely, H-to-H and head-to-tail (H-to-T) arrangements, surrounding a centrally coordinated K^+^ cation (Figure 4 and Figure S26).^39,41^ The H-to-H arrangement was more stable than the H-to-T structure by 25.0 kcal/mol based on DFT single-point energy calculations, and this, together with the good correlation between the calculated and experimental ECD spectra at lower wavelengths (Figure S27), supports a *D*_4_-symmetric H-to-H arrangement as the most plausible structure.

**Figure 4:**
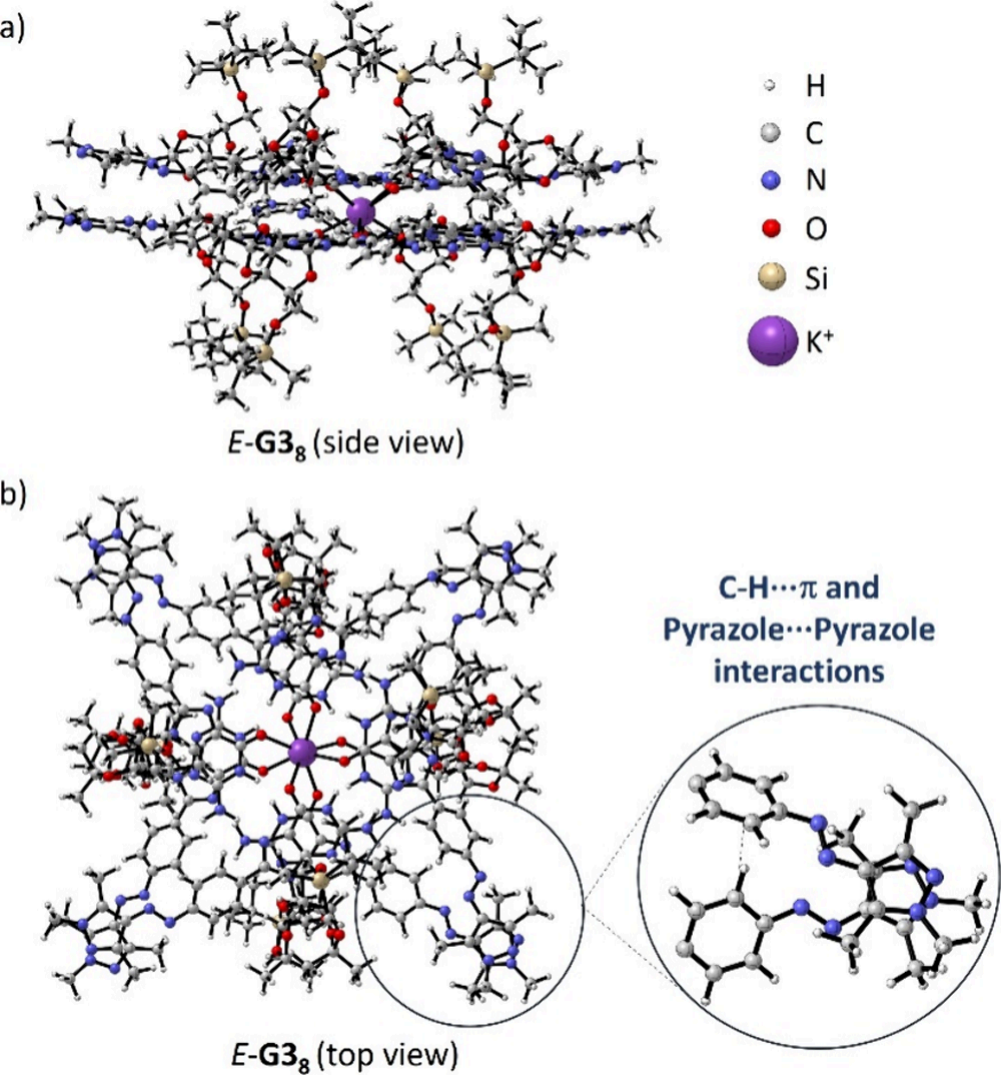
(a) Side and (b) top views of the H-to-H arrangement of *E*-**G3**_8_ optimized at the ALPB­(THF)-GFN2-xTB-level of theory. In panel b, the stabilizing interactions in the arylazopyrazole moiety are amplified and highlighted. The assemblies consist of two stacked G-quartets surrounding a coordinated K^+^ cation.

Although a *Z*-**G3**_8_ geometry could be located computationally, indicating that such an arrangement is geometrically feasible (Figure S28), DFT single-point energy calculations revealed that it is markedly less stable than *E*-**G3**_8_ by 95.1 kcal/mol. This large energy difference is consistent with the experimental absence of *Z*-**G3**_8_ and suggests that this assembly is unlikely to exist in the conditions studied (Figure S29). We also noticed the presence of C–H···π stabilizing interactions between the aryl rings as well as further π–π stacking of the photoswitch pyrazole rings in *E*-**G3**_8_ (see the highlight in Figure 4b). These interactions are not present for *Z*-**G3**_8_ and might strongly contribute to the difference in stability observed for the two quadruplexes. Moreover, we noted that the photoswitch and ribose groups in *Z*-**G3** are forced to adopt very specific relative conformations to avoid steric hindrance. This would diminish the degrees of freedom of this photoisomer upon self-assembly, which must result in a strong entropic penalty. In short, computational calculations further correlate with the observed experimental data and support a rationale in which light-induced changes in the geometry of the AAP unit modulate the relative stability of the G_8_ architecture.

Thus, visible-light-responsive guanosine derivatives carrying osAZO and AAP photoswitches permit reversible control over G-octamer formation. Whereas **G2** provides moderate modulation, **G3** exhibits a highly efficient light-controlled assembly–disassembly process using visible light. Spectroscopic and theoretical analyses support the formation of the H-to-H, *D*_4_-G_8_ in the *E* isomer and its disruption upon photoisomerization to the *Z* isomer. These findings establish visible light photoswitching as an effective strategy to regulate guanosine self-assembly and create adaptive supramolecular materials.

## Supplementary Material



## Data Availability

The data underlying this study are available in the published article and its Supporting Information.

## Notes

Experimental procedures, characterization data, NMR spectra, additional figures, and computational calculations (PDF)
