# Supplementary material for: Visible-Light-Controlled Formation of G‑Quadruplexes
Source: Org Lett. 2026 Jul 9;28(29):9505–10. doi: 10.1021/acs.orglett.6c02618 (PMC13411067; doi:10.1021/acs.orglett.6c02618)
Supplement: Supplementary file 1 [file ol6c02618_si_001.pdf]

SUPPORTING INFORMATION

Visible light-controlled formation of G-quadruplexes

Jorge S. Valera*, Jorge Rodríguez Durán, Jorge J. Cabrera-Trujillo and David González Rodríguez*

[1] Nanostructured Molecular Systems and Materials Group, Organic Chemistry Department, Science Faculty, Universidad Autónoma de Madrid 28049 Madrid, Spain.
E-mail: jorge.valera@uam.es & david.gonzalez.rodriguez@uam.es
[2] Departamento de Química Orgánica, Facultad de Ciencias, Universidad de La Laguna (ULL), 38206 La Laguna, Spain.
[3] Instituto Universitario de Bio-Orgánica Antonio González (IUBO-AG), Universidad de La Laguna (ULL), 38206 La Laguna, Spain.
[4] Institute for Advanced Research in Chemical Sciences (IAdChem), Universidad Autónoma de Madrid, 28049 Madrid, Spain.

Corresponding authors: jorge.valera@uam.es and david.gonzalez.rodriguez@uam.es

Materials and Methods

Synthetic Procedures and Characterization Data

Supporting Figures

Computational Details

Materials and methods

All reagents and solvents were obtained from commercial suppliers and used without further purification. Solvents for spectroscopic studies were of spectroscopic grade and used as received. Column chromatography was carried out on silica gel Merck-60 (230-400 mesh, 60 Å), and TLC on aluminum sheets precoated with silica gel 60 F254 (Merck).

NMR experiments. ^{1}H-NMR, ^{13}C-NMR and ^{19}F-NMR were recorded with a BRUKER AVANCE-II 300 MHz or a BRUKER DRX 500 MHz instrument. The temperature was actively controlled at 298 K. Chemical shifts are measured in ppm using the signals of the deuterated solvent as the internal standard [$CDCl_3$ calibrated at 7.27 ppm (^{1}H) and 75.0 ppm (^{13}C), DMSO-d_6 calibrated at 2.5 ppm (^{1}H) and 39.5 ppm (^{13}C), THF-d_8 calibrated at 3.58 ppm (^{1}H) and 67.5 ppm (^{13}C)]. Coupling constants (*J*) are denoted in Hz and chemical shifts (δ) in ppm. Multiplicities are denoted as: s = singlet, d = doublet, t = triplet, m = multiplet, br = broad. For the ^{1}H DOSY NMR experiments, a series of diffusion ordered spectra were collected from the samples using the LEDbp pulse sequence. The pulse fields were incremented in 16 steps from 5 % to 95 % of the maximum gradient strength in a linear ramp. Structural assignments were made with additional information from gCOSY, gHSQC, and gHMBC experiments.

Mass Spectrometry (MS) and High Resolution-Mass Spectrometry (HRMS) MALDI-TOF spectra were obtained from a BRUKER ULTRAFLEX III instrument equipped with a nitrogen laser operating at 337 nm.

UV/Vis spectroscopy: Measurements in solution were conducted using a JASCO V-660 equipment. The temperature was controlled using a JASCO Peltier thermostatted cell holder with a range of 263–383 K, adjustable temperature slope, and accuracy of ± 0.1 K.

CD spectra were recorded with JASCO J-815 equipment (measurement information: data pitch = 1 nm; sensitivity = standard; D.I.T. = 2 sec; slit width = 1000 mm; data interval = 1 nm; scanning speed = 200 nm/min). The temperature was controlled using a JASCO Peltier thermostatted cell holder with a range of 263–383 K, adjustable temperature slope, and accuracy of ± 0.1 K.

Irradiation experiments in NMR and UV Vis / CD spectroscopies were carried out by direct irradiation of the NMR tube, in a 1 mm cuvette or in a 1 cm cuvette of reduced volume. For purple, blue and green irradiations we used a Kessil PR160-purple LED lamp (30 W High Luminous DEX 2100 LED, λ_{max} = 390 nm) a Kessil PR160-blue LED lamp (30 W, High Luminous DEX 2100 LED, λ_{max} = 427 nm) and a Kessil PR160-green LED lamp (30 W High Luminous DEX 2100 LED, λ_{max} = 525 nm). The lamps were placed at 1 cm distance of the sample.

Kinetic Studies of Thermal *Z* → *E* Isomerization. To perform the kinetic studies for the thermal *Z*→*E* isomerization of **G2** and **G3**, time evolution of UV-Vis absorption spectra of **G2** and **G3** in DMSO at 5.10^{-5} M were recorded at four different temperatures, in a procedure of reported by J. Moreno *et al.*[1] For each spectrum, the corresponding amount of *Z*-form was derived from the value using the following equations: $[E(t)] = A(t) - A(Z) / A(E) - A(Z)$ and $[Z(t)] = A(E) - A(t) / A(E) - A(Z)$ and where $[E(t)]$, $[Z(t)]$) and A(t) are the estimated concentration of E isomer, estimated concentration of Z isomer, and the absorbance of the mixture at time t, respectively; A(E) and A(Z) are the absorbances of pure E and Z form, respectively. Plotting the percentage of Z isomer versus time gives the rate constants k by using the first order kinetic equation $[Z] = [Z]_0 e^{-kt}$ (ExpDec1 in Origin) and the corresponding thermal half-lives with $t_{1/2} = \ln 2/k$ of the absorbance at λ_{max}.

0. Synthetic Procedures and Characterization Data

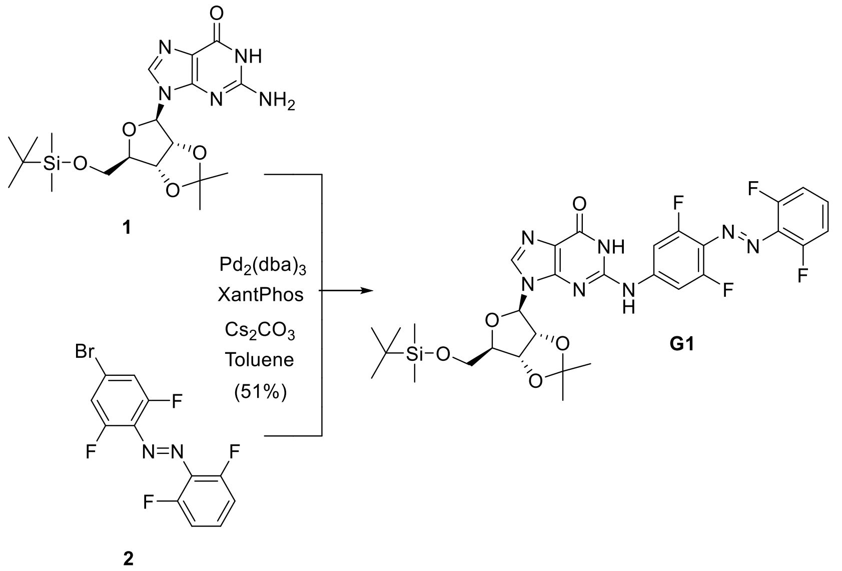

Scheme S0A. Synthetic route to **G1**. **1**[2] and **2**[1] were obtained through previously described methods and matched the spectroscopic features reported therein.

Synthesis of G1

1 (200 mg, 0.457 mmol, 1 eq.), **2** (304 mg, 0.914 mmol, 2 eq.), $Pd_2(dba)_3$ (84 mg, 0.0914 mmol, 0.2 eq.), Cs_2CO_3 (298 mg, 0.914 mmol, 2 eq.), and XantPhos (53 mg, 0.0914 mmol, 0.2 eq.) were dissolved in dry toluene (15 mL) under Ar atmosphere and the reaction was heated at 70 ºC with an oil bath overnight. Afterwards, the resulting mixture was cooled down and passed through a plug of celite. After elimination of the solvent, the resulting crude was purified though silica gel column chromatography (gradient from $CHCl_3$ to $CHCl_3$ 10:0.3 MeOH) affording **G1** (as a mixture of *E* and *Z* isomers which quickly evolves to the *E* isomer, 160 mg, 51% yield) as an orange solid.

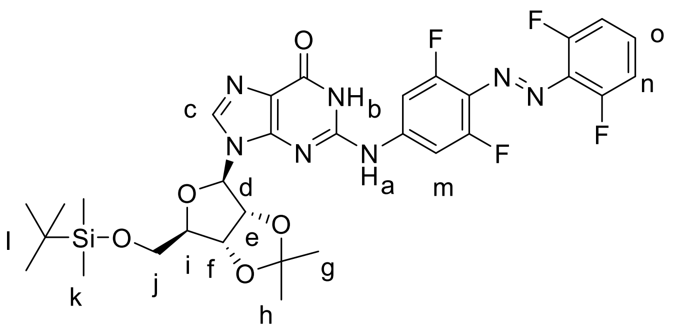

(*E*)-G1. **^{1}H-NMR** (500 MHz, DMSO-d_6): 11.16 (br, 1H_b), 9.75 (br, 1H_a), 8.08 (s, 1H_c), 7.65 (d, 2H_m, J = 13.1 Hz), 7.57 (m, 1H_n), 7.32 (t, 2H_n, J = 9.3 Hz), 6.08 (s, 1H_d), 5.36 (d, 1H_e, J = 6.0 Hz), 4.86 (d, 1H_f, J = 6.2 Hz), 4.35 (br, 1H_i), 3.75-3.65 (m,2H_j), 1.55 (s, 3$H_{h\ or\ g}$), 1.35 (s, 3$H_{g\ or\ h}$), 0.74 (s, 9H_l), -0.08 (d, 2H_k, J = 12.9 Hz). **^{19}F-NMR** (471 MHz, DMSO-d_6): δ = -118.37 (s, 2F), -122.68 (s, 2F). **^{13}C{^{1}H}-NMR** (125 MHz, DMSO-d_6): δ = 157.2, 155.6, 155.1, 153.5, 132.0-131.3, 113.2-113.0, 102.7, 102.5, 91.3, 86.6, 84.1, 81.4, 63.1, 26.9, 25.6, 25.0, 17.9, -5.7. **MS** (ESI): Calculated for $C_{31}H_{36}F_4N_7O_5Si$ $[M+H]^+$: 690.2483 Found $[M+H]^+$: 690.2479.

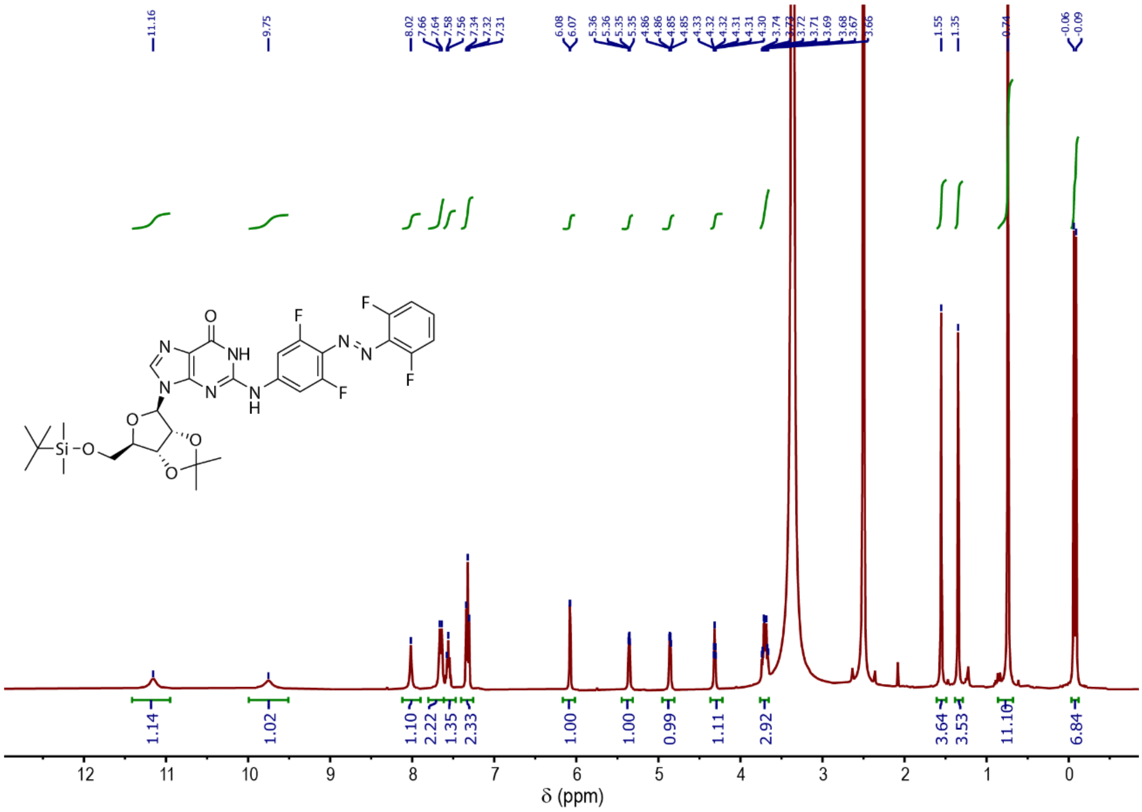

Figure S0A. ^{1}H NMR spectrum of **G1** (500 MHz, DMSO-d$_6$).

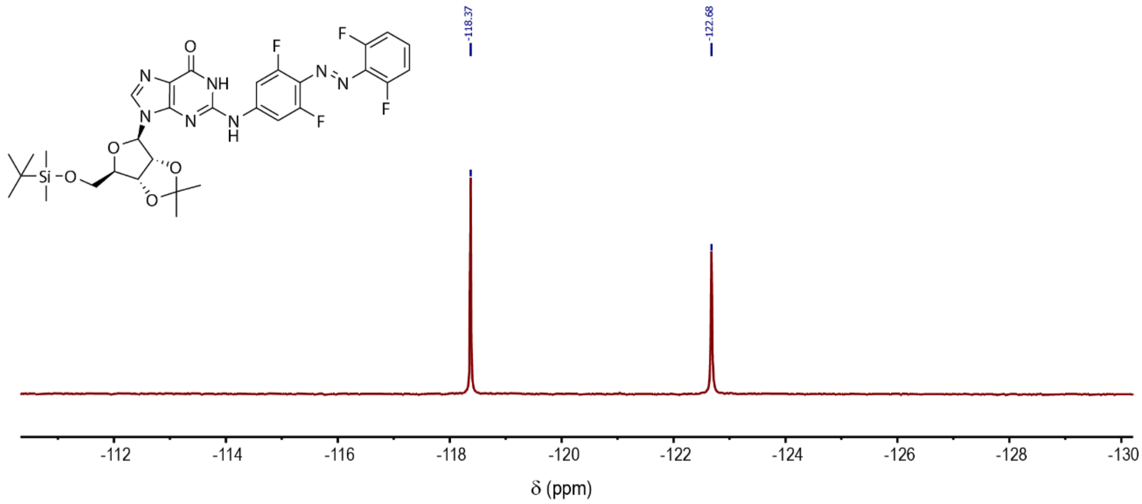

Figure S0B. ^{19}F NMR spectrum of **G1** (471 MHz, DMSO-d$_6$).

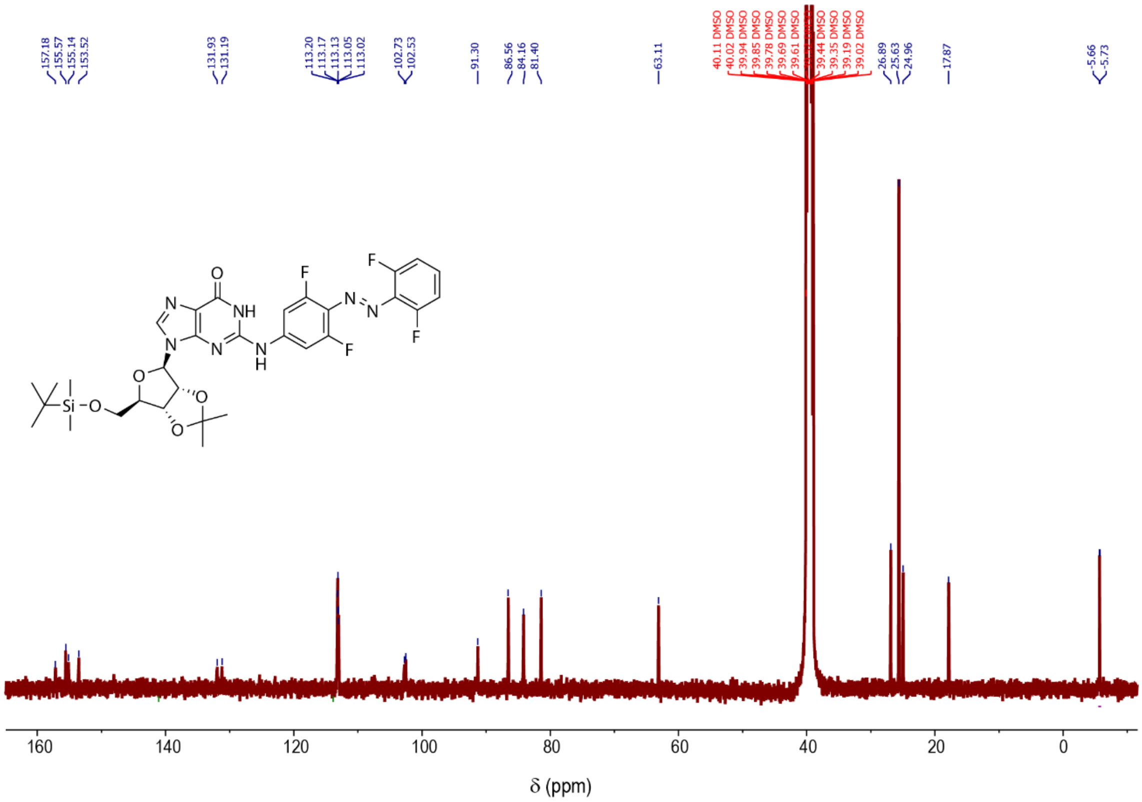

Figure S0C. ^{13}C NMR spectrum of **G1** (125 MHz, DMSO-d$_6$).

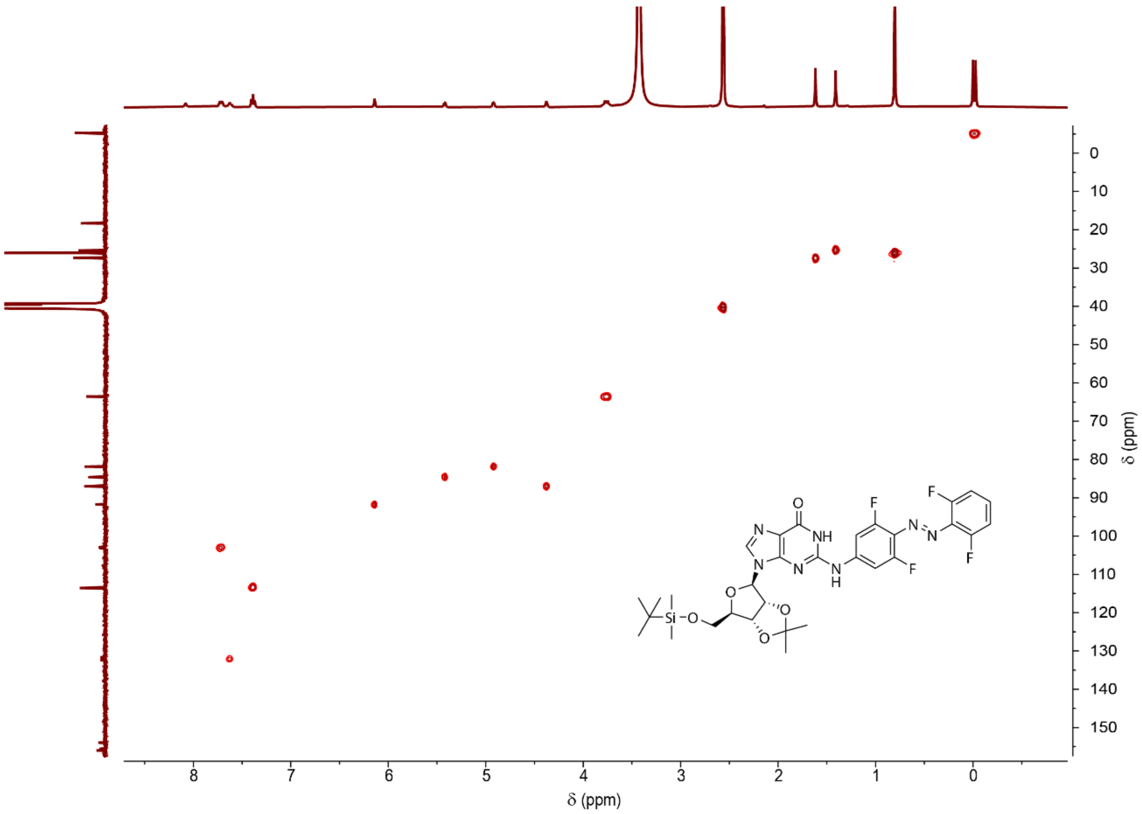

Figure S0D. ^{1}H-^{13}C HSQC spectrum of **G1** (500 MHz, DMSO-d$_6$)

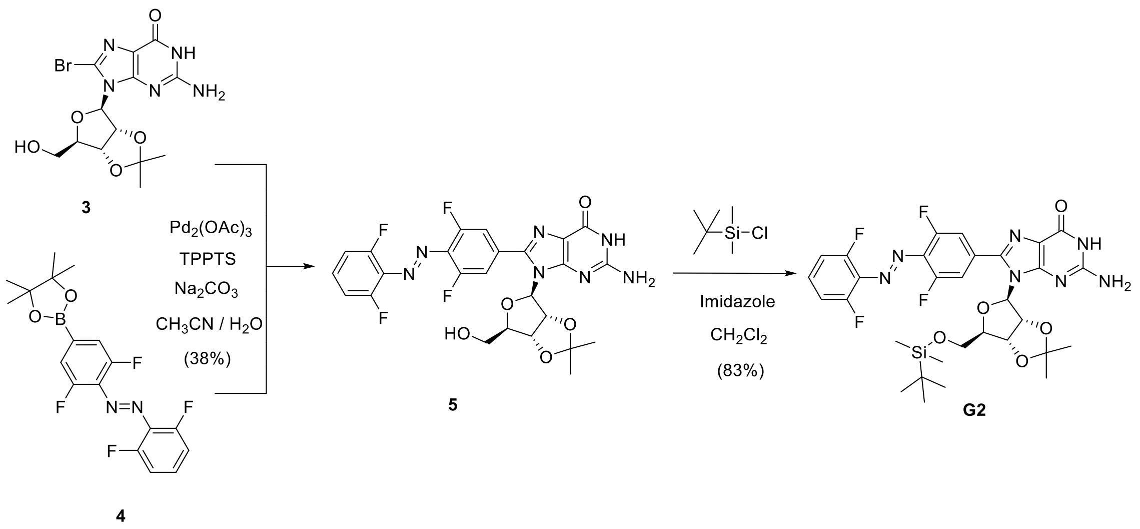

Scheme S0B. Synthetic route to **G2** **3**[2] and **4**[3] were obtained through previously described methods and matched the spectroscopic features reported therein.

Synthesis of G derivative 5

5 was obtained by partially reproducing previously described synthetic protocols.[4] Bromo guanosine derivative **3** (210 mg, 0.522 mmol, 1 eq.), boronate azoderivatives **4** (278 mg, 0.730 mmol, 1.3 eq.), $Pd_2(OAc)_3$ (22.5 mg, 0.052 mmol, 0.2 eq.) sodium triphenylphosphine trisulfonate (89 mg, 0.156 mmol, 0.3 eq.) and sodium carbonate (105 mg, 0.992 mmol, 1.9 eq.) were dissolved in a dioxane/water 1:2 mixture (9 mL x 0.15 g of **3**) and the mixture was degassed during 15 min with Ar. Afterwards, the reaction is stirred at 80 ºC with an oil bath overnight under Ar atmosphere. After that time, water is added and the organic phase is extracted with $CHCl_3$. After washing with brine, the organic phase is dried with $MgSO_4$, filtered, and the solvent is removed in the rotavapor. The resulting crude is purified by silica gel column chromatography (using a gradient from pure $CHCl_3$ to $CHCl_3$/MeOH 10:0.4 mixture as eluent), providing **5** (as a mixture of *E* and *Z* isomers, 100 mg, yield 33 %) as an orange solid.

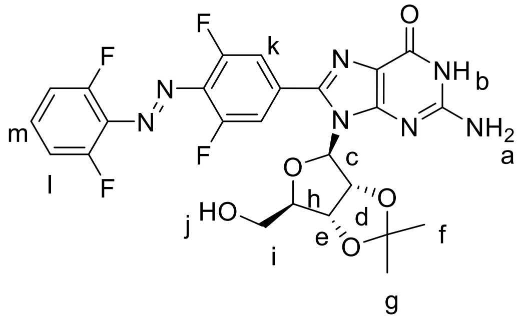

(***E***)**-5**. [1]**H-NMR** 300 MHz, DMSO-d$_6$): 10.74 (br, 1H$_b$), 7.65 (d, 2H$_k$, J = 9.9 Hz), 7.46-7.42 (m, H$_m$), 7.39 (dd, 2H$_l$, J = 9.1 Hz, J=9.1 Hz), 6.71 (br, 2H$_a$), 5.94 (d, 1H$_c$, J = 1.9 Hz), 5.47 (m, 1H$_d$), 5.21 (dd, 1H$_e$, J = 6.3 Hz, J = 3.2 Hz), 4.92 (q, 1H$_j$, J = 6.0 Hz, J = 6.0 Hz, J = 5.9 Hz), 4.16 (m, 1H$_h$), 3.54 (m, 2H$_i$), 1.48 (s, 3H$_{f\ or\ g}$), 1.31 (s, 3H$_{g\ or\ f}$). [19]**F-NMR** (282 MHz, CDCl$_3$): δ = -120.01 (s, 2F), -121.46 (s, 2F). [13]**C{**[1]**H}-NMR** (125 MHz, DMSO-d$_6$): δ = 156.6, 155.8 (br), 153.7 (br), 152.0, 151.8 (br), 149.8 (br), 143.4, 134.0-132.8, 117.3, 113.4, 113.3-112.6, 89.4, 88.7, 82.8, 81.8, 61.8, 26.9, 25.2. HR**MS** (APCI$^+$): Calculated for $C_{25}H_{22}F_4N_7O_5$ [M+H]$^+$: 576.1619. Found [M+H]$^+$: 576.1619.

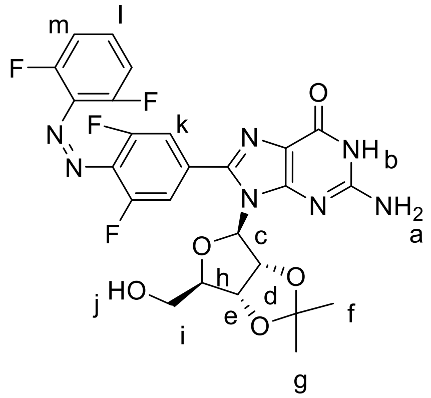

(***Z***)**-5**. [1]**H-NMR** 300 MHz, DMSO-d$_6$): 10.74 (br, 1H$_b$), 7.49 (d, 2H$_k$, J = 9.0 Hz), 7.46-7.36 (m, H$_m$), 7.22 (dd, 2H$_l$, J = 8.9 Hz, J=8.9 Hz), 6.65 (br, 2H$_a$), 5.76 (d, 1H$_c$, J = 2.1 Hz), 5.38 (dd, 1H$_d$, J = 6.3 Hz, J = 2.1 Hz), 5.06 (dd, 1H$_e$, J = 6.2 Hz, J = 3.2 Hz), 4.92 (q, 1H$_j$, J = 6.0 Hz, J = 6.0 Hz, J = 5.9 Hz), 4.10 (m, 1H$_h$), 3.50 (m, 2H$_i$), 1.44 (s, 3H$_{f\ or\ g}$), 1.28 (s, 3H$_{g\ or\ f}$). [19]**F-NMR** (282 MHz, DMSO-d$_6$): δ = -119.33 (t, 2F, J = 5.0 Hz, J = 5.0 Hz), -120.88 (s, 2F, J = 6.3 Hz, J = 3.2 Hz). [13]**C{**[1]**H}-NMR** (125 MHz, DMSO-d$_6$): δ = 156.5, 155.6 (br), 153.6 (br), 151.8, 151.7 (br), 149.7 (br), 143.3, 133.6, 131.8- 130.7, 117.1, 113.2, 113.1-112.8, 89.3, 88.3, 82.5, 81.7, 61.7, 26.9, 25.2.

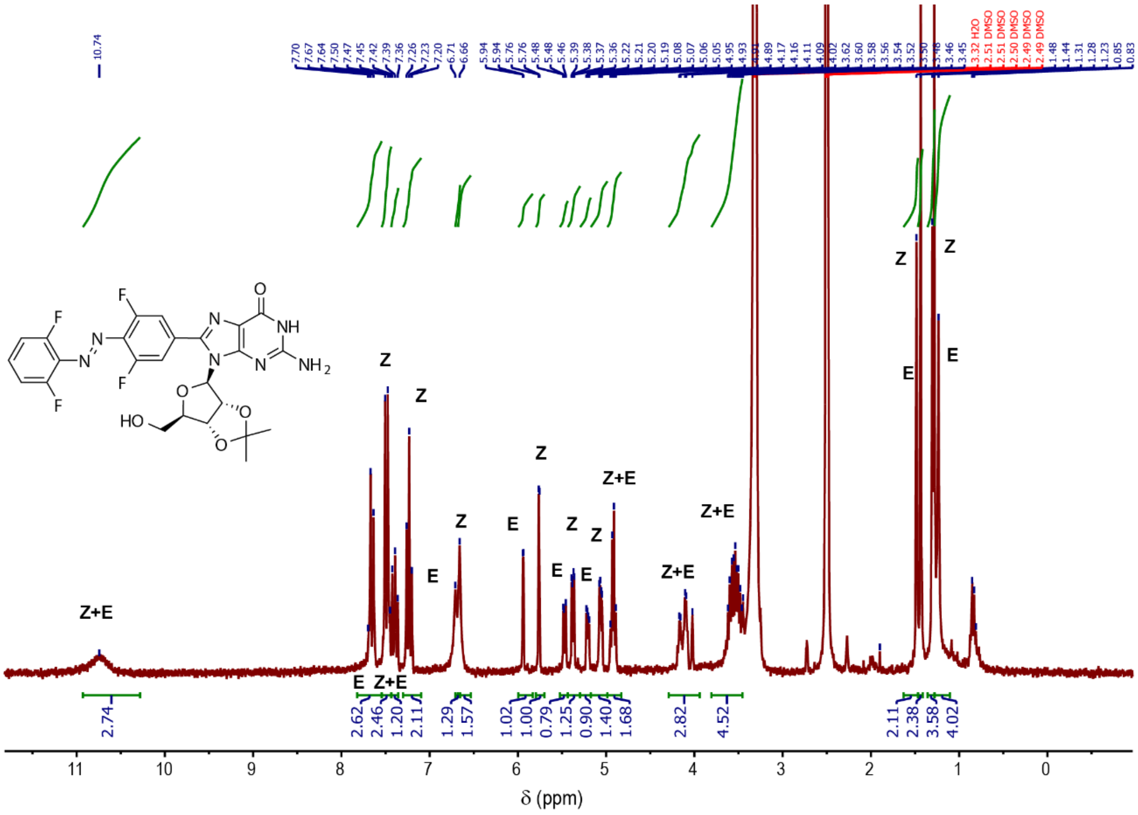

Figure S0E. ^{1}H NMR spectrum of **5** (300 MHz, DMSO-d$_6$).

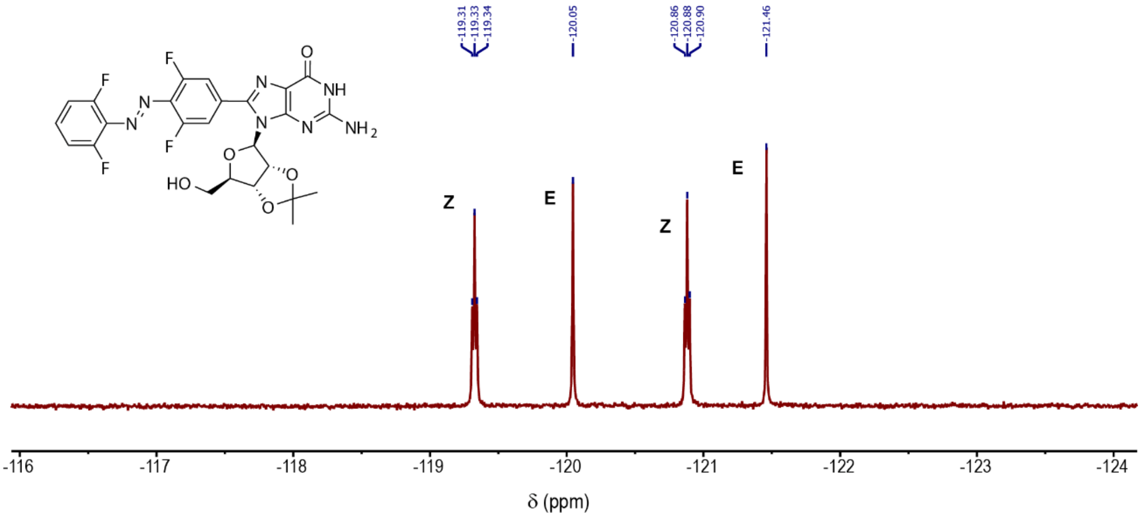

Figure S0F. ^{19}F NMR spectrum of **5** (282 MHz, DMSO-d$_6$).

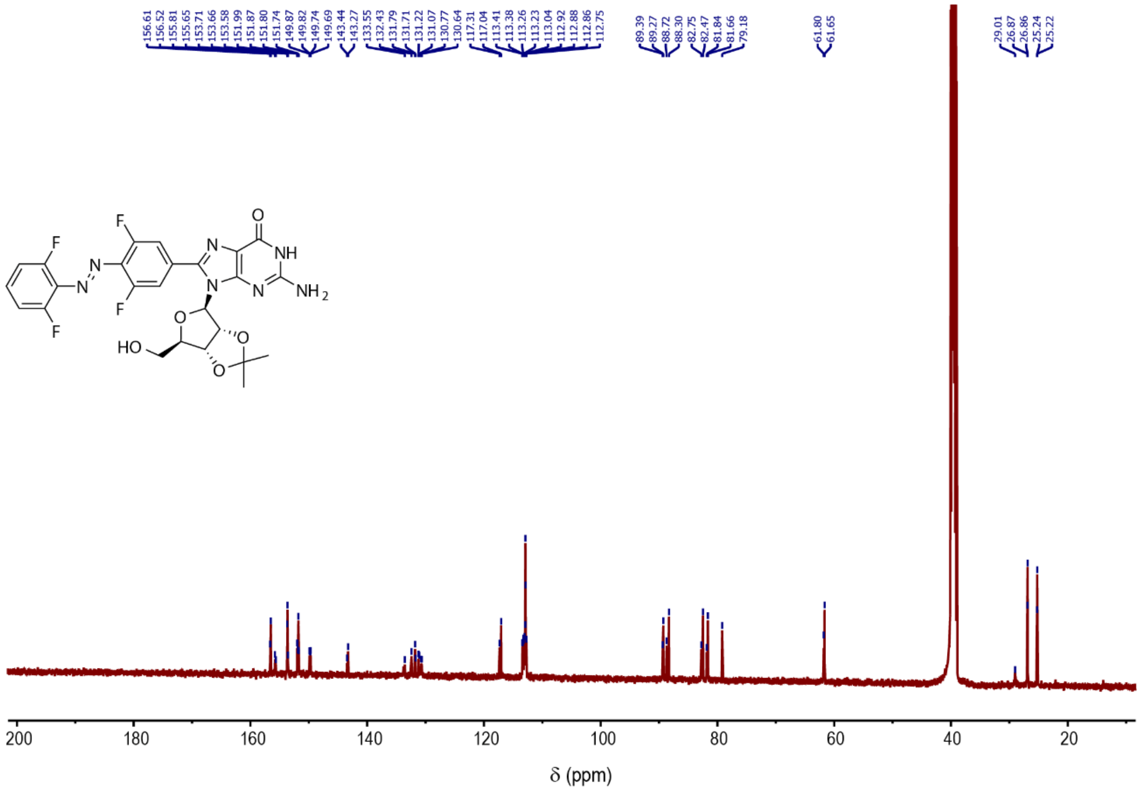

Figure S0G. ^{13}C NMR spectrum of **5** (125 MHz, DMSO-d$_6$).

Synthesis of G2

G2 was obtained by partially reproducing previously described synthetic protocols.[2] To a suspension of **5** (80 mg, 0.139 mmol, 1 eq.) and imidazole (63 mg, 0.417 mmol, 3 eq.) in anhydrous dichloromethane (5 mL x 75 mg of **5**) *tert*-butyldimethylsilyl chloride (28 mg, 0.417 mmol, 3 eq.) is added and the reaction is stirred overnight at 30 ºC with an oil bath. Afterwards, the reaction is stirred at 80 ºC overnight under Ar atmosphere. After TLC unveils the end of the reaction, the crude is washed with HCl 0.01 M, saturated $NaHCO_3$ and brine. The resulting organic phase is dried with $MgSO_4$, filtered, and the solvent is removed in the rotavapor. The obtained crude is purified by silica gel column chromatography (using $CHCl_3$/MeOH 10:0.4 mixture as eluent), providing **G2** (as a mixture of *E* and *Z* isomers, 70 mg, yield 73%) as orange solid.

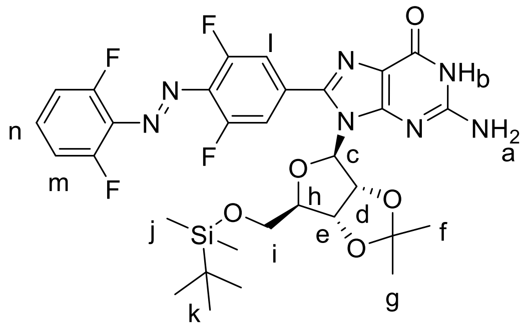

(*E*)-G2. ^{1}**H-NMR** 300 MHz, DMSO-d_6): 10.91 (br, $1H_b$), 7.65 (d, $2H_l$, J = 9.9 Hz), 7.43-7.31 (m, $2H_m$+$1H_n$), 6.72 (br, $2H_a$), 5.97 (d, $1H_c$, J = 1.2 Hz), 5.57 (m, $1H_d$), 5.25 (dd, $1H_e$, J = 6.1 Hz, J = 3.0 Hz), 4.23 (m, $1H_h$), 3.76 (m, $2H_i$), 1.47 (s, $3H_{f\ or\ g}$), 1.31 (s, $3H_{g\ or\ f}$), 0.77 (s, $9H_k$), - 0.14 (s, $6H_j$). ^{19}**F-NMR** (282 MHz, $CDCl_3$): δ = -120.12 (s, 2F), -121.47 (s, 2F). ^{13}**C{^{1}H}-NMR** (125 MHz, DMSO-d_6): δ = 156.7, 155.8 (d), 153.7 (br), 151.8, 151.8 (br), 149.8 (br), 143.4, 133.8-131.2, 117.3, 113.4-112.6, 89.5, 83.0, 82.0, 63.9, 26.8, 25.7, 25.2, 18.0, -5.6. **MS** (MALDI+, matrix DCTB): Calculated for $C_{31}H_{35}F_4N_7O_5Si$ $[M+Na]^+$: 712.2303 Found $[M+Na]^+$: 712.2315.

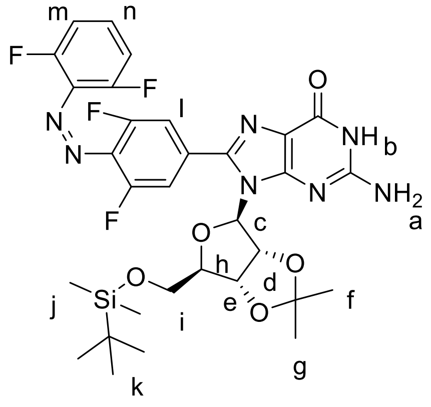

(*Z*)-G2. ^{1}**H-NMR** 300 MHz, DMSO-d_6): 10.91 (br, $1H_b$), 7.49 (d, $2H_l$, J = 9.1 Hz), 7.43-7.31 (m, $1H_n$), 7.22 (dd, $2H_m$, J = 8.7 Hz, J =8.7 Hz), 6.71 (br, $2H_a$), 5.80 (d, $1H_c$, J = 1.3 Hz), 5.52 (dd, $1H_d$, J = 6.1 Hz, J =1.3 Hz), 5.15 (dd, $1H_e$, J = 6.1 Hz, J = 3.0 Hz), 4.15 (m, $1H_h$), 3.69 (m, $2H_i$), 1.45 (s, $3H_{f\ or\ g}$), 1.29 (s, $3H_{g\ or\ f}$), 0.73 (s, $9H_k$), - 0.17 (s, $6H_j$). ^{19}**F-NMR** (282 MHz, DMSO-d_6): δ = -119.16 (t, 2F, J = 4.9 Hz, J =4.9 Hz), -120.91 (t, 2F. J = 4.9 Hz, J =4.9 Hz). ^{13}**C{^{1}H}-NMR** (125 MHz, DMSO-d_6): δ = 156.6, 155.7 (br), 153.6, 151.7 (br), 151.6, 149.7 (br), 143.3, 133.6-131.1 (br), 117.0, 113.2-112.5 (m), 89.3, 82.8, 81.9, 63.7, 26.7, 25.6, 25.2, 17.9, -5.6.

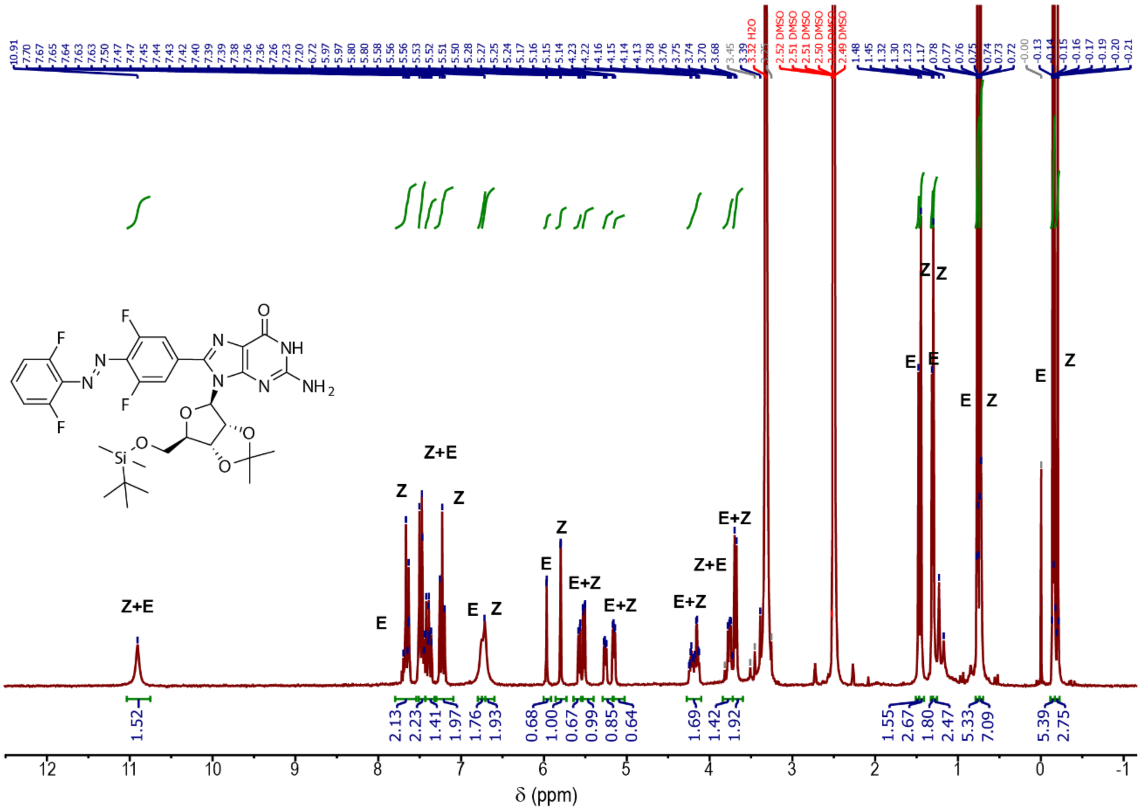

Figure S0H. ^{1}H NMR spectrum of **G2** (300 MHz, DMSO-d_6).

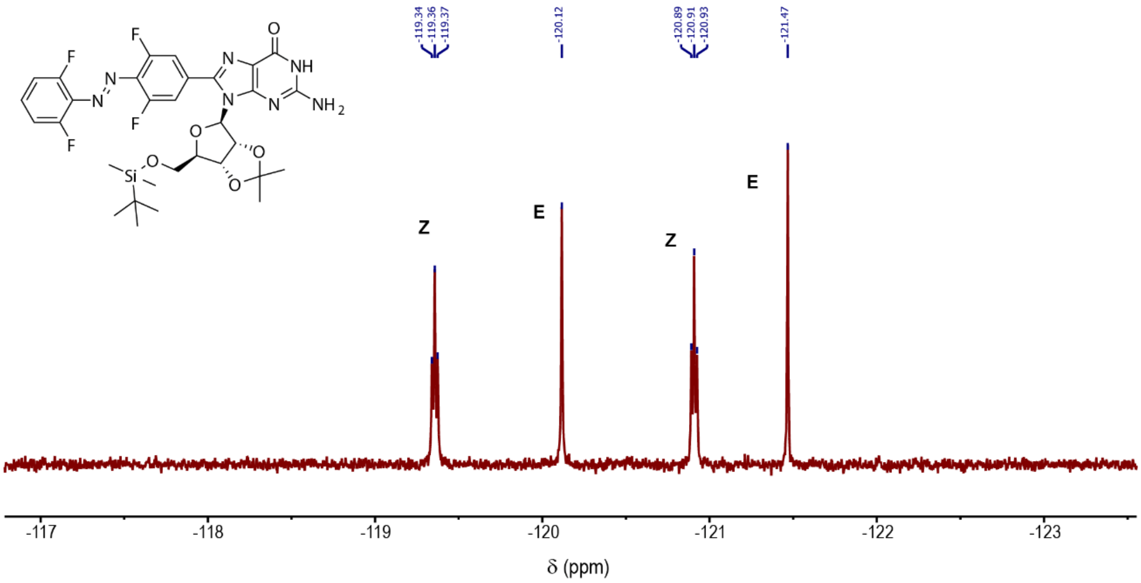

Figure S0I. ^{19}F NMR spectrum of **G2** (282 MHz, DMSO-d_6).

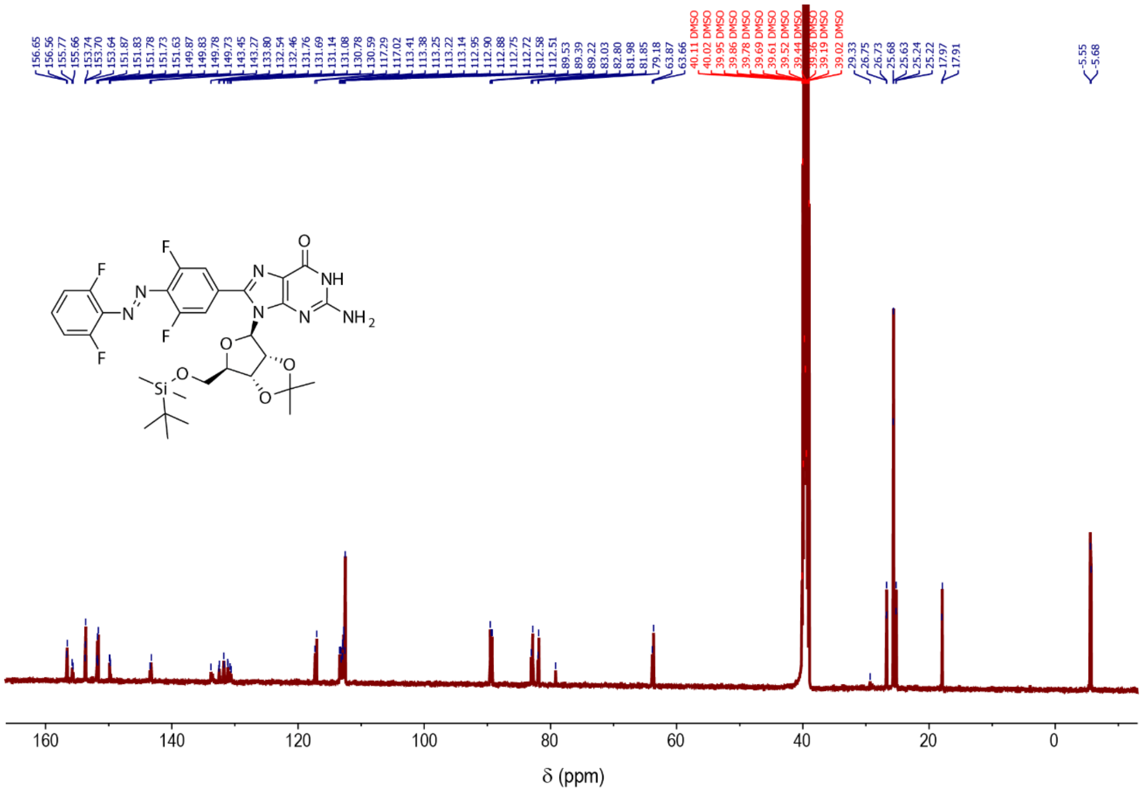

Figure S0J. ^{13}C NMR spectrum of **G2** (125 MHz, DMSO-d$_6$)

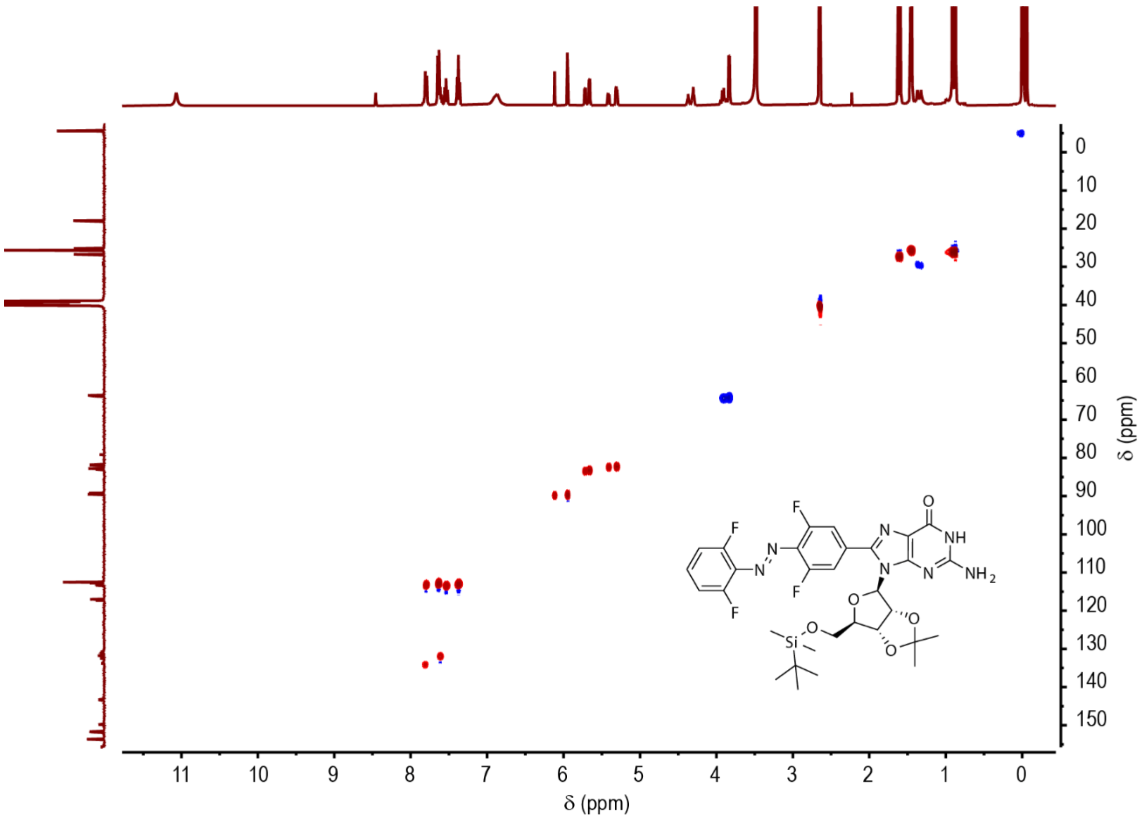

Figure S0K. ^{1}H-^{13}C HSQC spectrum of **G2** (500 MHz, DMSO-d$_6$)

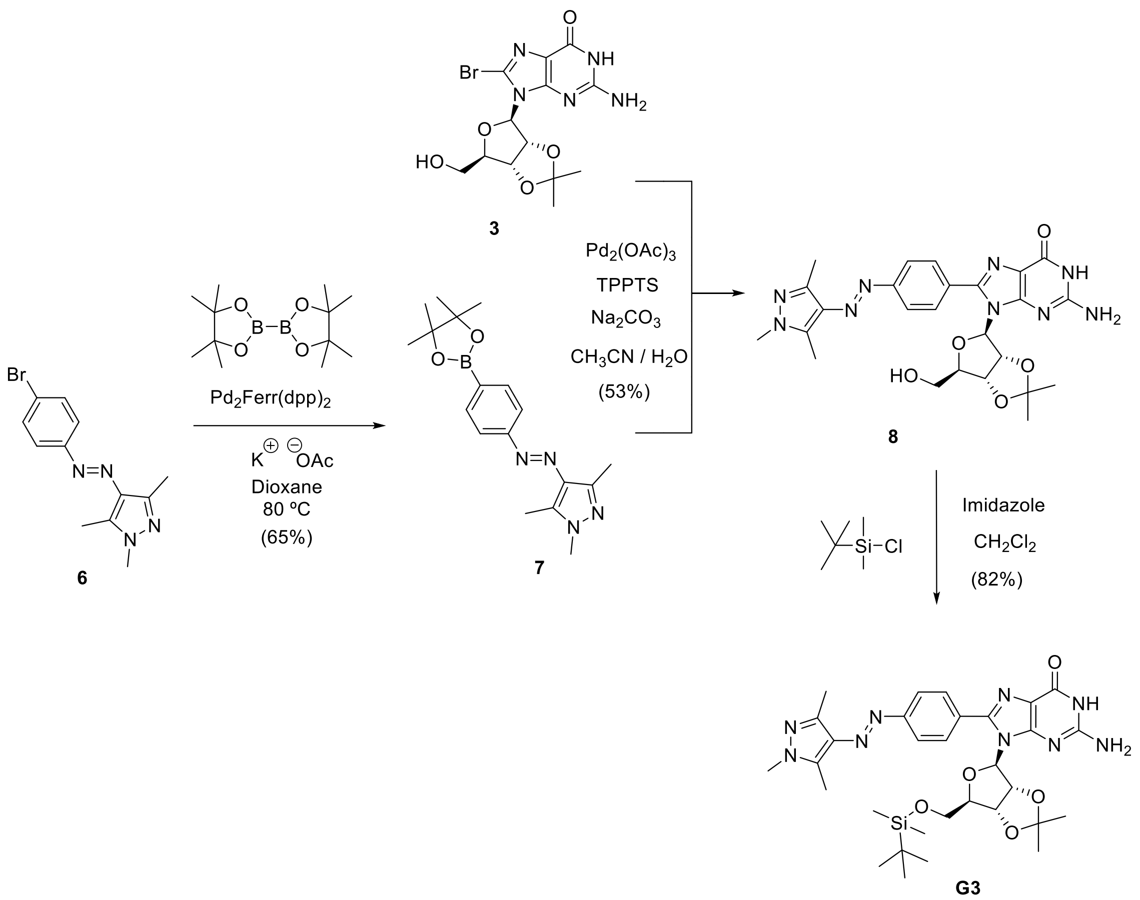

Scheme S0C. Synthetic route to **G3. 3**[2] and **6**[5] were obtained through previously described methods and matched the spectroscopic features reported therein.

Synthesis of boronate ester 7

7 was obtained by partially reproducing previously described synthetic protocols.[3, 6] Bromoazoderivative **6** (600 mg, 2.06 mmol, 1 eq.), bis(pinacolato)diboron (731 mg, 2.88 mmol, 1.4 eq.) and potassium acetate (444 mg, 4.52 mmol, 2.2 eq.) were dissolved in dioxane (20 mL x 0.7 g of **6**) under argon atmosphere. After 15 min, $PdCl_2Ferr(dpp)_2$ (250 mg, 0.30 mmol, 0.15 eq.) is added, and the reaction is stirred overnight at 90 ºC with an oil bath. After that time, dioxane is erased and the crude is redissolved in dichloromethane, and the solution is passed through celite. After removal of the solvent, the organic crude is purified by silica gel chromatography (using heptane/dichloromethane 1:3 mixture as eluents), providing **7** (350 mg, yield 50%) as orange sticky solids.

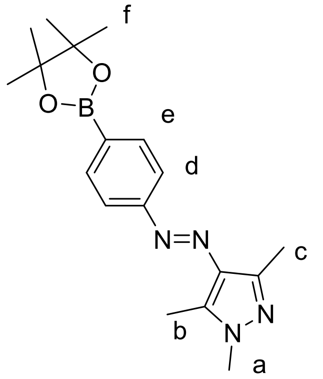

***(E)*-7.** ^{1}**H-NMR** (300 MHz, $CDCl_3$): δ = 7.91 (d, 2H_e, J = 8.5 Hz), 7.76 (d, 2H_d, J = 8.5 Hz), 3.79 (s, 3H_a), 2.59 (s, 3$H_{b\ or\ c}$), 2.51(s, 3$H_{c\ or\ d}$), 1.38 (s, 12H_f). ^{13}**C{**^{1}**H}-NMR** (125 MHz, $CDCl_3$): δ = 155.5, 142.6, 139.0, 135.5, 135.4, 121.0, 83.9, 36.0, 24.9, 13.8, 10.0. HR**MS** (APCI$^+$): Calculated for $C_{18}H_{26}BN_4O_2$ [M+H]$^+$: 341.2149. Found [M+H]$^+$: 341.2140.

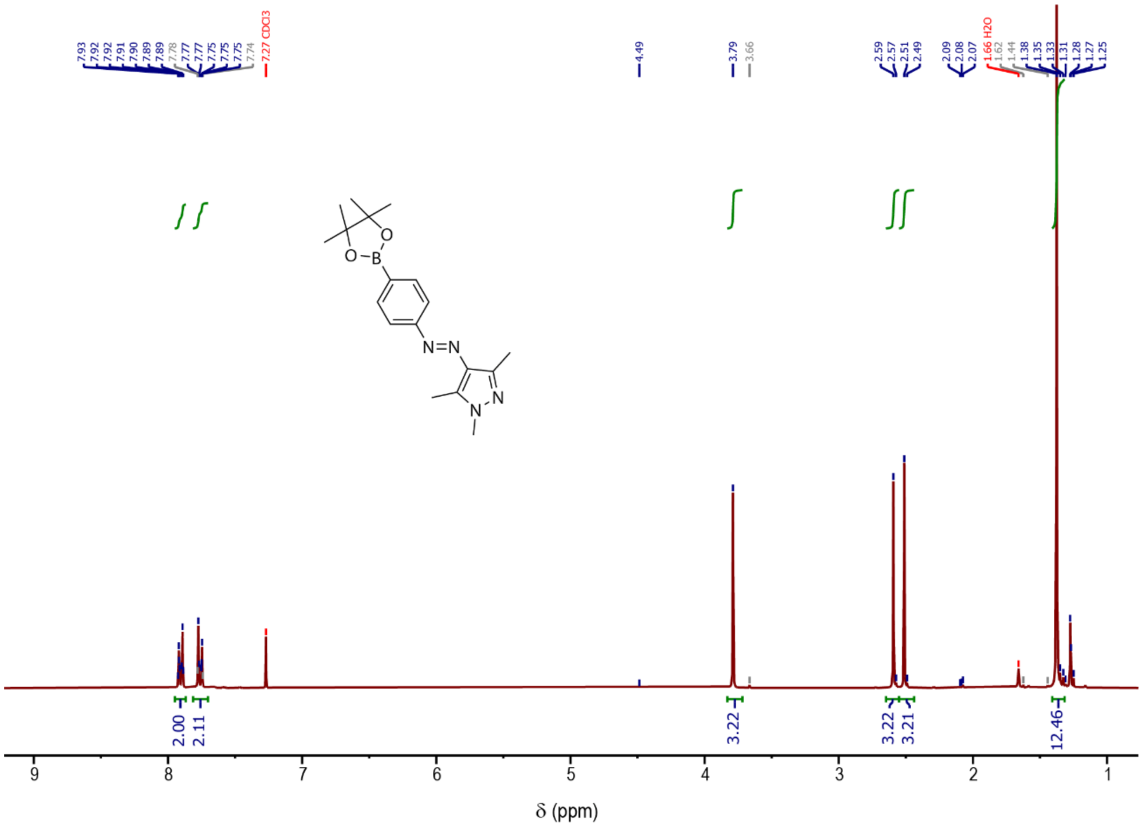

Figure S0L. ^{1}H NMR spectrum of **7** (300 MHz, $CDCl_3$).

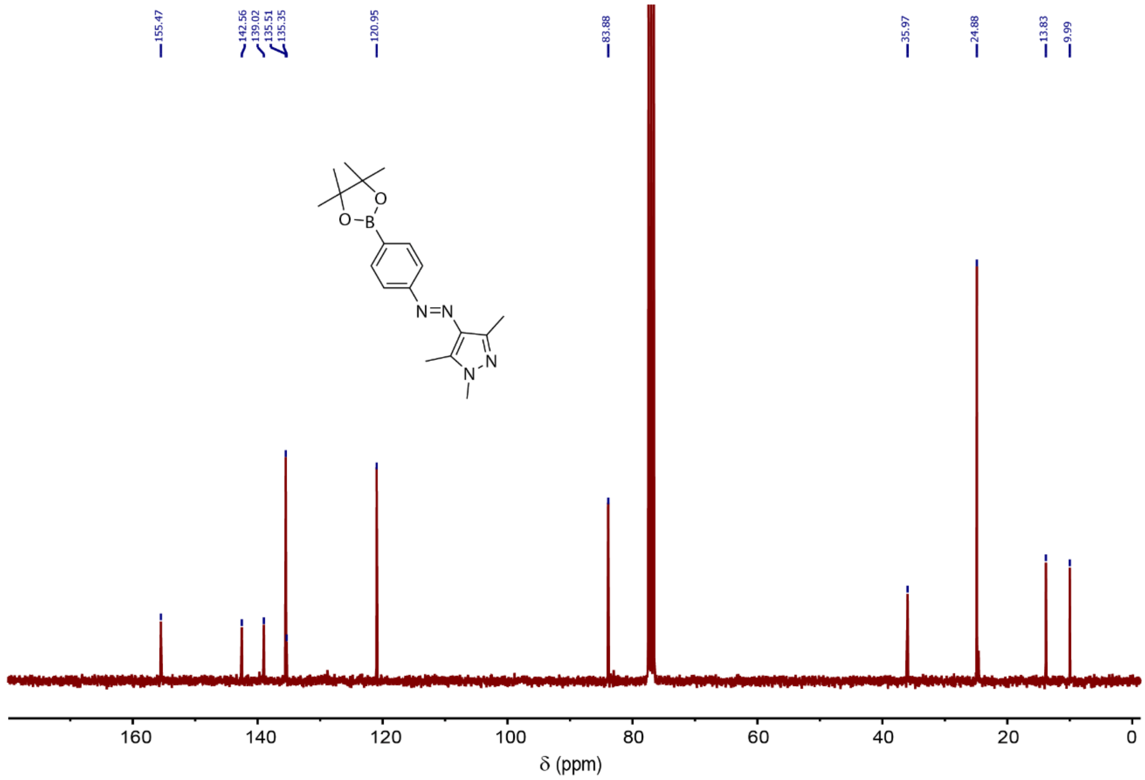

Figure S0M. ^{13}C NMR spectrum of **7** (75 MHz, $CDCl_3$).

Synthesis of G derivative 8

8 was obtained by partially reproducing previously described synthetic protocols.[4] Bromo guanosine derivative **3** (305 mg, 0.759 mmol, 1 eq.), boronate azoderivatives **7** (310 mg, 0.911 mmol, 1.3 eq.), $Pd_2(OAc)_3$ (34 mg, 0.152 mmol, 0.2 eq.) sodium triphenylphosphine trisulfonate (130 mg, 0.228 mmol, 0.3 eq.) and sodium carbonate (0.153 mg, 1.44 mmol, 1.9 eq.) were dissolved in a dioxane/water 1:2 mixture (9 mL x 0.15 g of **2**) and the mixture was degassed during 15 min with Ar. Afterwards, the reaction is stirred at 80 ºC with an oil bath overnight under Ar atmosphere. After that time, water is added and the organic phase is extracted with $CHCl_3$. After washing with brine, the organic phase is dried with $MgSO_4$, filtered, and the solvent is removed in the rotavapor. The resulting crude is purified by silica gel column chromatography (using a gradient from pure $CHCl_3$ to $CHCl_3$/MeOH 10:0.4 mixture as eluent), providing **8** (in the *E* form, 210 mg, yield 52 %) as orange solid.

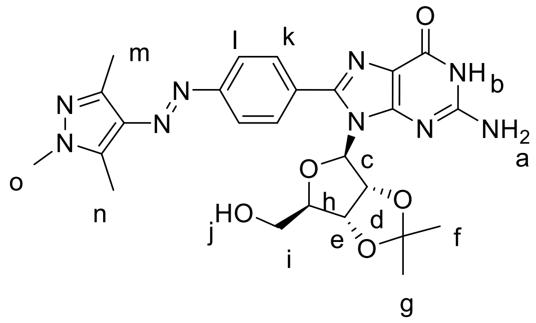

(*E*)-8. Yield 52 %. **^{1}H-NMR** (300 MHz, DMSO-d_6): δ = 10.86 (br, 1H_b), 7.89 (d, 2H_l, *J* = 8.6 Hz), 7.81 (d, 2H_k, *J* = 8.6 Hz), 6.60 (br, 2H_a), 5.85 (d, 1H_c, *J* = 2.0 Hz), 5.41 (dd, 1H_d, *J* = 6.3 Hz, *J* = 2.0 Hz), 5.19 (dd, 1H_e, *J* = 6.3 Hz, *J* = 3.5 Hz), 4.94 (q, 1H_j, *J* = 6.2 Hz), 4.12 (td, 1H_h, *J* = 6.2 Hz, *J* = 3.5 Hz), 3.76 (s, 3H_o), 3.70-350 (m, 2H_i), 2.58 (s, 3$H_{m\text{ or }g}$), 2.40 (s, 3$H_{n\text{ or }m}$), 1.44 (s, 3$H_{f\text{ or }g}$), 1.28 (s, 3$H_{g\text{ or }f}$). **^{13}C{^{1}H}-NMR** (125 MHz, DMSO-d_6): δ = 156.7, 153.4, 153.2, 151.6, 145.8, 140.6, 140.1, 134.7, 130.2, 129.8, 121.7, 116.9, 112.8, 89.5, 88.4, 82.9, 81.8, 62.0, 36.0, 27.0, 25.3, 13.8, 9.5. HR**MS** (APCI$^+$): Calculated for $C_{25}H_{29}N_9O_5$ $[M]^+$: 535.2292. Found $[M]^+$: 535.2283.

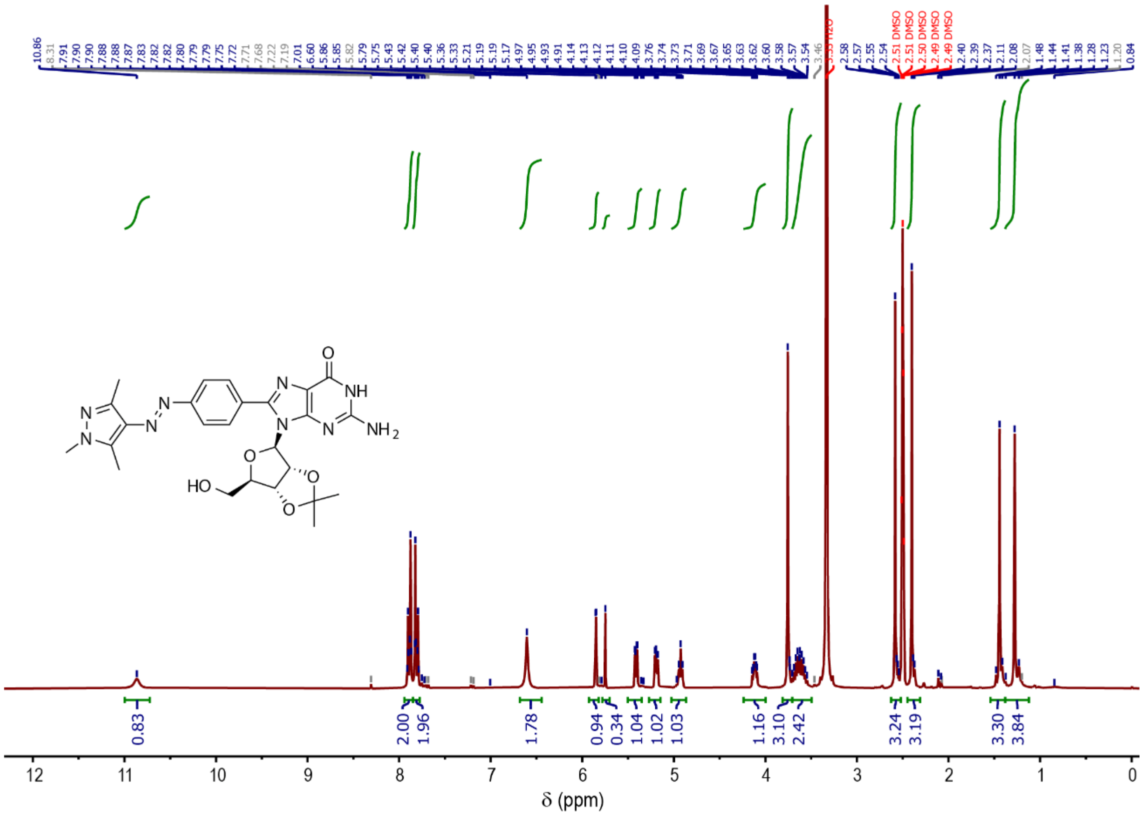

Figure S0N. ^{1}H NMR spectrum of **8** (300 MHz, DMSO-d$_6$)

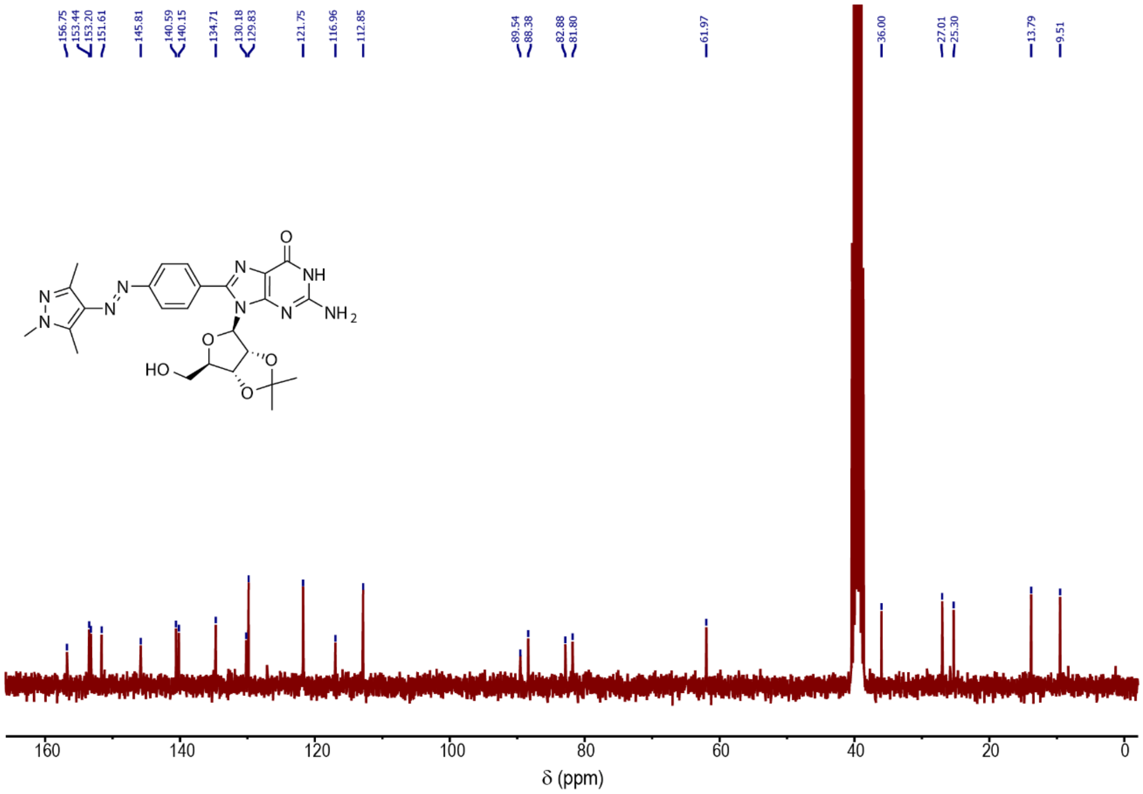

Figure S0O. ^{13}C NMR spectrum of **8** (125 MHz, DMSO-d$_6$).

Synthesis of G3

G3 was obtained by partially reproducing previously described synthetic protocols.[2] To a suspension of **8** (150 mg, 0.280 mmol, 1 eq.) and imidazole (127 mg, 0.840 mmol, 3 eq.) in anhydrous dichloromethane (10 mL x 0.150 g of **8**) *tert*-butyldimethylsilyl chloride (57 mg, 0.840 mmol, 3 eq.) is added and the reaction is stirred overnight at 30 ºC. Afterwards, the reaction is stirred at 80 ºC with an oil bath overnight under Ar atmosphere. After TLC unveils the end of the reaction, the crude is washed with HCl 0.01 M, saturated $NaHCO_3$ and brine. The resulting organic phase is dried with $MgSO_4$, filtered, and the solvent is removed in the rotavapor. The obtained crude is purified by silica gel column chromatography (using $CHCl_3$/MeOH 10:0.4 mixture as eluent), **G3** (in the *E* form, 120 mg, yield 67%) as orange solid.

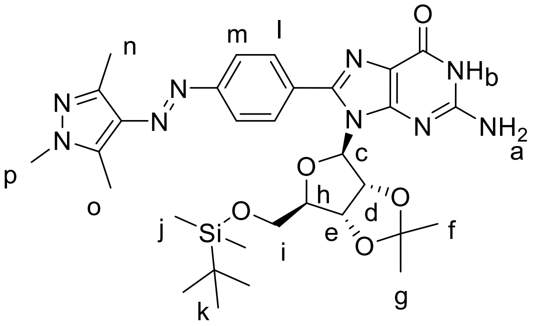

(*E*)-G3. **^{1}H-NMR** (300 MHz, DMSO-d_6): δ = 10.83 (br, 1H_b), 7.89 (d, 2H_l, *J* = 8.8 Hz), 7.81 (d, 2H_m, *J* = 8.8 Hz), 6.63 (br, 2H_a), 5.86 (d, 1H_c, *J* = 1.3 Hz), 5.48 (dd, 1H_d, *J* = 6.3 Hz, *J* = 1.3 Hz), 5.23 (dd, 1H_e, *J* = 6.3 Hz, *J* = 3.3 Hz), 4.15 (m, 1H_h), 3.82 (m, 2H_i), 3.75 (s, 3H_p), 2.58 (s, 3$H_{n\ or\ o}$), 2.40 (s, 3$H_{o\ or\ n}$), 1.44 (s, 3$H_{f\ or\ g}$), 1.29 (s, 3$H_{g\ or\ f}$), 0.78 (s, 9H_k), - 0.13 (d, 6H_j, *J* = 6.3 Hz). **^{13}C{^{1}H}-NMR** (125 MHz, DMSO-d_6): δ = 156.7, 153.3, 153.1, 151.4, 145.8, 140.6, 140.1, 134.7, 130.2, 129.7, 121.7, 116.9, 112.6, 89.7, 89.2, 83.2, 81.9, 64.1, 36.0, 26.8, 25.7, 25.3, 18.0, 13.8, 9.5, -5.5, -5.6. HR**MS** (APCI+): Calculated for $C_{31}H_{44}N_9O_5Si$ $[M+H]^+$: 650.3235. Found $[M+H]^+$: 650.3209.

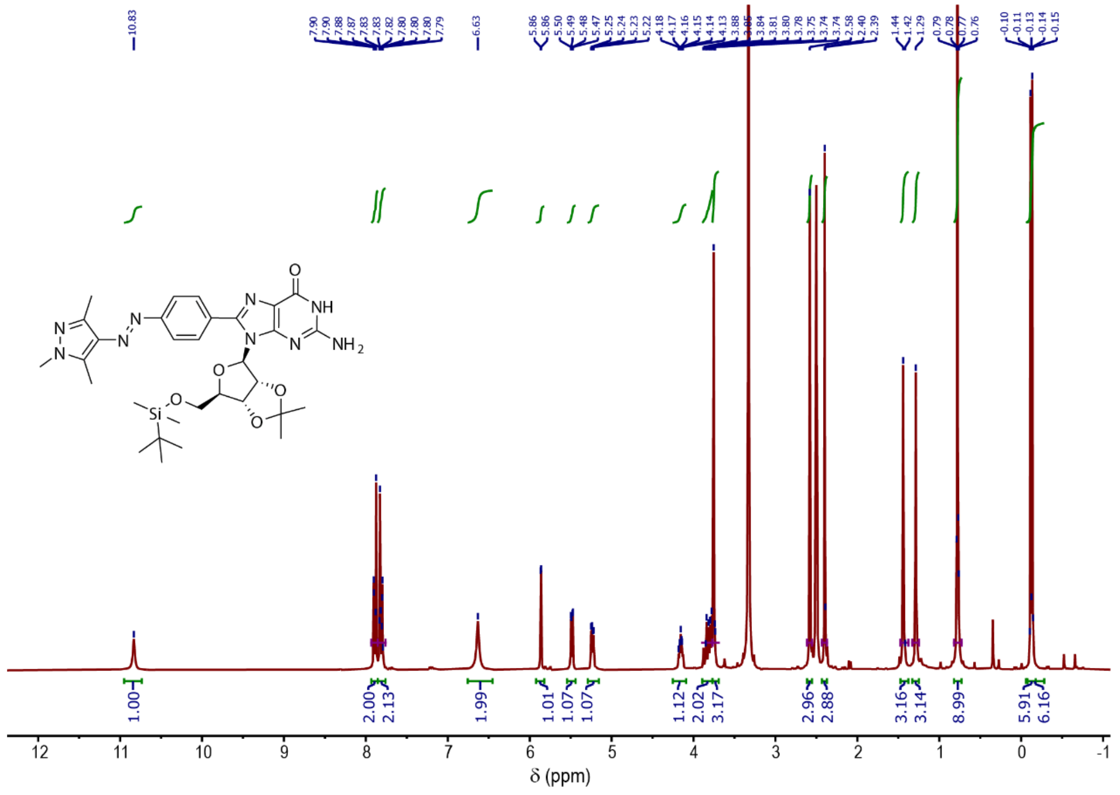

Figure S0P. ^{1}H NMR spectrum of **G3** (300 MHz, DMSO-d_6).

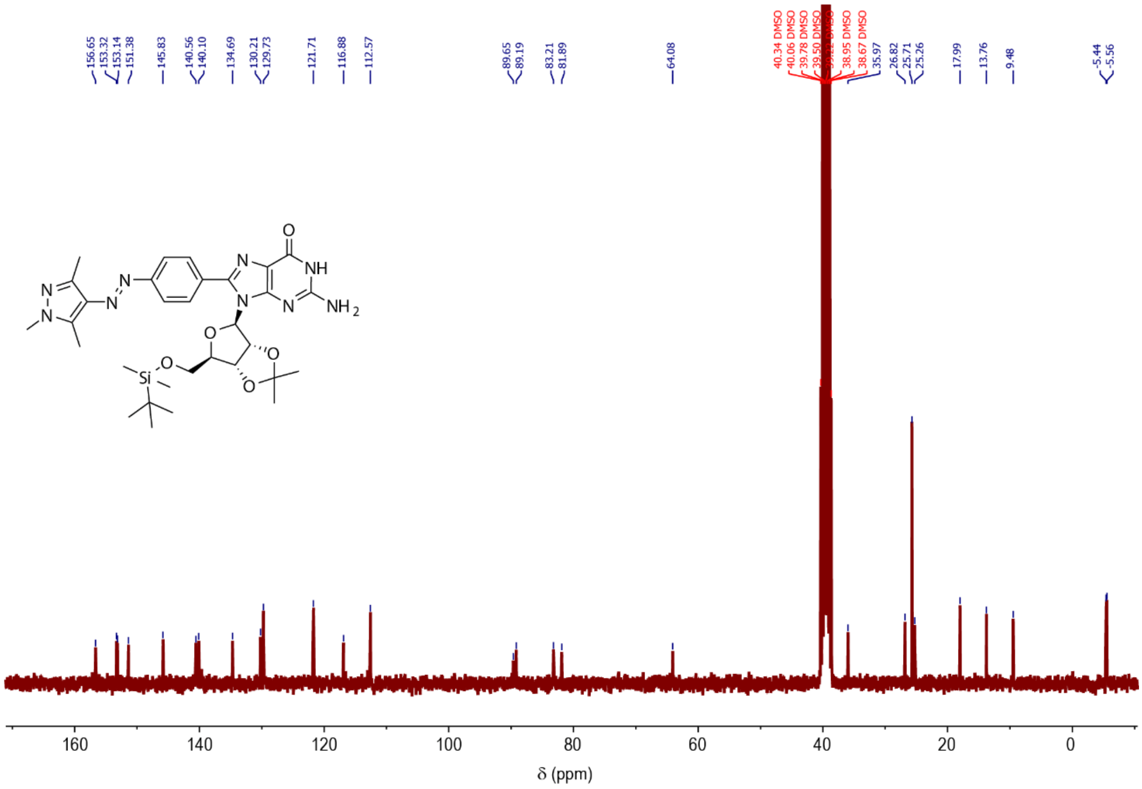

Figure S0Q. ^{13}C NMR spectrum of **G3** (125 MHz, DMSO-d$_6$).

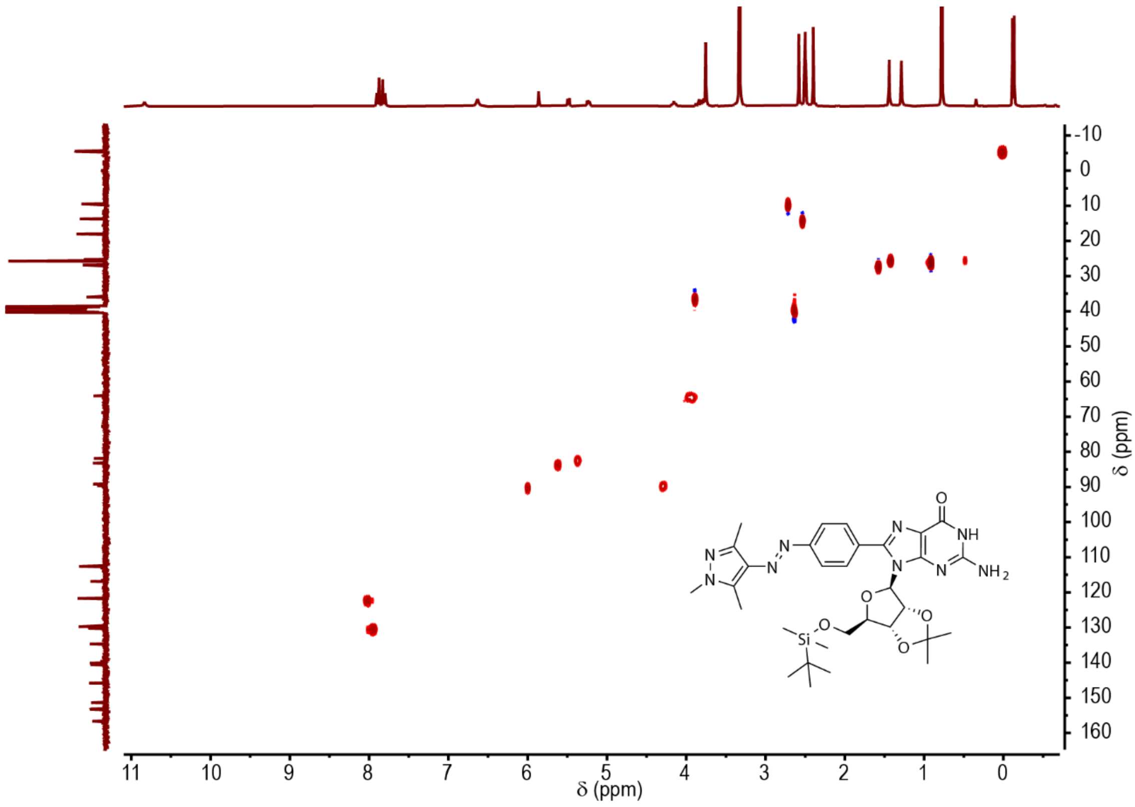

Figure S0R. ^{1}H-^{13}C HSQC spectrum of **G3** (500 MHz, DMSO-d$_6$).

1. Supporting Figures

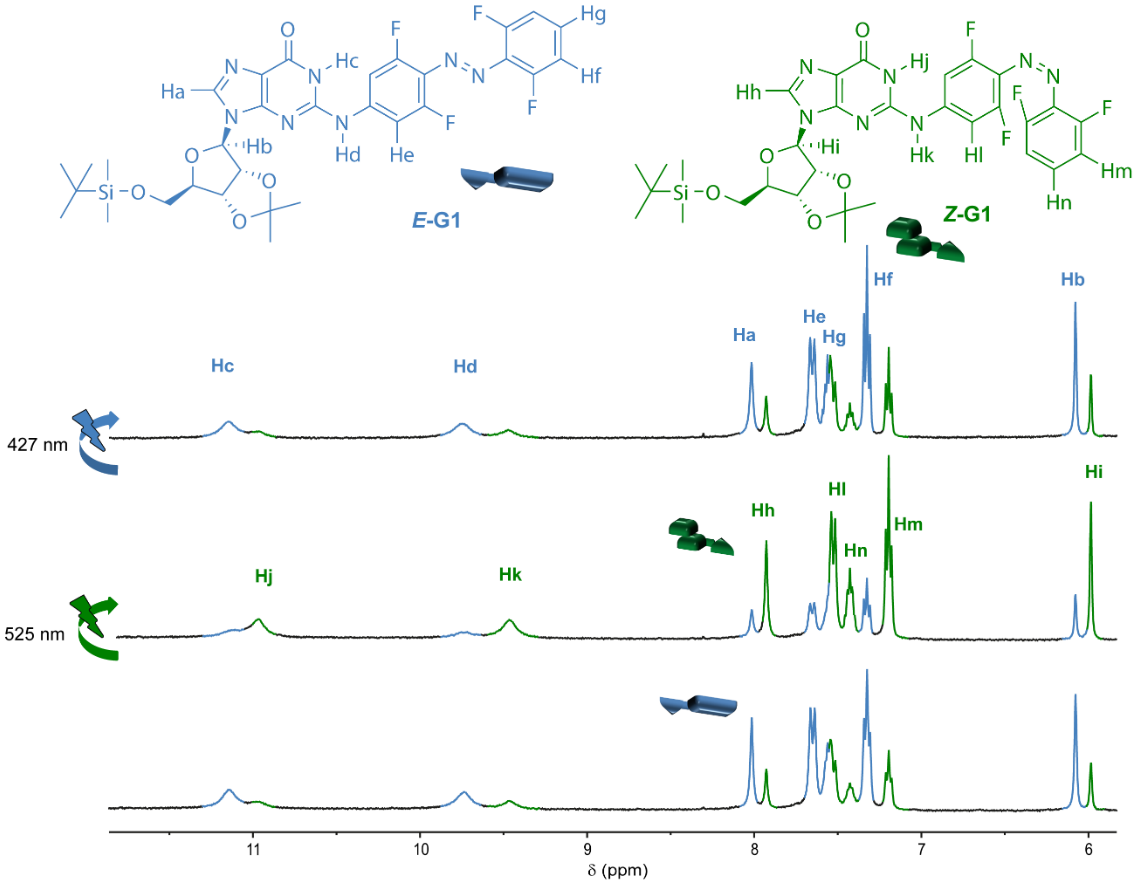

Figure S1. Partial ^{1}H NMR spectra of **G1** (10 mM) in DMSO-d_6 as (bottom spectrum) and after irradiation with 525 nm (middle spectrum) and 427 nm (upper spectrum). Signals in blue and green denote key protons of the *E* and *Z* isomer, respectively.

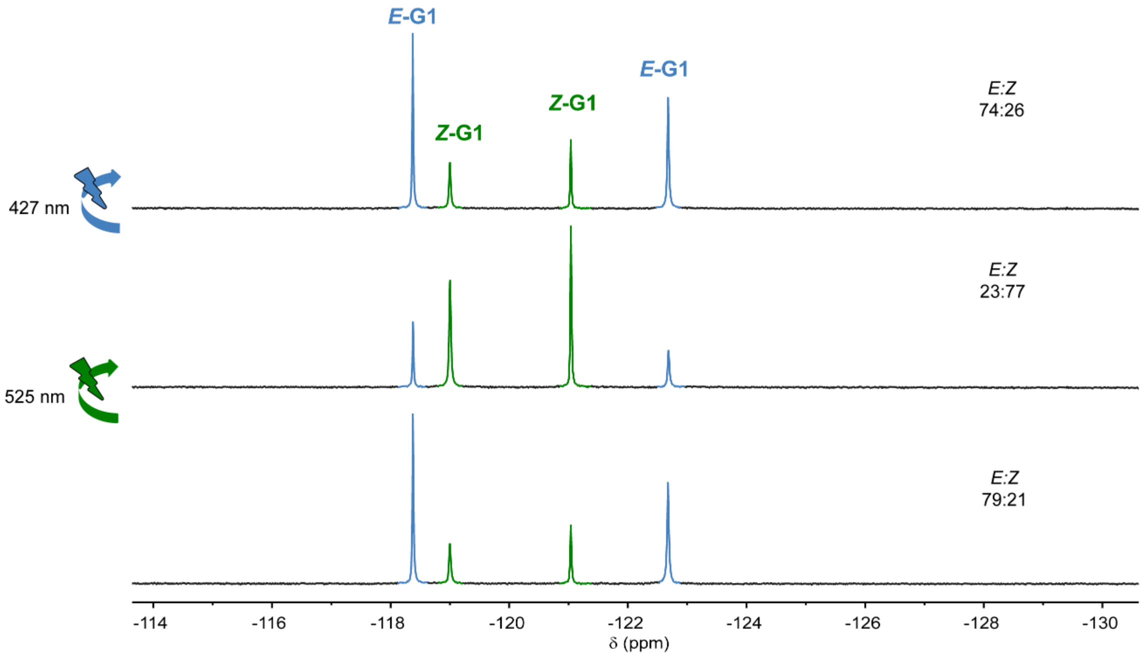

Figure S2. ^{19}F NMR spectra of **G1** (10 mM) in DMSO-d_6 as (bottom spectrum) and after irradiation with 525 nm (middle spectrum) and 427 nm (upper spectrum). Signals in blue and green denote the fluorine atoms of the *E* and *Z* isomer, respectively.

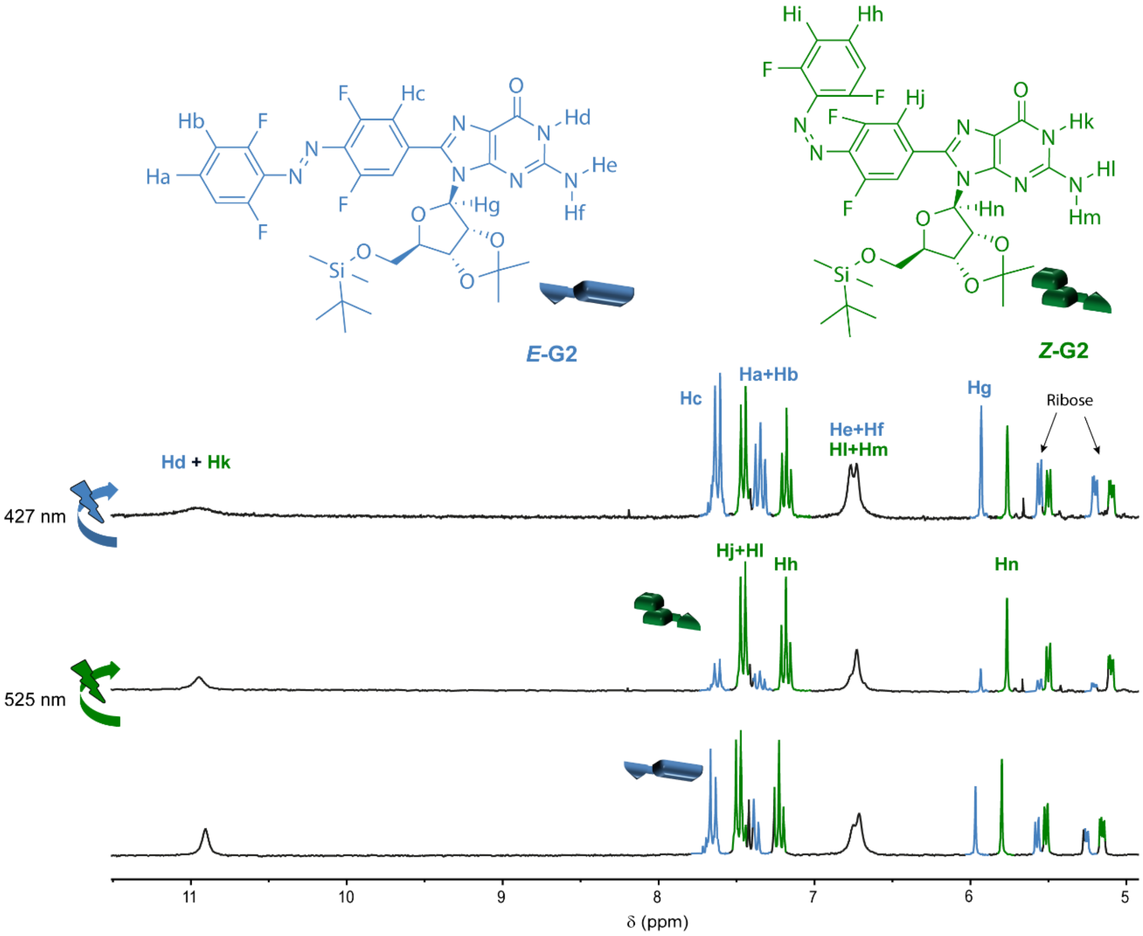

Figure S3. Partial ^{1}H NMR spectra of **G2** (10 mM) in DMSO-d_6 as prepared (bottom spectrum) and after irradiation with 525 nm (middle spectrum) and 427 nm (upper spectrum). Signals in blue and green denote key protons of the *E* and *Z* isomer, respectively.

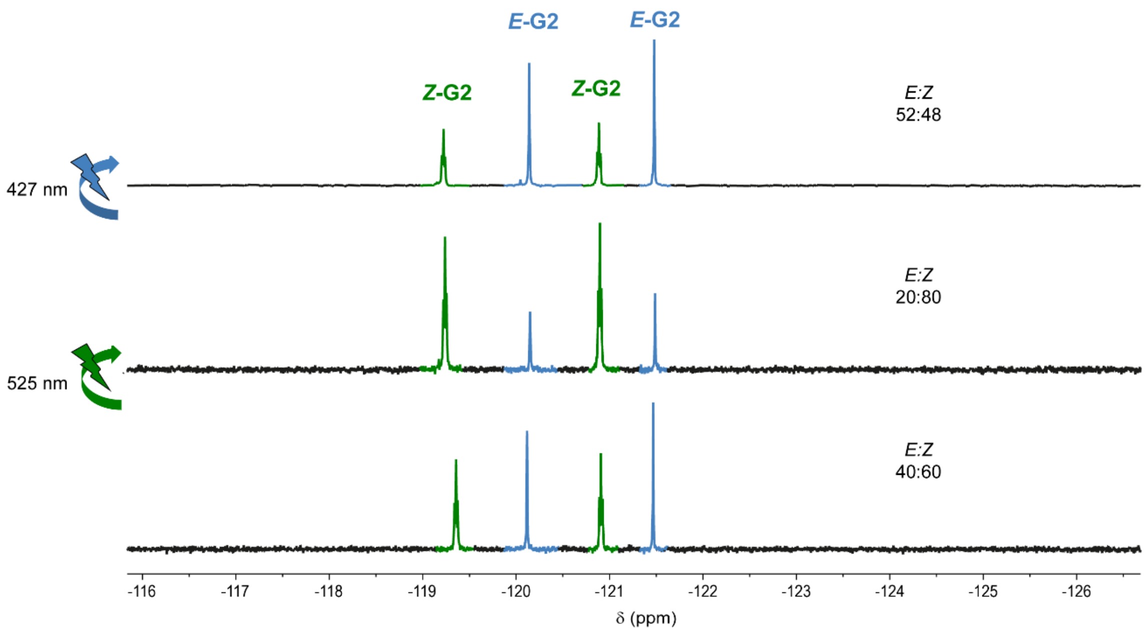

Figure S4. ^{19}F NMR spectra of **G2** (10 mM) in DMSO-d_6 as prepared (bottom spectrum) and after irradiation with 525 nm (middle spectrum) and 427 nm (upper spectrum). Signals in blue and green denote the fluorine atoms of the *E* and *Z* isomer, respectively.

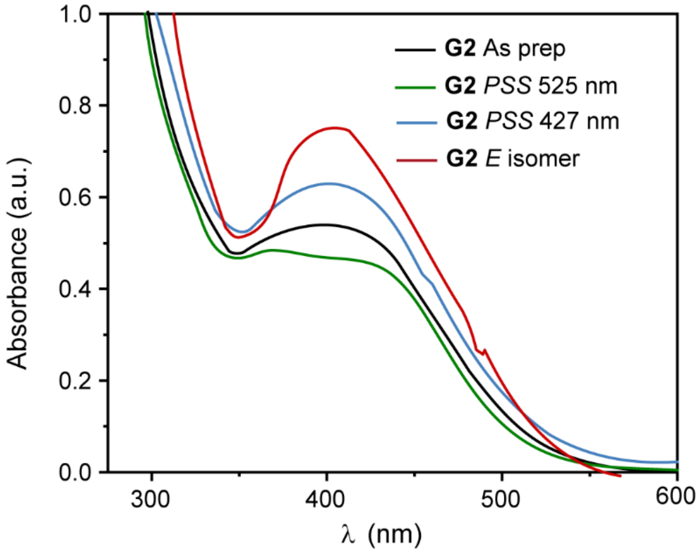

Figure S5. UV Vis spectra of **G2** in DMSO, showing the pristine solution (black lines), after irradiation with 525 nm (green lines), after irradiation with 427 nm (blue lines) and after 6 h at 90ºC (red lines, *E* isomer). [**G2**] = 5.10^{-5} M, (l = 1 mm).

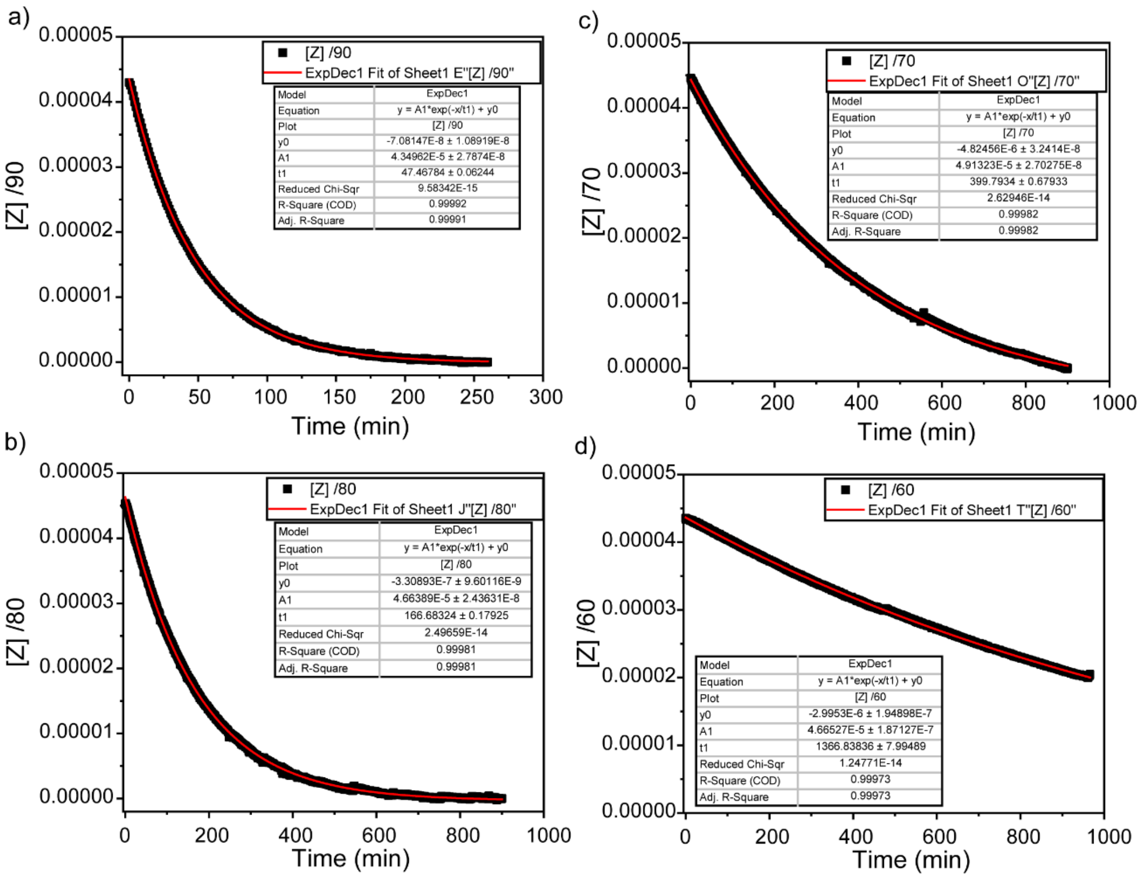

Figure S6. Thermal ***Z*-G2** → ***E*-G2** isomerization in DMSO followed by UV Vis spectroscopy, plotting the *Z* percentages of **G2** at (a) 90ºC, (b) 80 ºC, (c) 70 ºC and (d) 60 ºC. **$[G2]_{tot}$** = 5.10^{-5} M**.** **$[Z\text{-}G2]_0$** was estimated from related NMR experiments.

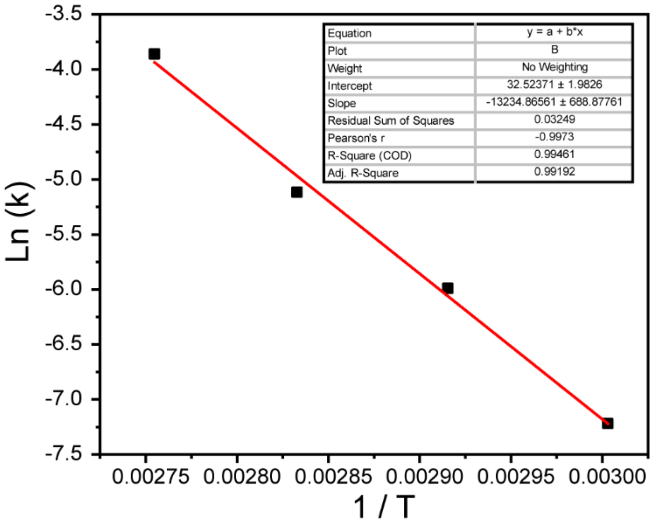

Figure S7. Arrhenius plot of the thermal ***Z*-G2** → ***E*-G2** isomerization in DMSO.

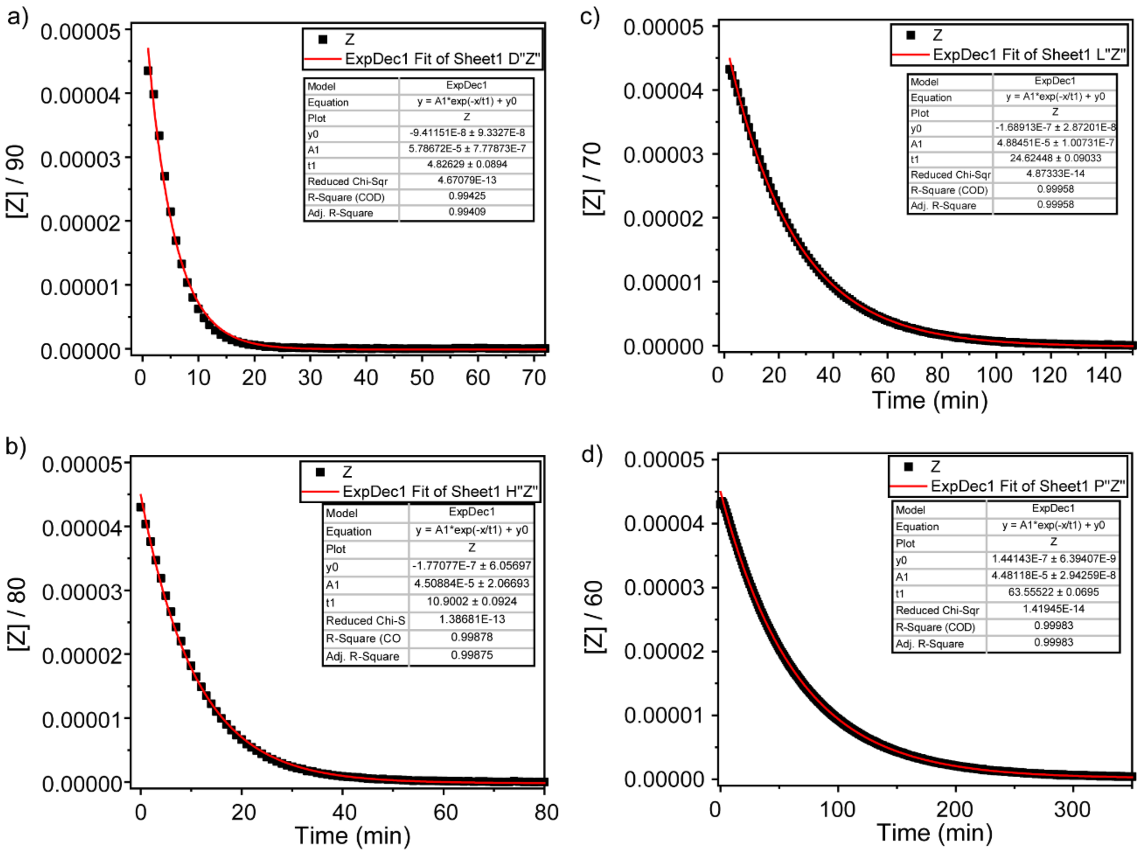

Figure S8. Thermal ***Z*-G3** → ***E*-G3** isomerization in DMSO followed by UV Vis spectroscopy, plotting the *Z* percentages of **G3** at (a) 90ºC, (b) 80 ºC, (c) 70 ºC and (d) 60 ºC. $\mathbf{[G3]_{tot}}$ = 5.10^{-5} M. $\mathbf{[\textit{Z}\text{-}G3]_0}$ was estimated from related NMR experiments.

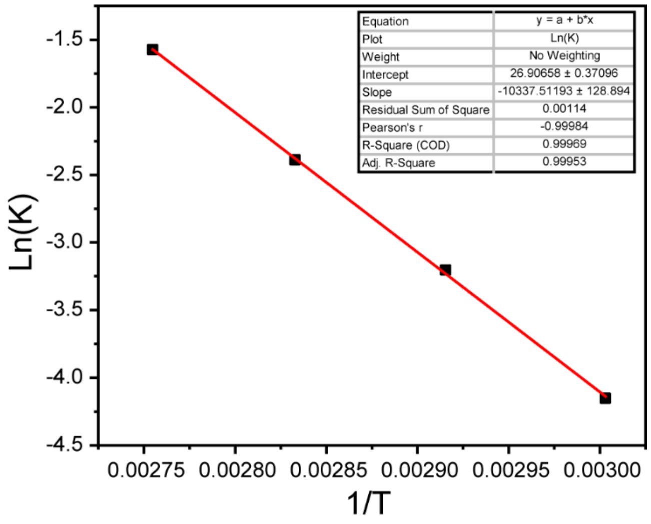

Figure S9. Arrhenius plot of the thermal ***Z*-G3** → ***E*-G3** isomerization in DMSO.

Table S1. Thermal isomerization rates (*k*) and thermal half-lives ($t_{1/2}$) of ***Z*-G2** and ***Z*-G3** at 293 K.

	***k* (293 K) / min^{-1}**	**$t_{½}$ / days**
***Z*-G2**	$3.21.10^{-6}$	149.6
***Z*-G3**	$2.31.10^{-4}$	2.1

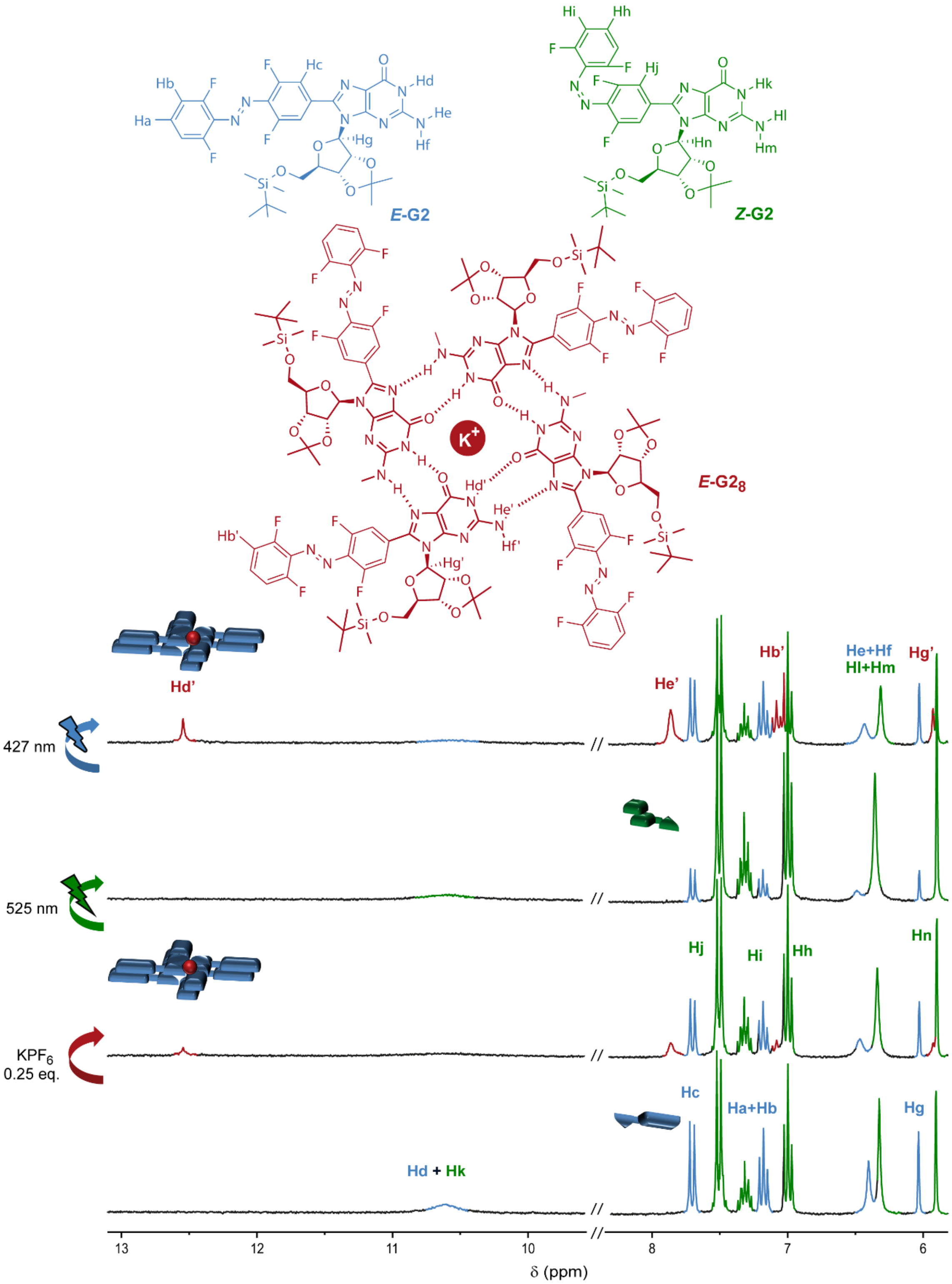

Figure S10. Selected ^{1}H NMR spectra regions of **G2** (10 mM) in THF-d$_8$ as prepared (first bottom spectrum), after the addition 0.25 eq. KPF_6 (second bottom spectrum), after irradiation with 525 nm (second upper spectrum) and after irradiation with 427 nm (first upper spectrum). Signals in red, blue and green denote key protons of the ***E*-G2$_8$** and ***E*-G2** and ***Z*-G2** in the monomeric states, respectively.

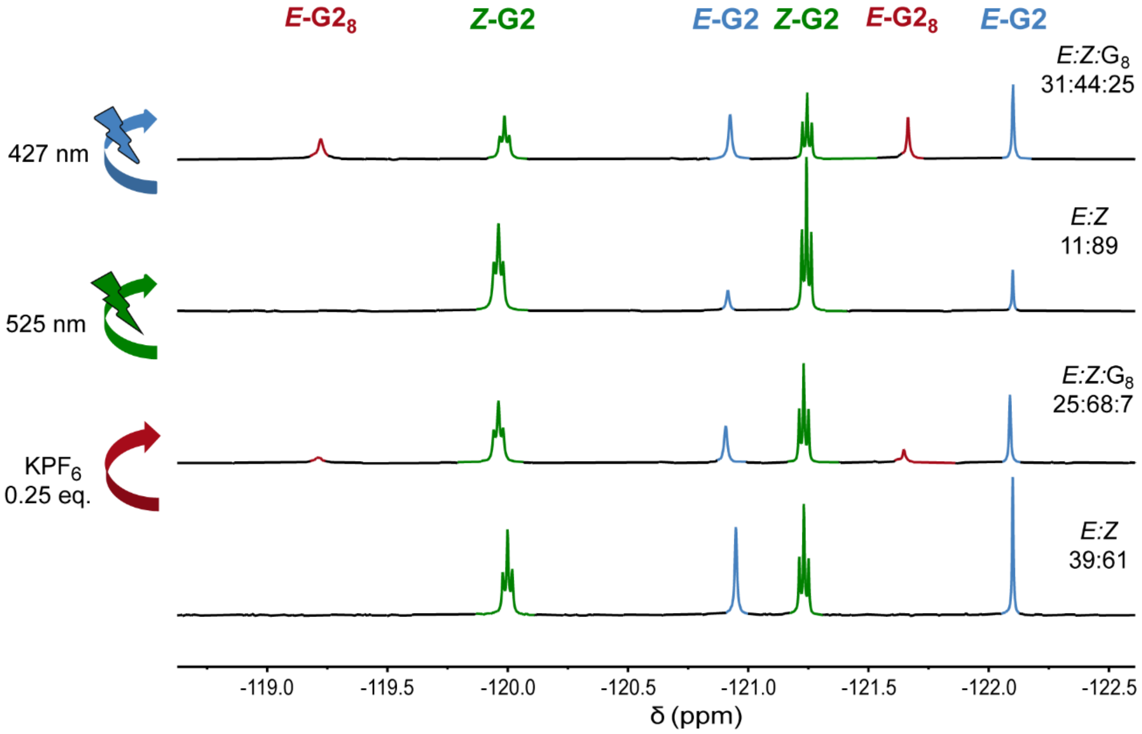

Figure S11. ^{19}F NMR spectra of **G2** (10 mM) in THF-d$_8$ as prepared (first bottom spectrum), after the addition 0.25 eq. KPF_6 (second bottom spectrum), after irradiation with 525 nm (second upper spectrum) and after irradiation with 427 nm (first upper spectrum). Signals in red, blue and green denote the fluorine atoms of the *E*-G-octamer and *E* and *Z* isomer in the monomeric states, respectively.

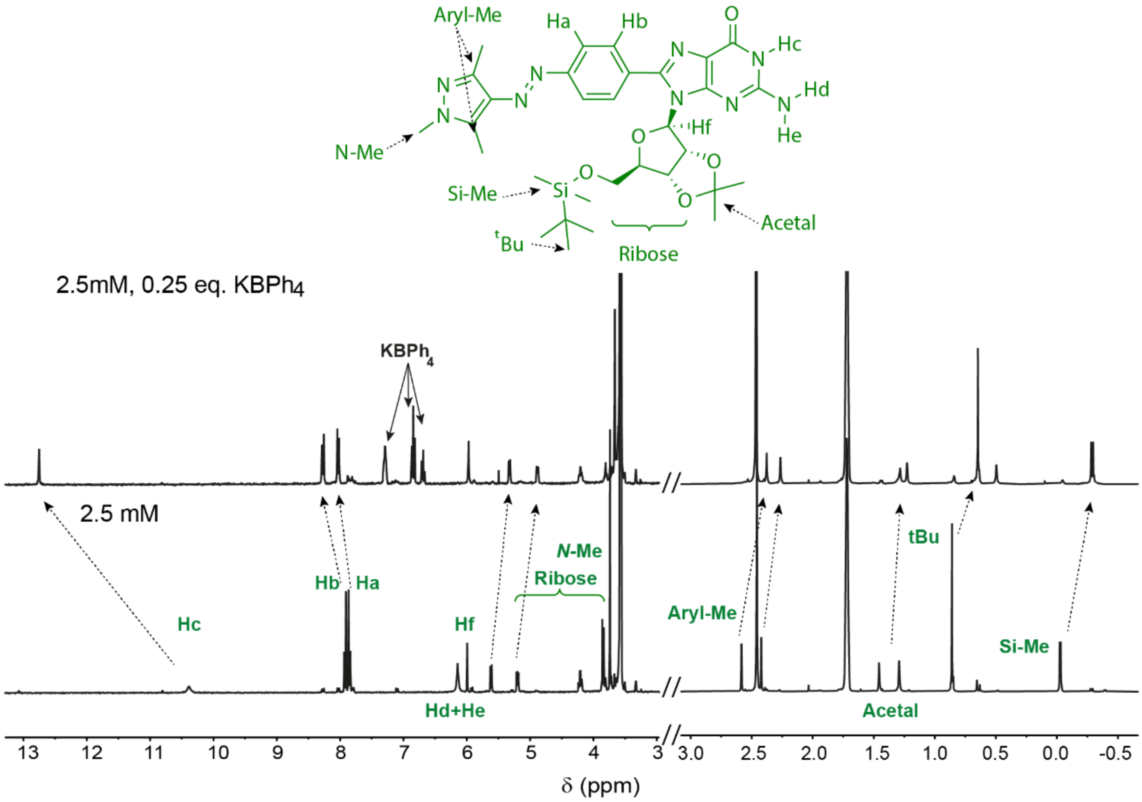

Figure S12. ^{1}H NMR spectra of ***E*-G3** (2.5 mM) in THF-d_8 before (lower spectrum) and after (upper spectrum) the addition of 0.25 eq. of $KBPh_4$, showing the formation of ***E*-G3$_8$**.

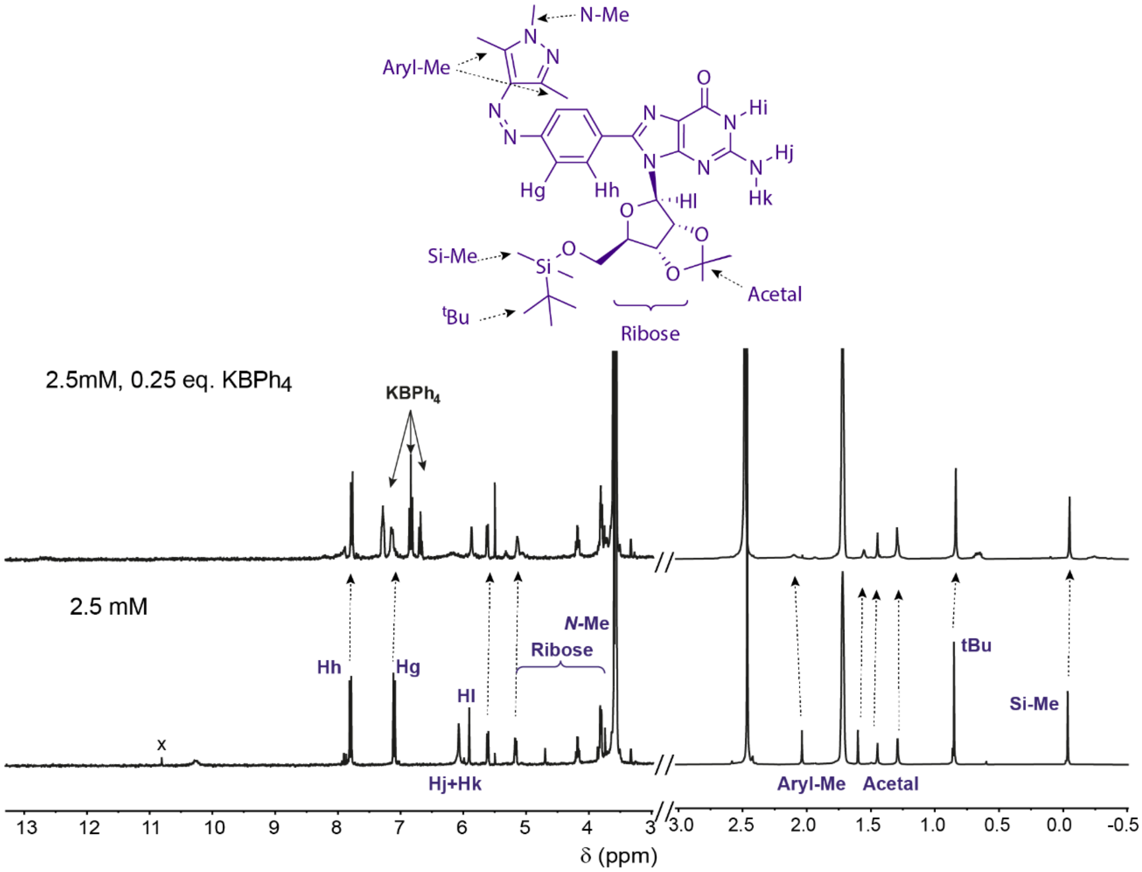

Figure S13. ^{1}H NMR spectra of ***Z*-G3** (2.5 mM) in THF-d_8 before (lower spectrum) and after (upper spectrum) the addition of 0.25 eq. of $KBPh_4$. X denotes the presence of a solvent stabilizer.

Note that in Figure S13, the N-H signal in the monomeric state of the ***Z*-G3** (as well as in Figure S12 for the monomeric state of the ***E*-G3**) is much more visible than in the presence of salt. We hypothesize that this disappearance (broadening) of the N-H signal is due to the formation of an ill-defined mixture of small H-bonded oligomers that exchange in a timescale comparable to that of the NMR technique.

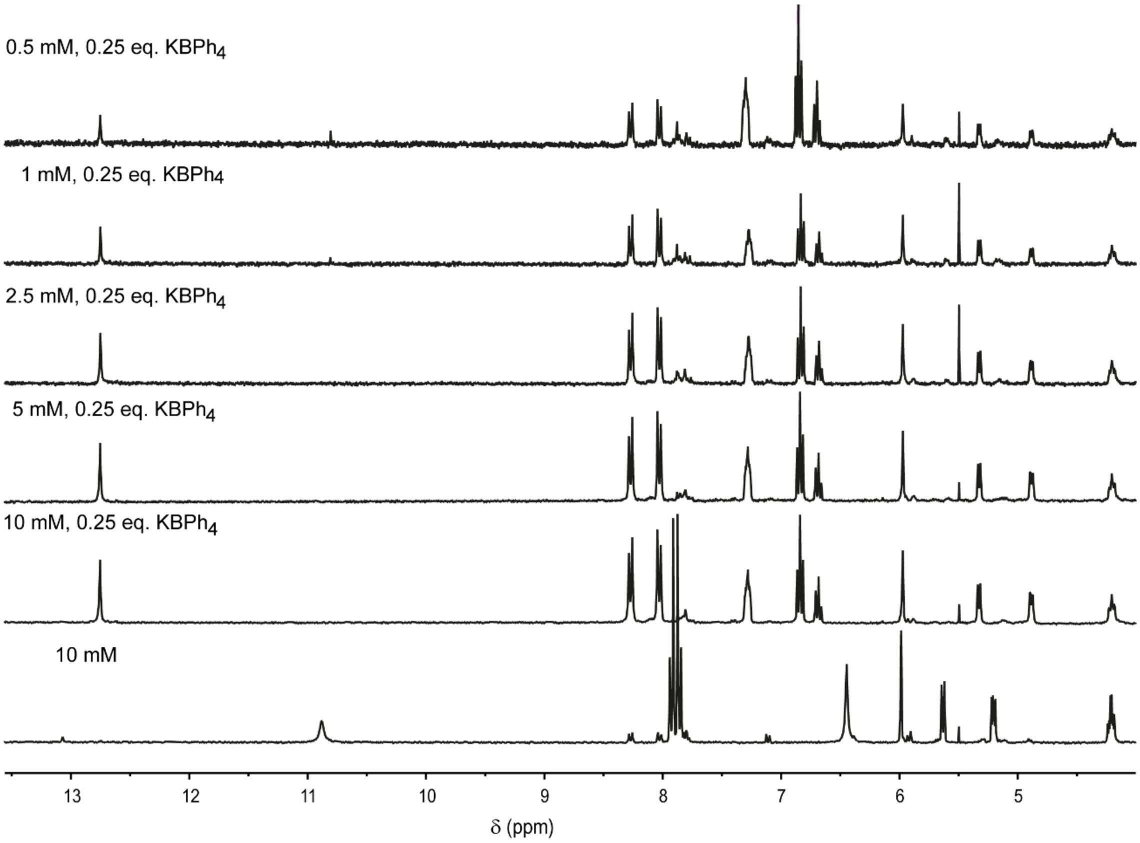

Figure S14. Evolution of the ^{1}H NMR spectra (from lower to upper) of ***E*-G3** in THF-d_8 after the addition of 0.25 eq. of $KBPh_4$ and subsequent changes in concentration.

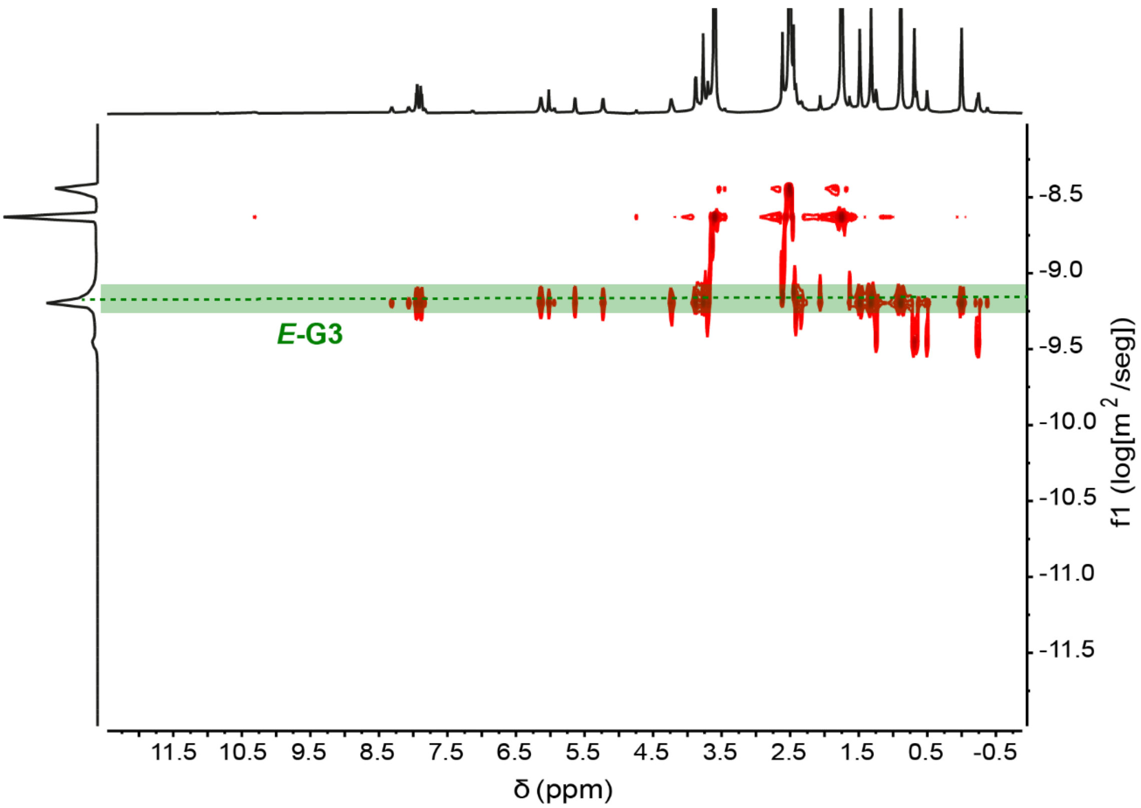

Figure S15. 2D DOSY spectrum of ***E*-G3** in THF-d_8 at 298 K.

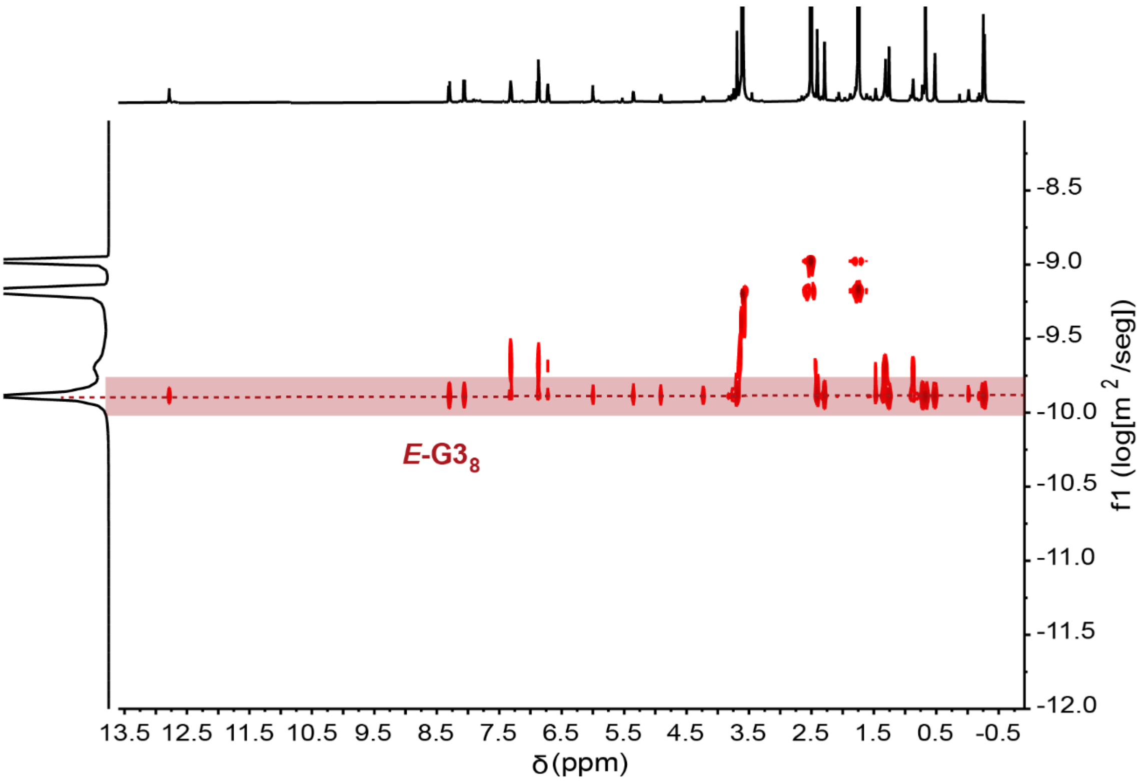

Figure S16. 2D DOSY spectrum of ***E*-G3** in THF-d_8 with 0.25 eq. of $KBPh_4$ at 298 K.

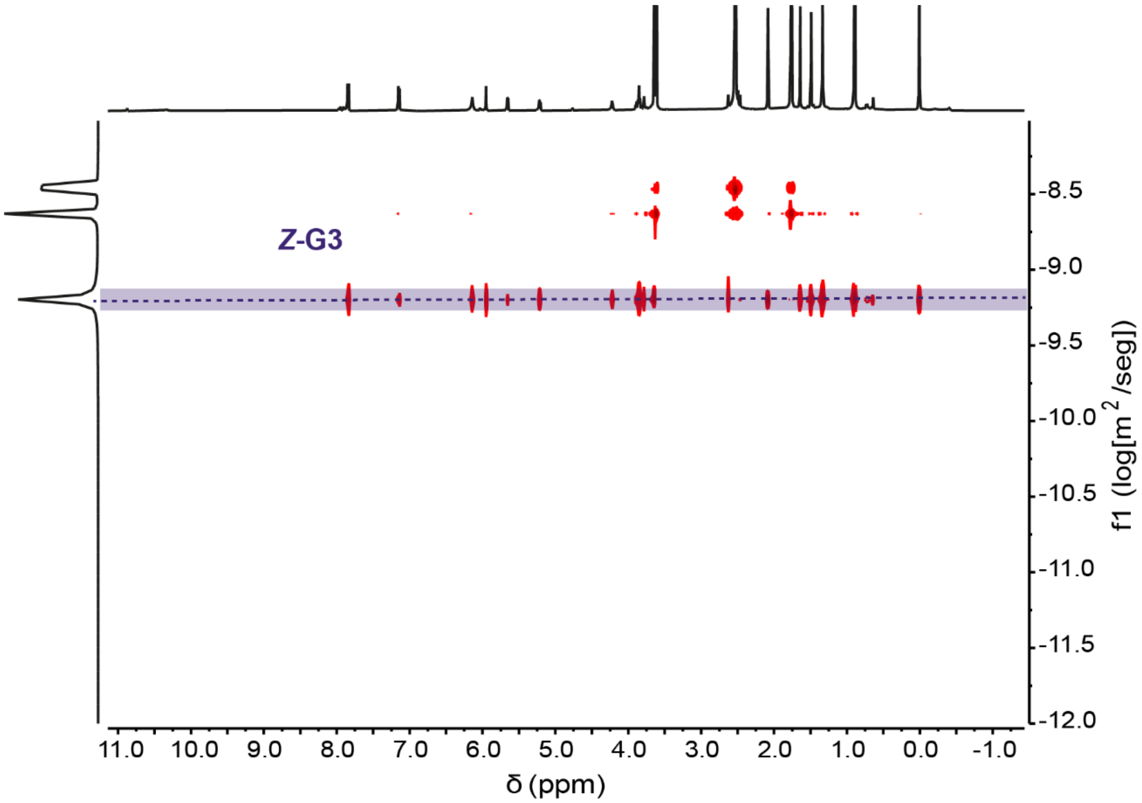

Figure S17. 2D DOSY spectrum of ***Z*-G3** in THF-d_8 at 298 K.

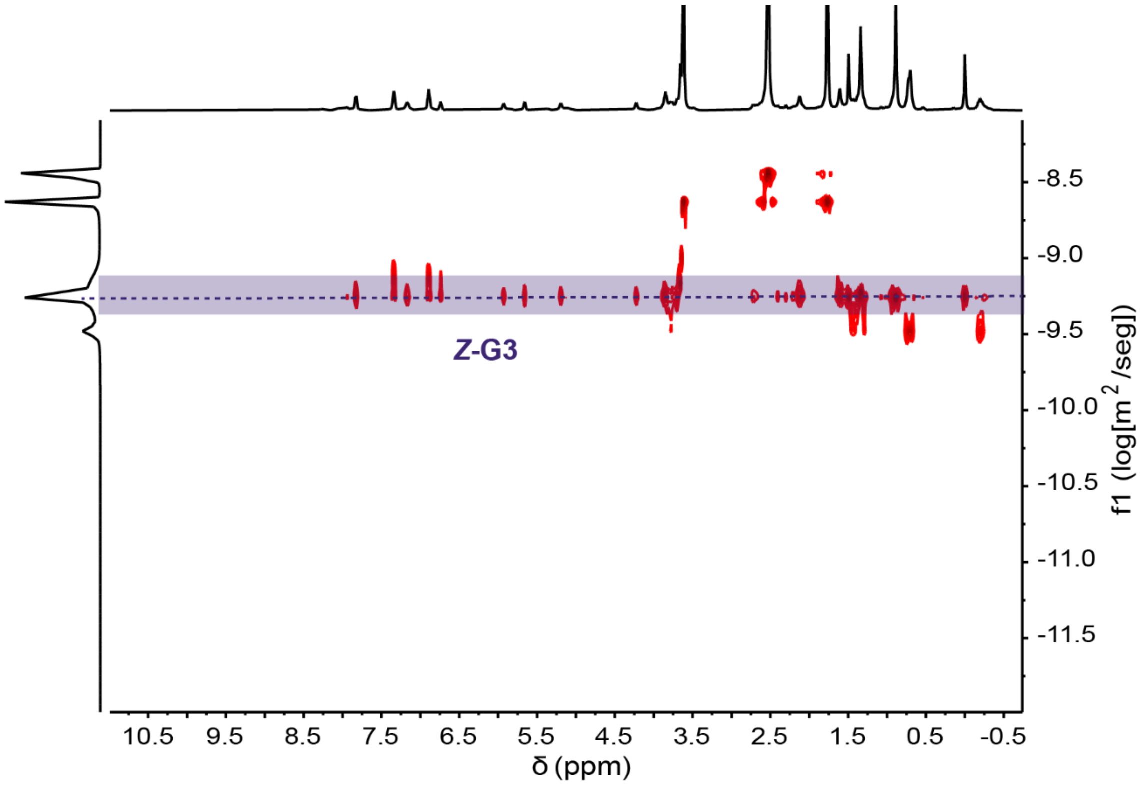

Figure S18. 2D DOSY spectrum of ***Z*-G3** in THF-d_8 with 0.25 eq. of $KBPh_4$ at 298 K.

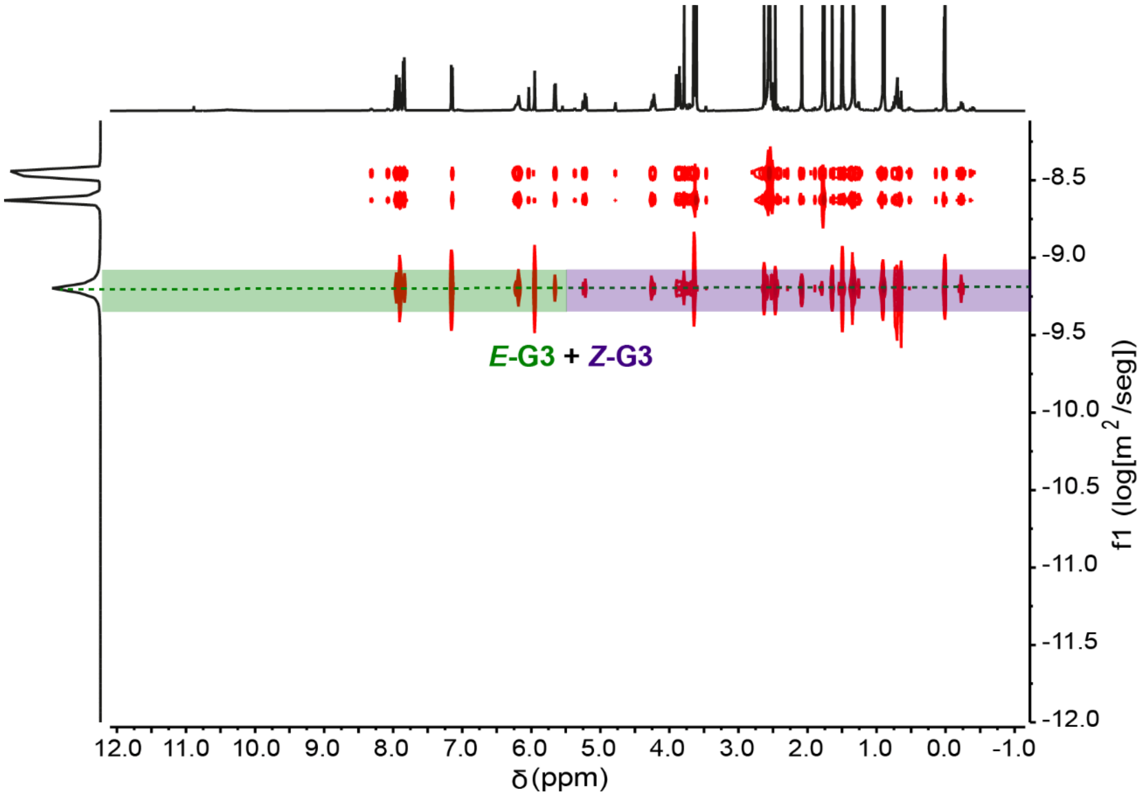

Figure S19. 2D DOSY spectrum of a mixture of the ***E*-G3** and ***Z*-G3** in THF-d_8 at 298 K, showing similar diffusion coefficients.

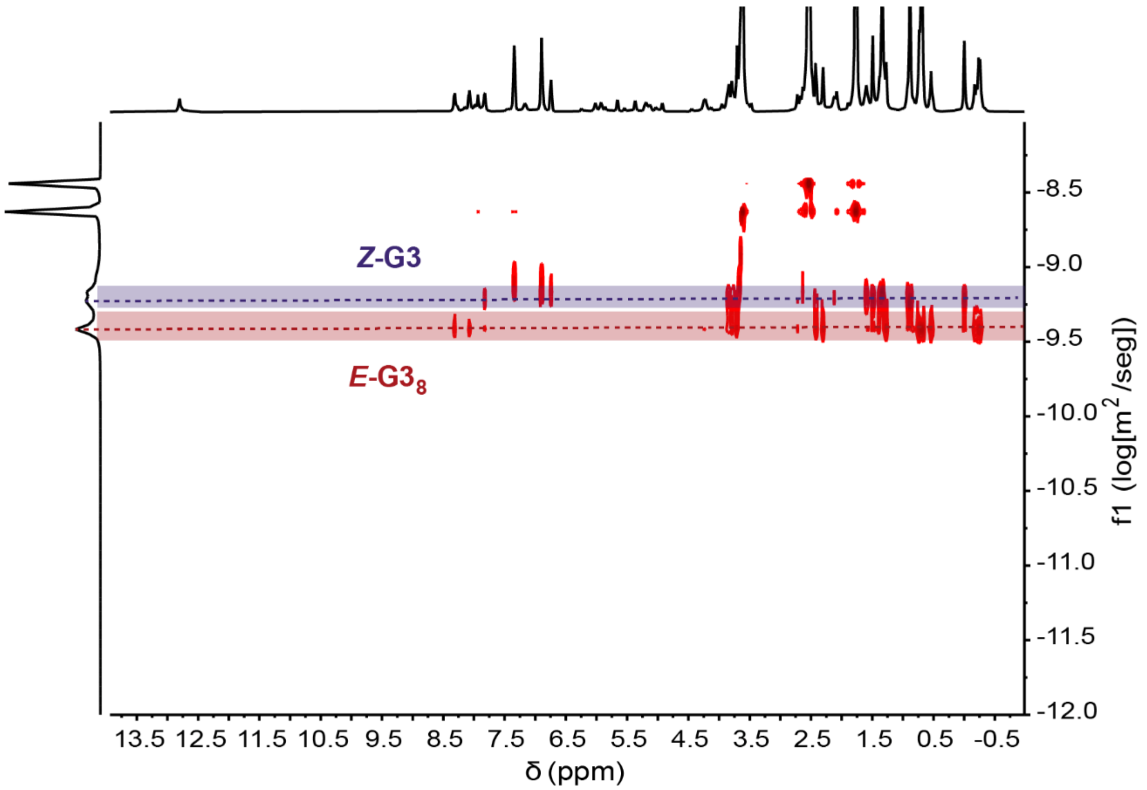

Figure S20. 2D DOSY spectrum of a mixture of the ***E*-G3** and ***Z*-G3** in THF-d_8 with 0.25 eq. of $KBPh_4$ at 298 K, showing different diffusion coefficients.

Table S2. Individual experimental diffusion coefficients (D_i) and the average diffusion coefficients (D_{AV}) obtained from the ^{1}H DOSY NMR spectra at 298 K in THF-d$_8$ for **G3** at different conditions.

	Peak (ppm)	D_i (m^{2}s^{-1})	D_{AV} (m^{2}s^{-1})
***E*-G3**	7.99	$5.99.10^{-10}$	
(Fig. S15)	5.65	$6.02.10^{-10}$	$(5.8\pm0.4).10^{-10}$
	5.25	$5.25.10^{-10}$	
***E*-G3 + K**	12.80	$3.54.10^{-10}$	
(Fig. S16)	8.28	$3.65.10^{-10}$	$(3.7\pm.0.1).10^{-10}$
	6.00	$3.82.10^{-10}$	
***Z*-G3**	7.15	$6.18.10^{-10}$	
(Fig. S17)	5.94	$6.17.10^{-10}$	$(6.18\pm0.02).10^{-10}$
	5.23	$6.20.10^{-10}$	
***Z*-G3 + K**	7.80	$5.67.10^{-10}$	
(Fig. S18)	7.17	$5.57.10^{-10}$	$(5.5\pm0.2).10^{-10}$
	5.95	$5.24.10^{-10}$	
***E*-G3**	7.85	$6.20.10^{-10}$	$(6.19\pm0.02).10^{-10}$
(Fig. S19)	5.95	$6.17.10^{-10}$	
***Z*-G3**	7.15	$6.40.10^{-10}$	$(6.3\pm0.1).10^{-10}$
(Fig. S19)	5.70	$6.19.10^{-10}$	
***E*-G3 + K**	12.82	$4.02.10^{-10}$	$(3.98\pm0.05).10^{-10}$
(Fig. S20)	8.33	$3.95.10^{-10}$	
***Z*-G3 + K**	7.84	$5.67.10^{-10}$	$(6.0\pm0.4).10^{-10}$
(Fig. S20)	7.16	$6.31.10^{-10}$	

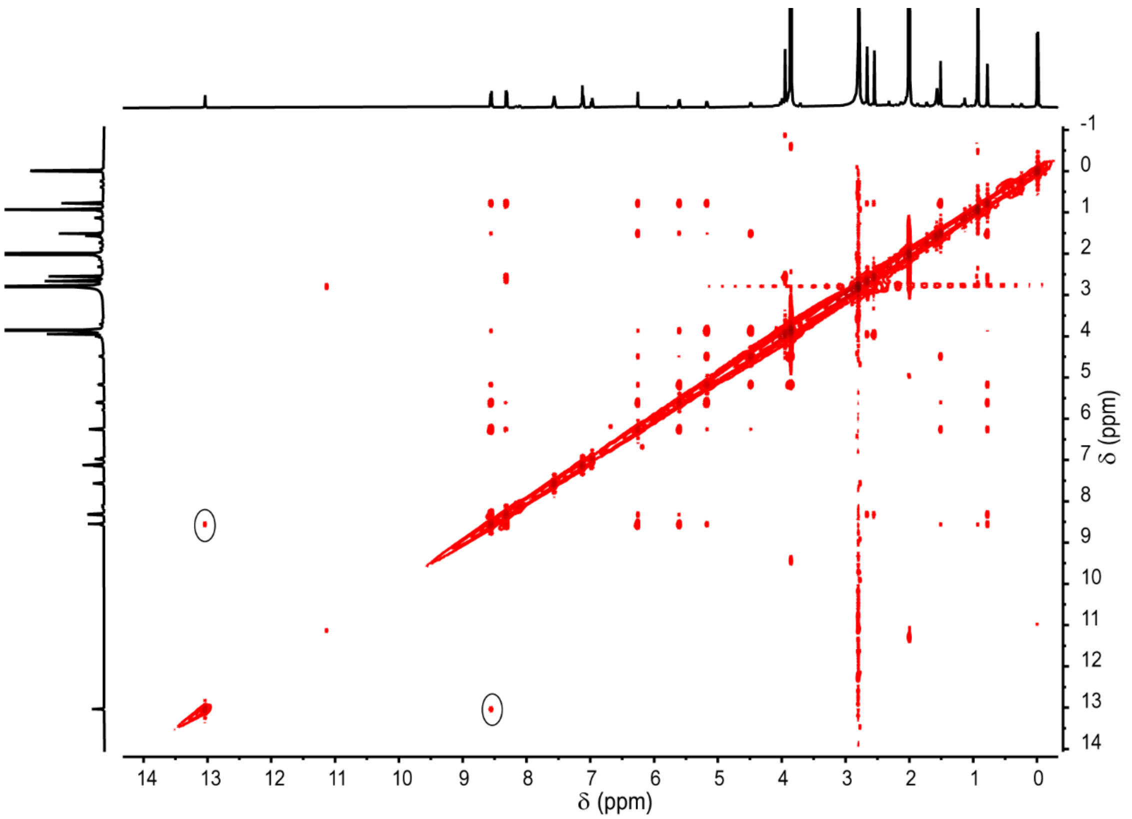

Figure S21. 2D NOESY spectrum of ***E*-G3₈** in THF-d_8 with 0.25 eq. of $KBPh_4$ at 298 K. The signals highlighted denote the contact between the N-H of the amide and one of the protons of the *para* substituted system in the AAP as explained in the main text.

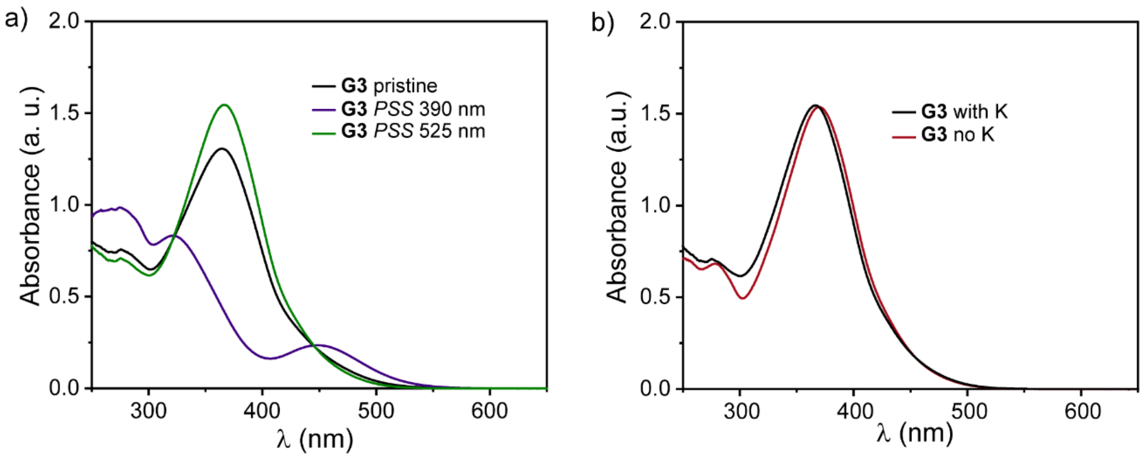

Figure S22. (a) UV-Vis spectra of **G3** in THF without the presence of potassium salts, showing the pristine solution (black lines), after irradiation with 390 nm (purple lines) and after irradiation with 525 nm (green light) and (b) UV Vis spectra of **G3** in the presence of potassium ions (0. 25 eq. of KPF_6, black lines) and without potassium salts (red lines). [**G3**] = 5.10^{-4} M, (l = 1 mm).

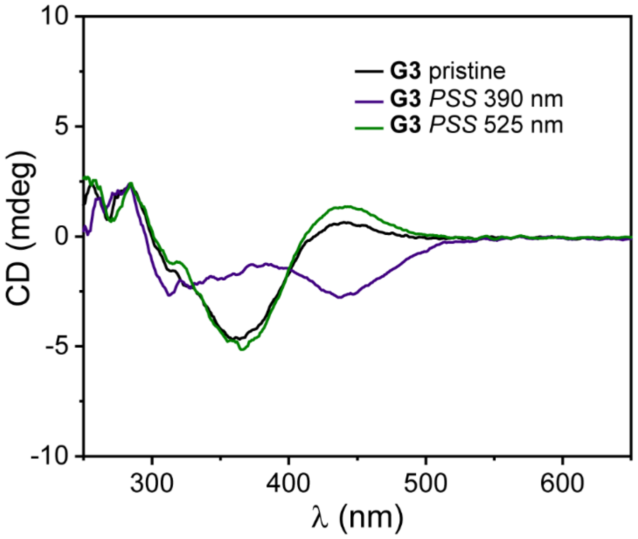

Figure S23. CD spectra of **G3** in THF without the presence of potassium salts, showing the pristine solution (black lines), after irradiation with 390 nm (purple lines) and after irradiation with 525 nm (green light). [**G3**] = 5.10^{-4} M, (l = 1 mm).

2. Computational Details

All geometry optimizations of the monomeric species were performed without symmetry or geometrical constraints using the Gaussian 16 quantum chemistry package, Revision B.01.[7] The PBE0[8] hybrid density functional was used together with the Grimme D3(BJ) empirical dispersion correction,[9, 10] in conjunction with the def2-SVP[11] basis set for all atoms. DFT optimization of the G-octamers was computationally prohibitive due to the size of the system (713 atoms). Therefore, Geometry optimizations of the octameric assemblies were performed using the Conformer-Rotamer Ensemble Sampling Tool (CREST), version 3.0.2,[12-14] at the semiempirical GFN2-xTB[15] level of theory. Solvent effects were included through the analytical linearized Poisson-Boltzmann (ALPB)[16] solvation model, using tetrahydrofuran as solvent. Single-point energy refinements were subsequently performed on the optimized geometries, obtained either at the DFT level for the monomers or at the semiempirical level for the octameric assemblies. These calculations were carried out at the PBE0-D3(BJ)/def2-TZVPP level of theory. Solvent effects were accounted for using the polarizable continuum model (PCM),[17-19] with tetrahydrofuran (THF) as solvent. Therefore, the level of theory is denoted as PCM(THF)-PBE0-D3(BJ)/def2-TZVPP//PBE0-D3(BJ)/def2-SVP for monomers and PCM(THF)-PBE0-D3(BJ)/def2-TZVPP//ALPB(THF)-GFN2-xTB for octamers. UV-Vis absorption and electronic circular dichroism (ECD) spectra were computed using time-dependent density functional theory (TD-DFT)[20] at the PCM(THF)-ωB97X-D[21]/def2-SVP level of theory. Figures of the optimized molecular geometries were generated using CYLview, version 1.0.[22]

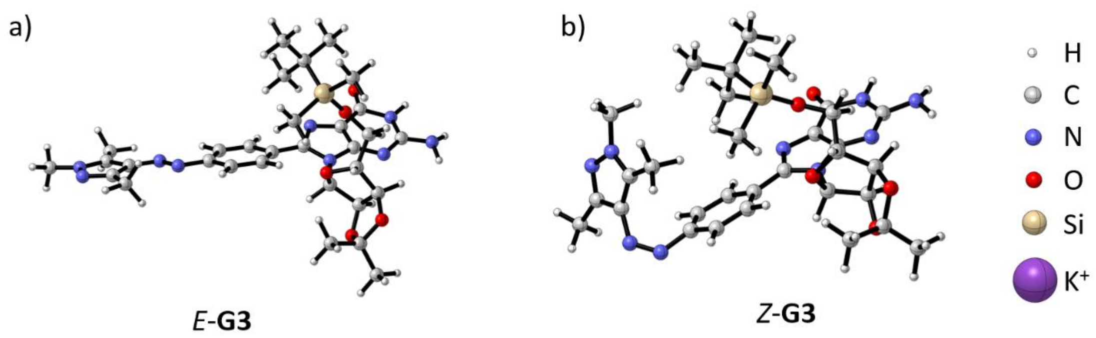

Figure S24. DFT calculated structures of (a) ***E*-G3** and (b) ***Z*-G3**. Both structures were computed at the PBE0-D3BJ/def2-SVP level of theory.

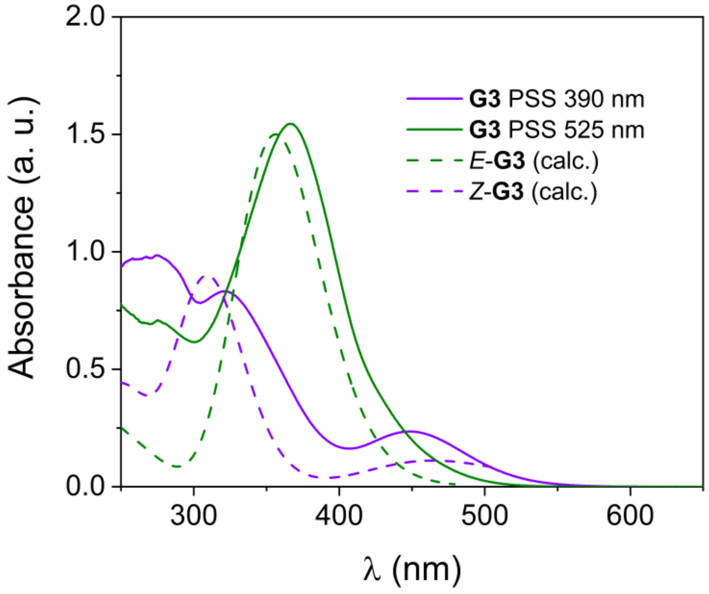

Figure S25. Comparison between experimental UV-VIS spectra of **G3** PSS 390 nm, and **G3** PSS 525 nm (solid lines), and TD-DFT-calculated UV-Vis spectra for the *E*-**G3** and *Z-G3* monomers (dashed lines). Calculated spectra were obtained from the optimized monomer geometries and vertically scaled to facilitate comparison with the experimental profiles.

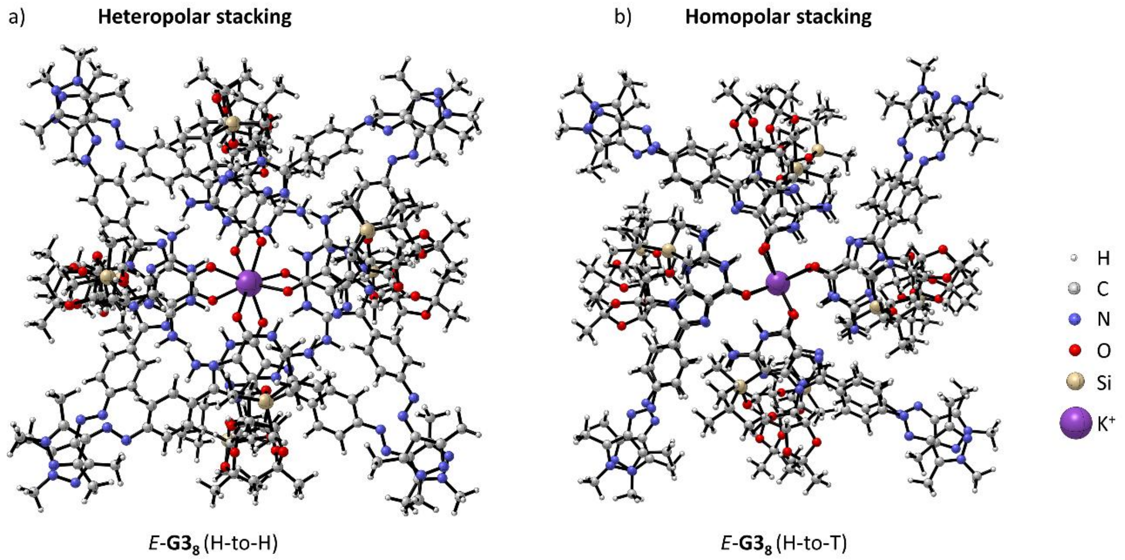

Figure S26. Optimized structures of the two possible arrangements evaluated for the experimentally observed *E*-**G3**$_8$ octamer, namely the head-to-head (H-to-H) and head-to-tail (H-to-T) arrangements. Both assemblies were optimized at the ALPB(THF)-GFN2-xTB-level of theory and consist of two stacked G-quartets surrounding a centrally coordinated K^+ cation.

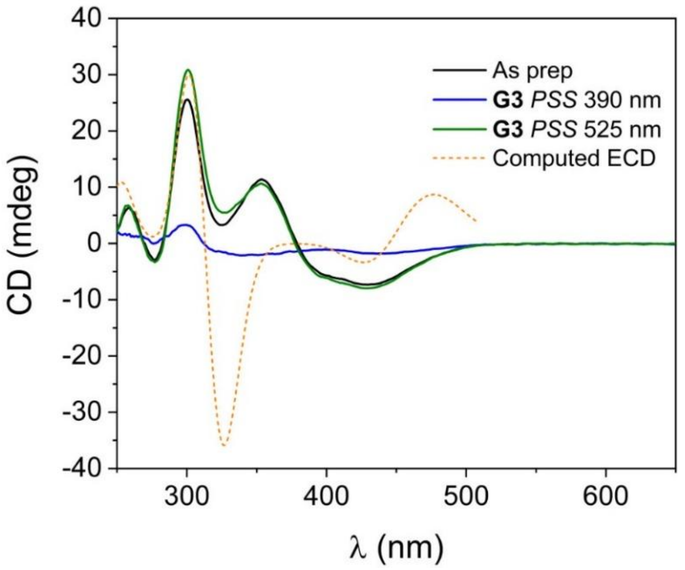

Figure S27. Comparison of the experimental CD spectra of **G3** as prepared (black line), **G3** at the photostationary state upon irradiation at 390 nm (*PSS* 390 nm, blue line), and **G3** at the photostationary state upon irradiation at 525 nm (*PSS* 525 nm, green line), with the TD-DFT-calculated ECD spectrum of the H-to-H octameric arrangement of *E*-$\mathbf{G3_8}$. The calculated spectrum was obtained from the octamer geometry optimized at the ALPB(THF)-GFN2-xTB-level of theory and vertically scaled to facilitate comparison with the experimental spectral profiles.

Although we have attempted to further understand the Cotton effect observed in the AAP region, the calculations have not enabled to fully correlate the molecular arrangement proposed and the complex Cotton effect which emerges in that region after the formation of the G-quadruplex.

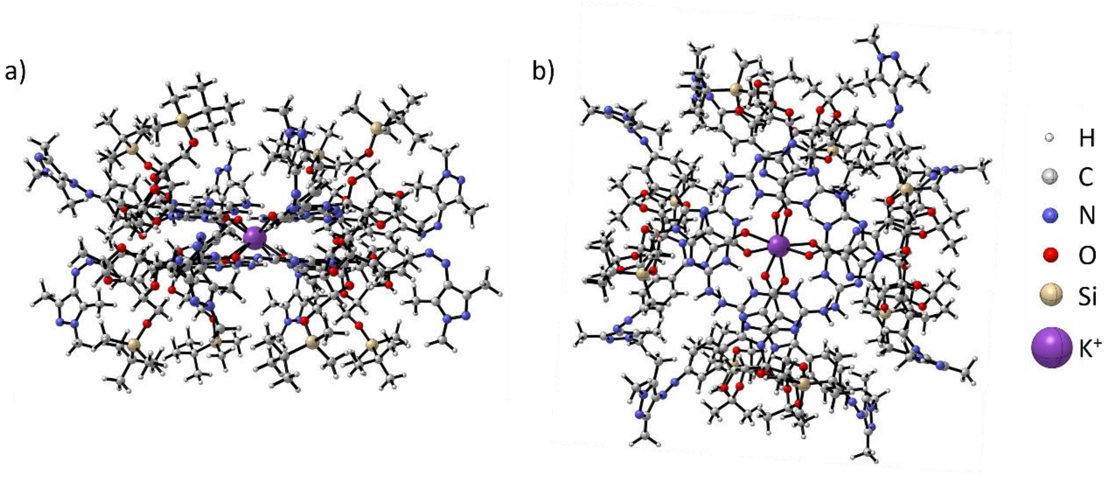

Z-$\mathbf{G3_8}$ (side view)　*Z*-$\mathbf{G3_8}$ (top view)

Figure S28. Side (a) top view (b) of the H-to-H arrangement of ***Z*-$\mathbf{G3_8}$** optimized at the ALPB(THF)-GFN2-xTB-level of theory. The assemblies consist of two stacked G-quartets surrounding a centrally coordinated K^+ cation.

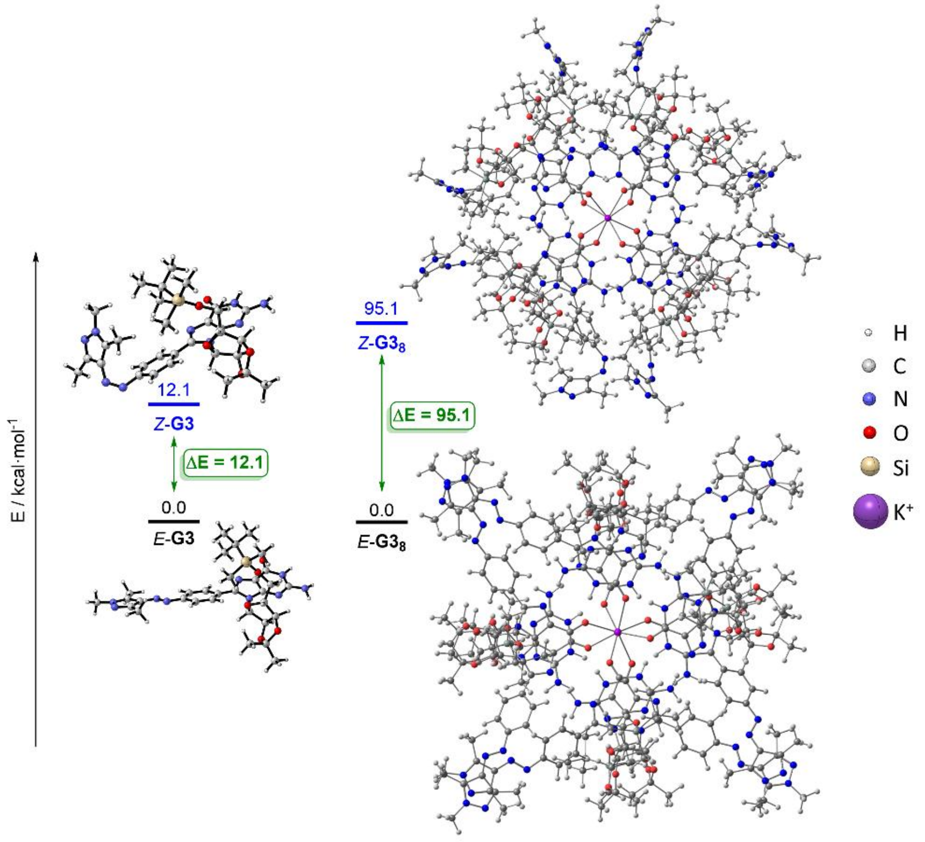

Figure S29. Relative electronic energies between optimized monomeric species *E*-**G3** and *Z*-**G3** and between the H-to-H octameric arrangements of *E*-$\mathbf{G3_8}$ and *Z*-$\mathbf{G3_8}$. The energy data have been computed at the PCM(THF)-PBE0-D3(BJ)/def2-TZVPP//PBE0-D3(BJ)/def2-SVP for monomers and the PCM(THF)-PBE0-D3(BJ)/def2-TZVPP//ALPB(THF)-GFN2-xTB for octamers.

Cartesian coordinates and electronic energies (hartress) of the computed structures

89

E-**G3**

C	-0.742021	-0.563536	-1.549881
N	-1.884698	0.217764	-1.336656
C	-2.926010	-0.476073	-1.902736
C	-2.358801	-1.627974	-2.443293
N	-1.018635	-1.657068	-2.218500
C	-3.204509	-2.583893	-3.113520
O	-2.945747	-3.637478	-3.645009
N	-4.555203	-2.123695	-3.092006
H	-5.206066	-2.777884	-3.514483
C	-5.009324	-0.965900	-2.536708
N	-4.226466	-0.106819	-1.936703
N	-6.349704	-0.731789	-2.583656
H	-6.910835	-1.178209	-3.295267
H	-6.632146	0.199697	-2.309864
C	0.624052	-0.234696	-1.130394
C	1.671033	-0.774427	-1.905445
C	0.950922	0.510582	0.013070
C	2.994133	-0.560017	-1.567613
C	2.282339	0.726868	0.349844
C	3.316489	0.203840	-0.432445
H	1.406065	-1.375896	-2.776741
H	0.166969	0.883511	0.670674
H	3.805994	-0.973015	-2.167203
H	2.550751	1.297262	1.241517
N	4.622797	0.482593	-0.000184
N	5.534095	0.015011	-0.724082
C	7.363369	0.985992	0.800290
N	8.681527	0.948541	0.752959
C	6.819570	0.272523	-0.318808
C	7.930265	-0.191136	-1.032517
N	9.005906	0.237192	-0.344655
C	7.985329	-0.986127	-2.281766
H	6.958573	-1.183413	-2.614715
H	8.517519	-0.451690	-3.084943
H	8.493109	-1.952395	-2.129779
C	6.649298	1.686620	1.898528
H	7.379868	2.125270	2.591421
H	5.995024	2.475429	1.499362
H	5.990899	0.993336	2.442216
C	10.390599	0.067728	-0.691843
H	10.576698	-0.956226	-1.044841
H	10.700059	0.777307	-1.476344
H	10.982819	0.254964	0.211130
O	-1.865889	1.341049	0.747673
C	-3.184000	1.326529	1.270382
H	-3.119988	1.762466	2.278522
C	-3.995717	2.251514	0.350925
H	-4.992352	1.837213	0.139942
C	-3.118001	2.392794	-0.915847
H	-3.634911	2.138809	-1.847736
C	-1.901555	1.494453	-0.641499
O	-2.741194	3.741899	-0.931419
O	-4.145636	3.559759	0.823760
C	-3.153165	4.395318	0.259253
C	-1.973729	4.564231	1.205469
H	-2.305403	5.032954	2.142812
H	-1.522829	3.588532	1.430763
H	-1.209042	5.202709	0.740749
C	-3.796144	5.713998	-0.112554
H	-4.636144	5.526587	-0.794654
H	-4.169869	6.221249	0.787561
H	-3.065432	6.365035	-0.611539
H	-0.989455	2.026850	-0.941492
C	-3.763941	-0.095155	1.327043
H	-4.557494	-0.099074	2.101279
H	-4.259795	-0.319275	0.370161
O	-2.804199	-1.070577	1.543810
Si	-2.023320	-1.516481	2.967382
C	-3.320143	-1.701860	4.317152
H	-3.785429	-0.732401	4.558060
H	-4.119362	-2.397123	4.017696
H	-2.867190	-2.083746	5.245660
C	-0.770846	-0.224732	3.501430
H	-0.297144	-0.519266	4.451429
H	0.019734	-0.100747	2.747642
H	-1.246636	0.755888	3.655743
C	-1.191922	-3.161300	2.521571
C	-2.270555	-4.162864	2.098380
H	-2.853173	-3.781736	1.245734
H	-1.808747	-5.117448	1.790136
H	-2.972154	-4.386781	2.918540
C	-0.224236	-2.935545	1.355313
H	0.264868	-3.885419	1.076165
H	-0.741901	-2.558519	0.461229
H	0.574157	-2.219472	1.606727
C	-0.428177	-3.696549	3.736197
H	0.053049	-4.660067	3.491726
H	0.370091	-3.008064	4.057393
H	-1.089882	-3.873020	4.600267

Electronic energy = -2364.080307

89

Z-**G3**

C	-0.405258	-0.038486	-1.637074
N	-1.752822	0.203612	-1.339797
C	-2.444667	-0.892908	-1.794399
C	-1.487641	-1.730428	-2.360747
N	-0.248409	-1.185276	-2.254892
C	-1.895502	-2.994511	-2.920091
O	-1.252515	-3.876856	-3.437503
N	-3.308488	-3.140890	-2.788127
H	-3.651366	-4.032131	-3.132029
C	-4.166172	-2.249734	-2.217452
N	-3.776981	-1.108165	-1.710629
N	-5.478978	-2.602228	-2.144312
H	-5.850461	-3.284136	-2.790570
H	-6.107424	-1.860196	-1.866889
C	0.739154	0.840346	-1.361897
C	1.902546	0.591363	-2.119250
C	0.790482	1.861409	-0.398878
C	3.068293	1.304947	-1.909457
C	1.949960	2.609215	-0.217641
C	3.110759	2.318928	-0.937516
H	1.863900	-0.198362	-2.870924
H	-0.054150	2.055550	0.258143
H	3.956320	1.087263	-2.505242
H	1.977368	3.412967	0.521207
N	4.237857	3.155651	-0.774504
N	5.399102	2.720186	-0.660454
C	6.815251	0.711847	-1.007091
N	6.891524	-0.528487	-0.556759
C	5.691679	1.374641	-0.437551
C	5.124070	0.446303	0.442253
N	5.882356	-0.667043	0.315275
C	4.016222	0.566825	1.420224
H	3.884612	1.614736	1.716902
H	3.059402	0.230757	0.990521

H	4.212138	-0.023677	2.326950
C	7.760920	1.262927	-2.013024
H	8.681130	0.664713	-2.034246
H	7.324468	1.254714	-3.024967
H	8.002995	2.308626	-1.772793
C	5.737630	-1.900366	1.043784
H	6.060832	-1.791828	2.091709
H	4.693501	-2.244223	1.024017
H	6.374218	-2.642594	0.549804
O	-1.940960	1.262414	0.756767
C	-3.020224	0.655770	1.449082
H	-3.004661	1.072530	2.467288
C	-4.285336	1.115361	0.708341
H	-5.019198	0.300634	0.620655
C	-3.760682	1.609133	-0.661707
H	-4.242852	1.134597	-1.522823
C	-2.245870	1.355479	-0.605444
O	-4.024363	2.984910	-0.661143
O	-4.918408	2.229736	1.267560
C	-4.503253	3.409643	0.605528
C	-3.399181	4.112454	1.380957
H	-3.764537	4.407874	2.374679
H	-2.534681	3.445794	1.499821
H	-3.075366	5.012886	0.839969
C	-5.716559	4.288839	0.392176
H	-6.474133	3.728627	-0.171931
H	-6.140075	4.592652	1.359373
H	-5.438548	5.188307	-0.173890
H	-1.728745	2.235902	-1.007812
C	-2.905691	-0.877450	1.483780
H	-3.479778	-1.229885	2.364036
H	-3.407619	-1.295220	0.597559
O	-1.600140	-1.342287	1.483554
Si	-0.462367	-1.371545	2.723441
C	-1.334947	-1.837134	4.322412
H	-2.019217	-1.037917	4.649841
H	-1.920162	-2.763006	4.212394
H	-0.605927	-1.992245	5.133566
C	0.357600	0.300925	2.933840
H	1.091678	0.275098	3.754797
H	0.873568	0.608252	2.013181
H	-0.385790	1.077537	3.169401
C	0.779497	-2.695236	2.163933
C	0.084860	-4.060033	2.160229
H	-0.805429	-4.050345	1.512279
H	0.767484	-4.838758	1.776492
H	-0.230615	-4.367730	3.170286
C	1.256301	-2.369720	0.745673
H	2.006803	-3.109398	0.413146
H	0.433354	-2.385682	0.018037
H	1.723393	-1.374928	0.677624
C	1.972802	-2.719879	3.123212
H	2.688530	-3.507069	2.824784
H	2.519014	-1.763059	3.123557
H	1.671881	-2.933665	4.162224

Electronic energy = -2364.061083

713

E-**G3$_{8}$** (H-to-H)

O	1.089669	2.117480	-0.752127
N	3.245649	1.474315	-1.029376
C	4.577067	1.716106	-1.198815
N	5.083532	2.936145	-1.270123
C	4.183235	3.926135	-1.187894
C	2.806970	3.755719	-1.010149
C	2.271361	2.439207	-0.908754
N	4.401472	5.276069	-1.265556
C	3.150302	5.866525	-1.159684
N	2.196620	4.983287	-1.004385
H	5.083834	-0.298338	-1.223671
H	6.366318	0.875264	-1.468042
H	2.908396	0.505053	-0.963845
N	0.518121	-5.253592	-1.578596
O	1.967063	-0.979720	-0.852946
N	1.321898	-3.126025	-1.193912
C	1.562123	-4.452632	-1.396314
N	2.781038	-4.960605	-1.470176
C	3.773983	-4.065505	-1.354763
C	3.602775	-2.691142	-1.160316
C	2.287276	-2.156483	-1.046694
N	5.124483	-4.283011	-1.406894
C	5.714767	-3.035259	-1.275920
N	4.829788	-2.082179	-1.125942
H	-0.448384	-4.955765	-1.401456
H	0.709545	-6.235839	-1.680688
H	0.353174	-2.792667	-1.105221
N	-5.425599	-0.403087	-1.359620
O	-1.124043	-1.872598	-0.884090
N	-3.281426	-1.217186	-1.105317
C	-4.617260	-1.449724	-1.241643
N	-5.132276	-2.665788	-1.310158
C	-4.236320	-3.662664	-1.260840
C	-2.852785	-3.500534	-1.135662
C	-2.309391	-2.188239	-1.023440
N	-4.463246	-5.009940	-1.333142
C	-3.212489	-5.607532	-1.286342
N	-2.248046	-4.730515	-1.165694
H	-5.117961	0.563738	-1.199175
H	-6.409573	-0.595016	-1.441257
H	-2.939982	-0.251369	-1.016215
N	-0.561024	5.531590	-1.288087
O	-2.011235	1.224466	-0.811580
N	-1.364309	3.384993	-1.023505
C	-1.602803	4.721858	-1.141147
N	-2.820793	5.236295	-1.164869
C	-3.815592	4.339398	-1.092359
C	-3.647568	2.954564	-0.993040
C	-2.332033	2.410913	-0.927085
N	-5.165090	4.565541	-1.112313
C	-5.759780	3.313735	-1.060522
N	-4.877047	2.349304	-0.986064
H	0.410549	5.223255	-1.158327
H	-0.754343	6.516725	-1.350656
H	-0.396413	3.044677	-0.957153
N	5.389291	0.676617	-1.336711
N	-5.336065	1.235695	2.176413
O	-0.872542	2.144701	1.831814
N	-3.096972	1.769279	2.039306
C	-4.396096	2.170710	2.138143
N	-4.749026	3.442915	2.217921
C	-3.734640	4.319633	2.152192
C	-2.386558	3.981998	2.011891
C	-2.010637	2.608968	1.949549
C	-2.482976	6.130148	2.018879
N	-1.641138	5.124537	1.926696
H	-5.131274	0.236009	2.066747
H	-6.293254	1.534679	2.239596
H	-2.877634	0.767974	1.957972
N	-1.181158	-5.256541	2.091269
O	-2.097277	-0.792763	1.745264
N	-1.718428	-3.018594	1.937728
C	-2.117448	-4.319320	2.027273

N	-3.389673	-4.676528	2.078891
C	-4.267241	-3.663703	2.011502
C	-3.932047	-2.312768	1.894832
C	-2.559564	-1.933534	1.848565
C	-6.079335	-2.414751	1.886884
N	-5.075958	-1.568432	1.814888
H	-0.182725	-5.052397	1.970965
H	-1.481071	-6.213643	2.153624
H	-0.716156	-2.795916	1.879264
N	5.314806	-1.139005	2.117386
O	0.848148	-2.016753	1.731048
N	3.074248	-1.658384	1.953825
C	4.372518	-2.068381	2.028290
N	4.722384	-3.344305	2.032971
C	3.706398	-4.214215	1.916616
C	2.358573	-3.864096	1.804859
C	1.985932	-2.489084	1.820422
N	3.761218	-5.580310	1.841803
C	2.446109	-6.008927	1.675059
N	1.607985	-4.995795	1.654647
H	5.111480	-0.135259	2.057492
H	6.270507	-1.443006	2.178870
H	2.855383	-0.653956	1.936351
N	1.160949	5.377464	2.249133
O	2.064188	0.917166	1.837289
N	1.693472	3.140478	2.075255
C	2.096146	4.439255	2.174896
N	3.369210	4.794701	2.223303
C	4.240768	3.778543	2.131054
C	3.903873	2.429987	1.995339
C	2.530244	2.053980	1.957134
N	5.607768	3.841206	2.112818
C	6.049505	2.530821	1.962508
N	5.046168	1.685843	1.887606
H	0.159392	5.173828	2.152814
H	1.461727	6.334466	2.305202
H	0.690782	2.921243	2.013250
K	-0.018055	0.085936	0.513824
C	2.882307	7.297099	-1.243788
C	3.587255	8.244574	-0.499057
C	1.843976	7.729163	-2.073856
C	3.255311	9.579823	-0.569052
C	1.509704	9.061770	-2.148021
C	2.195313	10.009740	-1.374556
H	4.383449	7.937328	0.157972
H	1.316357	7.002357	-2.670032
H	3.787935	10.308437	0.021544
H	0.717422	9.403196	-2.796268
C	-7.192236	3.054668	-1.143672
C	-7.626647	2.059072	-2.023950
C	-8.139050	3.722760	-0.365425
C	-8.958828	1.726931	-2.110901
C	-9.474609	3.394806	-0.449672
C	-9.905155	2.372997	-1.302242
H	-6.901152	1.563162	-2.648454
H	-7.835237	4.487607	0.328529
H	-9.301189	0.965419	-2.794669
H	-10.202157	3.898435	0.166889
C	-2.965374	-7.036788	-1.436862
C	-1.987592	-7.439588	-2.350951
C	-3.644512	-8.011420	-0.704341
C	-1.695940	-8.772266	-2.529398
C	-3.354535	-9.347056	-0.878163
C	-2.363139	-9.751079	-1.778816
H	-1.475973	-6.688368	-2.930744
H	-4.389762	-7.729691	0.020202
H	-0.953385	-9.089762	-3.245078

H	-3.871540	-10.097863	-0.301846
C	7.145815	-2.764719	-1.349239
C	8.093430	-3.467142	-0.602910
C	7.576362	-1.723257	-2.176256
C	9.427448	-3.128976	-0.667328
C	8.907283	-1.380842	-2.243244
C	9.854702	-2.062815	-1.466015
H	7.789639	-4.267769	0.049560
H	6.850326	-1.199054	-2.776638
H	10.156589	-3.658204	-0.074573
H	9.246554	-0.584223	-2.887514
C	-7.450935	-1.955233	1.781034
C	-8.558296	-2.577060	2.366464
C	-7.662900	-0.760297	1.073741
C	-9.825143	-2.057898	2.208287
C	-8.917178	-0.229070	0.930355
C	-10.035812	-0.882060	1.471411
H	-8.430055	-3.452714	2.978647
H	-6.815515	-0.258163	0.642569
H	-10.673215	-2.536624	2.674596
H	-9.067562	0.683914	0.378340
C	-2.021324	7.500381	1.898783
C	-2.630762	8.615865	2.481908
C	-0.838003	7.703353	1.169117
C	-2.114080	9.880202	2.297025
C	-0.308665	8.955247	0.998959
C	-0.952490	10.081373	1.535350
H	-3.494166	8.497349	3.112918
H	-0.343482	6.850420	0.740438
H	-2.584369	10.734398	2.760648
H	0.594973	9.097224	0.428907
C	7.423448	2.086295	1.834133
C	7.646154	0.923505	1.079420
C	8.522518	2.699943	2.443340
C	8.907559	0.417091	0.912087
C	9.796088	2.205261	2.262091
C	10.018934	1.062516	1.477472
H	6.803258	0.428648	0.630817
H	8.380454	3.546542	3.094009
H	9.069204	-0.471324	0.324362
H	10.638016	2.676126	2.746118
C	1.972095	-7.364751	1.470029
C	0.758996	-7.508707	0.774748
C	2.595972	-8.525150	1.939764
C	0.215366	-8.742162	0.530702
C	2.065553	-9.769589	1.678076
C	0.873782	-9.908648	0.950368
H	0.251060	-6.625012	0.432331
H	3.483250	-8.461543	2.543731
H	-0.711886	-8.835994	-0.009967
H	2.548888	-10.658696	2.054473
N	-0.306286	11.275718	1.435117
N	-0.942241	12.364764	1.278371
C	1.190212	13.733829	1.486035
N	1.440717	15.019353	1.380379
C	-0.212522	13.504949	1.310312
C	-0.757721	14.784363	1.101287
N	0.266836	15.638701	1.155982
C	-2.165021	15.196639	0.908651
H	-2.763200	14.317424	0.689260
H	-2.547506	15.656376	1.820528
H	-2.245752	15.922909	0.103678
C	2.249477	12.724540	1.700765
H	3.202591	13.217637	1.870647
H	1.997631	12.086961	2.545997
H	2.313851	12.090511	0.814802
C	0.239985	17.057091	0.943291

H	0.102344	17.269863	-0.118681
H	-0.565042	17.510197	1.519926
H	1.199102	17.455668	1.263786
N	11.217202	0.424874	1.380248
N	12.310252	1.060807	1.258516
C	13.657554	-1.089139	1.414272
N	14.943323	-1.347155	1.332006
C	13.445165	0.322045	1.290780
C	14.734017	0.863430	1.140227
N	15.578010	-0.170666	1.175380
C	15.166009	2.272680	1.018293
H	14.298398	2.892509	0.813534
H	15.617833	2.607788	1.952461
H	15.905658	2.380597	0.228472
C	12.635614	-2.148276	1.561759
H	13.119991	-3.111401	1.697034
H	11.985700	-1.930482	2.407113
H	12.015039	-2.169815	0.664050
C	17.003958	-0.144036	1.023750
H	17.265368	0.095012	-0.008527
H	17.441650	0.593859	1.695220
H	17.377471	-1.135526	1.266288
N	0.222755	-11.093260	0.789272
N	0.851082	-12.163927	0.518421
C	-1.252338	-13.567383	0.781417
N	-1.501904	-14.845338	0.605094
C	0.130485	-13.310103	0.514285
C	0.665769	-14.564830	0.172005
N	-0.345966	-15.433085	0.244509
C	2.056733	-14.942075	-0.160961
H	2.627680	-14.040084	-0.359769
H	2.514417	-15.462810	0.680843
H	2.079575	-15.604707	-1.022601
C	-2.298500	-12.588034	1.144784
H	-3.228919	-13.103908	1.365061
H	-1.982024	-11.994718	2.000388
H	-2.442656	-11.907289	0.304162
C	-0.333243	-16.831266	-0.076386
H	-0.320936	-16.961351	-1.160485
H	0.536956	-17.312597	0.366710
H	-1.245811	-17.267461	0.322022
N	11.204453	-1.773681	-1.491643
N	11.501762	-0.566188	-1.790754
C	14.028556	-0.949258	-2.001803
N	15.016132	-0.115406	-2.244834
C	12.794935	-0.219594	-1.943568
C	13.162485	1.120356	-2.173716
N	14.485647	1.121185	-2.340464
C	12.319297	2.332112	-2.215444
H	11.287518	2.043674	-2.039675
H	12.627339	3.034487	-1.442184
H	12.399458	2.826949	-3.182306
C	14.239178	-2.406547	-1.845015
H	15.125796	-2.712659	-2.393887
H	14.386625	-2.642797	-0.791367
H	13.363011	-2.946376	-2.195863
C	15.322090	2.237627	-2.677335
H	15.130545	2.556694	-3.703643
H	15.125435	3.071829	-2.005277
H	16.356744	1.914593	-2.593488
N	-11.229618	-0.231851	1.404378
N	-12.324170	-0.859394	1.253984
C	-13.672918	1.276319	1.539512
N	-14.958425	1.538796	1.465601
C	-13.458802	-0.123316	1.325478
C	-14.746073	-0.654031	1.129926
N	-15.591121	0.375214	1.225805
C	-15.174139	-2.052682	0.911662
H	-15.908791	-2.108187	0.112072
H	-14.303388	-2.654798	0.670498
H	-15.629229	-2.450244	1.819436
C	-12.651929	2.323441	1.759441
H	-13.136565	3.274489	1.962302
H	-11.998997	2.047689	2.585366
H	-12.034546	2.406510	0.863136
C	-17.014086	0.361542	1.045919
H	-17.253587	0.212618	-0.008554
H	-17.463409	-0.431512	1.642279
H	-17.394248	1.328636	1.364680
N	1.920390	11.362075	-1.411041
N	0.728900	11.671043	-1.760177
C	1.148830	14.193864	-1.944181
N	0.339471	15.191277	-2.226585
C	0.404062	12.967726	-1.930391
C	-0.917010	13.350057	-2.235459
N	-0.895076	14.674038	-2.394701
C	-2.131546	12.519755	-2.365953
H	-1.870454	11.486085	-2.161310
H	-2.892139	12.841737	-1.656177
H	-2.545238	12.597382	-3.370685
C	2.598865	14.387348	-1.715399
H	2.943041	15.266107	-2.254390
H	2.785554	14.538637	-0.652237
H	3.142997	13.501925	-2.034629
C	-1.980481	15.523956	-2.793862
H	-2.227443	15.352881	-3.843413
H	-2.861377	15.321509	-2.186075
H	-1.658861	16.555001	-2.669324
N	-11.255948	2.088692	-1.342952
N	-11.552601	0.901558	-1.715860
C	-14.082654	1.286418	-1.885916
N	-15.068104	0.465935	-2.178658
C	-12.845205	0.560982	-1.885807
C	-13.207930	-0.761833	-2.206003
N	-14.532232	-0.758265	-2.363349
C	-12.359031	-1.962180	-2.347618
H	-11.327421	-1.683956	-2.154740
H	-12.659316	-2.727360	-1.633117
H	-12.442762	-2.377086	-3.351340
C	-14.297938	2.729369	-1.632601
H	-13.422444	3.292682	-1.945787
H	-15.184584	3.069002	-2.161378
H	-14.447323	2.895836	-0.565920
C	-15.364541	-1.853751	-2.772065
H	-15.162776	-2.112303	-3.813249
H	-15.174166	-2.726236	-2.148243
H	-16.400203	-1.536585	-2.678942
N	-2.138030	-11.106140	-1.921532
N	-0.995481	-11.425432	-2.401319
C	-1.512462	-13.922301	-2.675517
N	-0.768851	-14.923698	-3.093168
C	-0.731698	-12.718783	-2.675380
C	0.537857	-13.117939	-3.136919
N	0.457613	-14.430346	-3.362351
C	1.758220	-12.314641	-3.353723
H	1.550583	-11.284341	-3.082397
H	2.575131	-12.687439	-2.737703
H	2.068672	-12.359819	-4.396976
C	-2.935372	-14.090553	-2.302464
H	-3.479000	-13.170989	-2.503562
H	-3.366201	-14.922300	-2.853529
H	-3.010620	-14.306616	-1.236727
C	1.470433	-15.285187	-3.913326
H	1.634605	-15.046388	-4.965773

H	2.407985	-15.157968	-3.373148
H	1.119996	-16.311213	-3.832368
O	6.452453	-5.240278	-3.079988
C	5.630905	-5.783911	-4.105050
H	6.295432	-6.154511	-4.889362
C	4.841783	-6.940741	-3.467795
H	3.809231	-6.940694	-3.820357
O	5.386261	-8.212080	-3.707613
C	6.181588	-8.597919	-2.593211
C	7.657364	-8.248940	-2.808189
H	8.019361	-8.765037	-3.692210
H	7.785248	-7.178442	-2.940985
H	8.227846	-8.568626	-1.941546
C	6.000021	-10.096411	-2.366139
H	4.947674	-10.309691	-2.209303
H	6.350104	-10.631041	-3.242554
H	6.565032	-10.404563	-1.493104
C	4.696388	-4.727633	-4.708124
H	4.018860	-4.351107	-3.933346
H	5.307063	-3.895243	-5.075074
O	3.913696	-5.254252	-5.724059
Si	4.390572	-5.762852	-7.220443
C	5.343025	-7.419082	-7.157542
H	6.417668	-7.261765	-7.129441
H	5.073051	-7.992326	-6.275009
H	5.138358	-8.033952	-8.027639
C	5.496918	-4.448005	-8.053870
H	5.781648	-4.733722	-9.060621
H	4.994129	-3.486935	-8.113524
H	6.413226	-4.300970	-7.488202
C	2.688484	-5.961106	-8.093389
C	2.890988	-6.395720	-9.545174
H	3.420540	-7.343122	-9.596481
H	1.929512	-6.517187	-10.043291
H	3.465918	-5.654223	-10.093301
C	1.849657	-7.020935	-7.374916
H	1.691011	-6.731406	-6.339977
C	1.913739	-4.640340	-8.086463
H	0.935629	-4.771081	-8.549603
H	1.766923	-4.289712	-7.068024
H	2.450285	-3.874105	-8.640870
H	0.877899	-7.130162	-7.856615
H	2.350654	-7.985384	-7.391001
O	5.635206	-7.886950	-1.481475
C	4.993430	-6.728811	-1.946398
H	4.048228	-6.612294	-1.416094
C	5.864964	-5.464169	-1.821210
H	6.672302	-5.638180	-1.103834
O	-5.514974	-6.323825	-2.958977
C	-6.097145	-5.493716	-3.955306
H	-6.501327	-6.151711	-4.728213
C	-7.222447	-4.704838	-3.264693
H	-7.242503	-3.674433	-3.623538
O	-8.503120	-5.253833	-3.436365
C	-8.838538	-6.028663	-2.290828
C	-8.509679	-7.509841	-2.498962
H	-9.083725	-7.888471	-3.339107
H	-7.450592	-7.644927	-2.699075
H	-8.771876	-8.059832	-1.600424
C	-10.322056	-5.832111	-1.989724
H	-10.523881	-4.773313	-1.861792
H	-10.906387	-6.214336	-2.819682
H	-10.579649	-6.361686	-1.078973
C	-5.064264	-4.556401	-4.593787
H	-4.659783	-3.880483	-3.831914
H	-4.245862	-5.165197	-4.993519
O	-5.630003	-3.770888	-5.586176

Si	-6.164291	-4.245334	-7.074392
C	-7.798140	-5.232966	-6.979804
H	-7.617136	-6.303796	-6.947955
H	-8.363154	-4.969189	-6.089923
H	-8.430697	-5.047061	-7.841317
C	-4.843380	-5.317056	-7.942887
H	-5.120127	-5.557601	-8.963483
H	-3.881751	-4.812169	-7.971523
H	-4.701681	-6.257473	-7.416625
C	-6.416160	-2.540663	-7.927973
C	-6.885477	-2.739500	-9.369497
H	-7.825986	-3.282826	-9.400287
H	-7.034194	-1.776530	-9.857152
H	-6.150108	-3.299124	-9.941367
C	-7.471029	-1.725652	-7.175358
H	-7.158918	-1.571100	-6.146222
C	-5.108611	-1.743915	-7.949046
H	-5.267100	-0.764955	-8.401443
H	-4.734477	-1.598938	-6.938765
H	-4.348092	-2.263961	-8.526551
H	-7.607809	-0.751853	-7.645692
H	-8.427593	-2.241911	-7.172375
O	-8.071300	-5.470621	-1.224407
C	-6.931855	-4.848120	-1.755369
H	-6.775738	-3.901755	-1.237740
C	-5.672588	-5.735140	-1.690997
H	-5.825448	-6.540714	-0.966799
O	-6.549888	5.632092	-2.667021
C	-5.761735	6.219501	-3.693653
H	-6.450757	6.620353	-4.440612
C	-4.952956	7.346929	-3.030817
H	-3.941653	7.381994	-3.439710
O	-5.523647	8.622972	-3.161682
C	-6.234346	8.944849	-1.971403
C	-7.723309	8.609661	-2.097686
H	-8.159305	9.207517	-2.892083
H	-7.862948	7.556651	-2.324699
H	-8.216404	8.835784	-1.157267
C	-6.027175	10.426962	-1.670547
H	-4.963835	10.631047	-1.594233
H	-6.450798	11.014914	-2.477552
H	-6.511739	10.678068	-0.733362
C	-4.841232	5.193157	-4.366369
H	-4.132847	4.796583	-3.630252
H	-5.457638	4.368415	-4.740608
O	-4.099161	5.763281	-5.389132
Si	-4.633863	6.289644	-6.859481
C	-5.627678	7.917628	-6.732257
H	-6.694751	7.728735	-6.653626
H	-5.330460	8.490641	-5.858021
H	-5.483463	8.545488	-7.605354
C	-5.732363	4.960106	-7.679980
H	-6.023386	5.235219	-8.687841
H	-5.221221	4.002928	-7.733199
H	-6.645678	4.809834	-7.110239
C	-2.965758	6.550300	-7.780295
C	-3.227175	6.983623	-9.223083
H	-3.793686	7.910322	-9.253481
H	-2.286124	7.143756	-9.748134
H	-3.789196	6.223171	-9.758384
C	-2.142047	7.635946	-7.083067
H	-1.945958	7.352856	-6.052579
C	-2.146505	5.256838	-7.803183
H	-1.188142	5.422231	-8.295323
H	-1.957274	4.907970	-6.791256
H	-2.673676	4.474705	-8.343786
H	-1.187899	7.774886	-7.591499

H	-2.672537	8.584627	-7.085789
O	-5.614037	8.171487	-0.944542
C	-5.020033	7.038523	-1.519636
H	-4.049376	6.875315	-1.051561
C	-5.904519	5.778718	-1.425734
H	-6.676571	5.926158	-0.664786
O	5.387228	6.397263	-3.071491
C	5.995967	5.474753	-3.966082
H	6.407859	6.056300	-4.797044
C	7.128775	4.784597	-3.186656
H	7.144641	3.715916	-3.404667
O	8.404034	5.309501	-3.449384
C	8.740506	6.247101	-2.433725
C	8.385904	7.677943	-2.850739
H	8.922278	7.926265	-3.761672
H	7.318334	7.775355	-3.026560
H	8.676228	8.362216	-2.059650
C	10.230455	6.117604	-2.128516
H	10.447174	5.096974	-1.828944
H	10.796166	6.360700	-3.021386
H	10.496014	6.795898	-1.324936
C	4.979600	4.463149	-4.515806
H	4.540592	3.904608	-3.681408
H	4.180620	5.011058	-5.024928
O	5.589938	3.546801	-5.359186
Si	5.589105	3.426530	-7.003491
C	3.880747	3.907906	-7.706886
H	3.102097	3.311998	-7.238601
H	3.646951	4.952729	-7.522416
H	3.816340	3.744222	-8.777003
C	5.915058	1.565558	-7.217748
H	5.872079	1.279272	-8.262148
H	6.890124	1.284335	-6.835335
H	5.172898	0.982670	-6.682059
C	6.982342	4.486998	-7.830455
C	7.236140	3.996761	-9.260359
H	6.333486	4.070101	-9.861317
H	8.010800	4.598168	-9.734094
H	7.562126	2.960531	-9.259256
C	6.597332	5.969260	-7.902562
H	6.368709	6.365611	-6.915375
C	8.288137	4.354701	-7.038940
H	9.072244	4.954977	-7.499787
H	8.162613	4.685369	-6.010739
H	8.619723	3.319505	-7.021489
H	7.418213	6.552665	-8.318699
H	5.725931	6.111114	-8.537122
O	7.994389	5.838475	-1.287891
C	6.860053	5.128864	-1.706280
H	6.734603	4.257736	-1.063866
C	5.578933	5.984303	-1.739006
H	5.711045	6.882509	-1.129999
O	6.954485	5.143918	3.547202
C	6.232924	6.137230	4.270017
H	6.957047	6.637200	4.920103
C	5.683116	7.108136	3.213928
H	4.681152	7.456454	3.470682
O	6.497161	8.233883	2.997367
C	7.229285	8.066364	1.786438
C	8.635831	7.518672	2.039971
H	9.256387	8.294645	2.476525
H	8.600431	6.671761	2.718933
H	9.050752	7.195117	1.089499
C	7.280766	9.400396	1.049143
H	6.271244	9.741651	0.842083
H	7.815067	9.268787	0.114654
C	5.137802	5.529933	5.154376
H	4.398270	6.301367	5.389873
H	4.642308	4.719761	4.608394
O	5.629987	5.066736	6.368206
Si	6.720868	3.875262	6.677244
C	8.516867	4.412539	6.313792
H	8.647302	4.630241	5.259170
H	8.782429	5.306860	6.870257
H	9.226858	3.636494	6.578846
C	6.309984	2.300937	5.682450
H	6.886323	1.441616	6.005662
H	5.257462	2.045380	5.772822
H	6.518713	2.450517	4.627755
H	5.851001	5.682816	8.921649
H	7.569056	5.386453	9.187992
O	6.454426	7.149566	1.015048
C	5.736291	6.324739	1.887113
H	4.754028	6.125046	1.461115
C	6.456135	5.007189	2.236210
H	7.309592	4.855462	1.561979
C	6.449773	3.635021	8.567653
C	7.497060	2.670224	9.123314
H	7.329248	2.494835	10.185681
H	7.449804	1.714328	8.608875
C	6.572692	4.968711	9.308162
C	5.059660	3.055118	8.841615
H	4.288418	3.707540	8.439271
H	4.953771	2.073218	8.386245
H	6.387637	4.831075	10.373562
H	4.894657	2.951080	9.914227
H	7.785249	10.132518	1.670132
H	8.497693	3.075303	9.000115
O	-4.947505	7.099732	3.652750
C	-5.833430	6.391472	4.508196
H	-6.258949	7.118617	5.203460
C	-6.917892	5.791953	3.604965
H	-7.200765	4.797869	3.956638
O	-8.083667	6.570047	3.497886
C	-8.070605	7.277092	2.263227
C	-7.534425	8.701062	2.431352
H	-8.265415	9.305295	2.959049
H	-6.605850	8.694879	2.994957
H	-7.344605	9.118151	1.446498
C	-9.481654	7.274978	1.683350
H	-9.803517	6.250291	1.524872
H	-9.486814	7.803438	0.736301
C	-5.111240	5.298851	5.305336
H	-4.708437	4.550131	4.614281
H	-4.281162	5.757513	5.854119
O	-5.976225	4.644493	6.170472
Si	-6.691258	5.237730	7.534100
C	-8.092015	6.479676	7.144563
H	-7.736261	7.506216	7.157171
H	-8.520096	6.292685	6.163411
H	-8.895112	6.417404	7.871499
C	-5.392504	6.099821	8.638714
H	-5.813098	6.429421	9.582355
H	-4.561205	5.436500	8.861639
H	-4.984234	6.976865	8.143231
H	-8.012331	2.767590	6.439798
H	-9.273723	3.689843	7.258472
O	-7.223440	6.510776	1.407029
C	-6.298637	5.810455	2.192411
H	-6.149090	4.817176	1.770632
C	-4.949656	6.541952	2.357333
H	-4.871965	7.364019	1.632610
C	-7.383918	3.627102	8.322016
C	-8.026817	3.944127	9.672545

H	-8.437080	3.039474	10.120485
H	-7.295443	4.361279	10.359507
C	-8.443067	3.005077	7.408614
C	-6.268625	2.601795	8.543326
H	-5.779910	2.362399	7.602423
H	-5.520459	2.986429	9.232433
H	-8.831906	2.085770	7.846870
H	-6.675331	1.681759	8.963589
H	-10.154615	7.756580	2.384467
N	-3.794582	5.687329	2.165809
O	4.917290	-7.138556	3.174134
C	5.836101	-6.550193	4.082585
H	6.245224	-7.357744	4.693741
C	6.925334	-5.895626	3.226930
H	7.264415	-4.964614	3.685690
O	8.050451	-6.706777	2.992497
C	7.991588	-7.231813	1.670711
C	7.396621	-8.642497	1.643174
H	8.132745	-9.358764	1.993562
H	6.518554	-8.698063	2.280247
H	7.104984	-8.870924	0.621521
C	9.391148	-7.203147	1.064993
H	9.761508	-6.182621	1.060918
H	9.349397	-7.574628	0.046946
C	5.157829	-5.524985	4.997689
H	4.796166	-4.682380	4.397271
H	4.302222	-6.006716	5.484185
O	6.041965	-5.020165	5.940281
Si	6.753842	-5.827070	7.192230
C	8.155203	-6.991235	6.610661
H	7.808830	-8.014790	6.497226
H	8.557401	-6.674529	5.652350
H	8.975583	-7.015430	7.320254
C	5.453438	-6.858564	8.138427
H	5.872353	-7.340430	9.015037
H	4.622011	-6.241096	8.467236
H	5.045636	-7.640925	7.503675
H	7.973051	-3.166207	6.508866
H	9.286199	-4.178819	7.111022
O	7.166558	-6.318593	0.948298
C	6.270964	-5.717740	1.841194
H	6.125788	-4.675896	1.556711
C	4.915788	-6.452068	1.942280
H	4.829415	-7.197552	1.139403
C	7.436624	-4.361045	8.229585
C	8.134224	-4.886195	9.484442
H	8.524198	-4.058616	10.076389
H	7.441474	-5.449117	10.104244
C	8.445508	-3.556157	7.406470
C	6.302274	-3.428145	8.661862
H	5.775199	-3.040961	7.793599
H	5.589249	-3.953400	9.292870
H	8.828430	-2.717438	7.987816
H	6.698464	-2.586145	9.229028
H	10.052812	-7.821058	1.662414
O	-7.047739	-4.949374	3.446066
C	-6.347193	-5.880953	4.258002
H	-7.082554	-6.339169	4.925948
C	-5.757185	-6.924934	3.302994
H	-4.772819	-7.245813	3.649514
O	-6.560601	-8.066224	3.128293
C	-7.236790	-7.986242	1.879117
C	-8.657745	-7.437916	2.036403
H	-9.290824	-8.191151	2.494396
H	-8.656899	-6.549048	2.660913
H	-9.036868	-7.176136	1.052579
C	-7.240081	-9.367818	1.232659
H	-6.216828	-9.704475	1.098054
H	-7.730951	-9.313943	0.267102
C	-5.248160	-5.200361	5.086614
H	-4.532016	-4.727040	4.405092
H	-5.703205	-4.424858	5.710755
O	-4.553142	-6.130052	5.847480
Si	-4.621656	-6.487616	7.454417
C	-4.761171	-4.885541	8.484014
H	-3.946959	-4.210171	8.236232
H	-5.691223	-4.358815	8.289141
H	-4.712797	-5.076447	9.550380
C	-2.926812	-7.313662	7.692549
H	-2.751045	-7.550031	8.735536
H	-2.848366	-8.234130	7.124281
H	-2.128897	-6.657334	7.360980
H	-7.625509	-6.459714	7.008985
H	-7.425082	-6.223799	8.744029
O	-6.439101	-7.111732	1.081611
C	-5.752055	-6.230922	1.926184
H	-4.752368	-6.060519	1.528571
C	-6.486367	-4.891609	2.152890
H	-7.307562	-4.782275	1.431176
C	-6.068766	-7.694935	7.901716
C	-5.824180	-8.321348	9.279146
H	-6.642460	-8.991121	9.540205
H	-4.901164	-8.894195	9.282176
C	-7.416860	-6.965779	7.949284
C	-6.154114	-8.826436	6.871210
H	-6.318332	-8.439714	5.868317
H	-5.232473	-9.402938	6.861166
H	-8.223272	-7.673952	8.138207
H	-6.973465	-9.501606	7.116876
H	-7.762472	-10.063459	1.880456
N	-5.634643	-3.727631	2.011640
H	8.964933	-5.536598	9.224307
H	-5.753287	-7.555240	10.046911
H	-8.835091	4.661503	9.558940

Electronic energy = -19512.666693

713

E-**G3₈** (H-to-T)

O	1.204868	1.795564	0.476986
N	3.342969	1.056600	0.661653
C	4.686799	1.247988	0.817567
N	5.227216	2.429871	1.041696
C	4.355378	3.442164	1.104398
C	2.978401	3.338925	0.877311
C	2.399964	2.059131	0.647520
N	4.602773	4.757678	1.390653
C	3.382010	5.405457	1.285400
N	2.407858	4.576736	0.989531
H	5.125188	-0.775098	0.719959
H	6.461020	0.349759	0.900264
H	2.969211	0.103620	0.565502
N	0.298723	-5.555083	1.315908
O	1.944318	-1.335370	0.669506
N	1.193021	-3.444871	1.038797
C	1.379111	-4.778842	1.270488
N	2.566668	-5.322930	1.454766
C	3.593933	-4.467311	1.379730
C	3.489821	-3.102681	1.085726
C	2.203694	-2.519039	0.907680
N	4.929444	-4.721600	1.547916
C	5.579095	-3.523268	1.303123
N	4.739664	-2.549315	1.042924
H	-0.651819	-5.191425	1.234132

H	0.452922	-6.535674	1.473496
H	0.238385	-3.071570	0.943444
N	-5.453092	-0.354064	0.910135
O	-1.232375	-2.124377	0.855674
N	-3.343995	-1.306563	0.962563
C	-4.702074	-1.444211	1.046728
N	-5.293769	-2.604715	1.246071
C	-4.464231	-3.650686	1.342188
C	-3.072944	-3.606417	1.196874
C	-2.436881	-2.347641	0.988180
N	-4.788056	-4.959885	1.562933
C	-3.595606	-5.664445	1.486931
N	-2.565699	-4.874535	1.289671
H	-5.064830	0.594791	0.918969
H	-6.444164	-0.478209	1.029077
H	-2.937726	-0.373340	0.827200
N	-0.283342	5.382430	0.597289
O	-1.936249	1.113147	0.622692
N	-1.174406	3.250909	0.700979
C	-1.348695	4.604739	0.776663
N	-2.517489	5.167434	1.008001
C	-3.532104	4.307841	1.159870
C	-3.450329	2.918209	1.011969
C	-2.185384	2.314088	0.757014
N	-4.834184	4.588549	1.465632
C	-5.499571	3.371957	1.456489
N	-4.690094	2.371517	1.195788
H	0.673127	5.011161	0.609174
H	-0.428484	6.369846	0.722437
H	-0.231574	2.870185	0.561429
N	5.483011	0.185709	0.732651
N	5.446109	0.839857	-2.315679
O	1.091494	2.233831	-2.283583
N	3.272631	1.619319	-2.313938
C	4.614721	1.872234	-2.298338
N	5.109852	3.098608	-2.283548
C	4.189614	4.071556	-2.254192
C	2.804438	3.894256	-2.234153
C	2.277374	2.571769	-2.272210
C	3.148813	6.011391	-2.193443
N	2.190939	5.117277	-2.185369
H	5.129953	-0.139189	-2.272856
H	6.430145	1.040068	-2.295925
H	2.934335	0.647624	-2.305642
N	0.507011	-5.113536	-1.862628
O	1.901504	-0.762969	-2.096669
N	1.286486	-2.942788	-2.005244
C	1.536956	-4.283883	-1.964736
N	2.759687	-4.783842	-2.022756
C	3.731592	-3.865077	-2.095436
C	3.561627	-2.479093	-2.072011
C	2.239474	-1.948346	-2.047402
C	5.671725	-2.835815	-2.231016
N	4.788772	-1.873066	-2.141216
H	-0.471250	-4.795363	-1.880219
H	0.704327	-6.097848	-1.909930
H	0.315494	-2.607250	-1.980619
N	-5.483841	-0.223851	-2.221846
O	-1.133773	-1.596629	-1.973301
N	-3.314696	-0.994115	-2.085433
C	-4.654647	-1.254167	-2.136485
N	-5.147521	-2.482128	-2.121079
C	-4.226196	-3.450193	-2.039417
C	-2.842849	-3.264209	-1.965717
C	-2.318309	-1.940478	-1.998284
N	-4.434977	-4.801055	-2.011588
C	-3.176660	-5.383282	-1.933383

N	-2.225288	-4.483427	-1.890949
H	-5.161796	0.751982	-2.183188
H	-6.468274	-0.420488	-2.252906
H	-2.978423	-0.023182	-2.119055
N	-0.533919	5.751276	-2.463418
O	-1.911201	1.399964	-2.177698
N	-1.304777	3.582002	-2.308742
C	-1.562859	4.922645	-2.339228
N	-2.787387	5.417895	-2.278597
C	-3.753757	4.496204	-2.183500
C	-3.572490	3.111975	-2.138783
C	-2.251148	2.586395	-2.201046
N	-5.102474	4.710301	-2.117487
C	-5.689287	3.451752	-2.053710
N	-4.792363	2.496076	-2.047274
H	0.443376	5.438103	-2.395528
H	-0.732552	6.735855	-2.460287
H	-0.332387	3.245925	-2.350009
K	-0.028271	0.251278	-0.899950
C	3.128492	6.823865	1.475492
C	3.973066	7.829259	0.999675
C	1.938562	7.187334	2.119517
C	3.636519	9.155180	1.151646
C	1.595347	8.506789	2.268350
C	2.439262	9.519470	1.786880
H	4.892406	7.565943	0.500466
H	1.302938	6.409431	2.510809
H	4.274582	9.933429	0.762607
H	0.683571	8.788317	2.770782
C	-6.916480	3.159988	1.700320
C	-7.289953	2.001168	2.394301
C	-7.916573	4.008730	1.220746
C	-8.612134	1.694473	2.592669
C	-9.244470	3.708069	1.418897
C	-9.618565	2.543953	2.107434
H	-6.515890	1.355708	2.776266
H	-7.653774	4.893839	0.663846
H	-8.900185	0.807051	3.133561
H	-10.018431	4.346444	1.021676
C	-3.460898	-7.110620	1.552874
C	-2.335432	-7.651172	2.182401
C	-4.388000	-7.974821	0.963231
C	-2.151548	-9.011864	2.236525
C	-4.202001	-9.336411	1.005545
C	-3.079204	-9.879027	1.645517
H	-1.630070	-6.979910	2.643249
H	-5.250782	-7.572773	0.456289
H	-1.294052	-9.439857	2.729852
H	-4.915866	-10.007127	0.553548
C	7.015598	-3.287858	1.309240
C	7.932513	-4.140069	0.690383
C	7.477254	-2.110992	1.909507
C	9.272583	-3.824299	0.667753
C	8.811447	-1.791257	1.886735
C	9.736454	-2.645202	1.269455
H	7.586611	-5.051147	0.226682
H	6.767976	-1.471572	2.409537
H	9.983602	-4.467690	0.173476
H	9.176101	-0.890742	2.354521
C	7.102739	-2.621598	-2.351618
C	7.877879	-3.378076	-3.238199
C	7.721707	-1.629666	-1.585564
C	9.237488	-3.192157	-3.306665
C	9.081538	-1.432605	-1.660774
C	9.868127	-2.223565	-2.512572
H	7.405749	-4.099229	-3.886133
H	7.115661	-1.039842	-0.918424

H	9.837751	-3.774244	-3.988274
H	9.570945	-0.691058	-1.048429
C	2.904708	7.441056	-2.169839
C	3.688397	8.337897	-2.905633
C	1.836786	7.938400	-1.414952
C	3.454307	9.689664	-2.832897
C	1.588720	9.289885	-1.353757
C	2.406763	10.194672	-2.048903
H	4.468221	7.969634	-3.551948
H	1.228576	7.242630	-0.861002
H	4.059422	10.382092	-3.396796
H	0.787380	9.681362	-0.746078
C	-7.117512	3.206470	-2.013695
C	-7.610718	2.143460	-1.249018
C	-8.018091	3.985621	-2.751129
C	-8.962402	1.899067	-1.177642
C	-9.369386	3.756842	-2.665520
C	-9.871347	2.717669	-1.867147
H	-6.914603	1.535647	-0.694774
H	-7.651693	4.752426	-3.414482
H	-9.350560	1.102338	-0.561981
H	-10.063511	4.358505	-3.230577
C	-2.915322	-6.811985	-1.916736
C	-1.860618	-7.298370	-1.134933
C	-3.648131	-7.708126	-2.700507
C	-1.565115	-8.640841	-1.111155
C	-3.356948	-9.053367	-2.671816
C	-2.320193	-9.545603	-1.870921
H	-1.300588	-6.601523	-0.534951
H	-4.421856	-7.346627	-3.358366
H	-0.758230	-9.024563	-0.507368
H	-3.917640	-9.749841	-3.274634
N	2.341891	11.551083	-1.906347
N	1.218972	12.131494	-1.743526
C	2.354734	14.353294	-1.266517
N	1.894739	15.559918	-1.022477
C	1.258016	13.458819	-1.484531
C	0.110885	14.263992	-1.355907
N	0.550474	15.492808	-1.081645
C	-1.318002	13.918918	-1.504264
H	-1.411775	12.853761	-1.690652
H	-1.867869	14.184518	-0.603490
H	-1.752311	14.464449	-2.341887
C	3.799164	14.032780	-1.313487
H	4.385371	14.927109	-1.123092
H	4.030830	13.263326	-0.578363
H	4.049898	13.629414	-2.293357
C	-0.239498	16.656528	-0.803844
H	-0.993421	16.792561	-1.578341
H	-0.727862	16.544815	0.165629
H	0.428341	17.513615	-0.782853
N	-11.222513	2.666063	-1.692042
N	-11.828107	1.555268	-1.556730
C	-14.013514	2.720249	-0.986010
N	-15.229950	2.278446	-0.757260
C	-13.147574	1.614278	-1.263662
C	-13.979341	0.481759	-1.182570
N	-15.196276	0.936944	-0.881710
C	-13.663851	-0.945086	-1.403575
H	-12.599596	-1.048788	-1.590774
H	-13.944885	-1.537633	-0.535181
H	-14.212065	-1.323027	-2.266455
C	-13.654358	4.156054	-0.963479
H	-14.541768	4.759945	-0.799303
H	-12.924674	4.339442	-0.175602
H	-13.187842	4.429564	-1.908297
C	-16.379696	0.164547	-0.637318
H	-16.482505	-0.609899	-1.395550
H	-16.319113	-0.292759	0.352179
H	-17.231366	0.838581	-0.676122
N	-2.114095	-10.906451	-1.936010
N	-1.208787	-11.395471	-1.167732
C	-1.699626	-13.741419	-2.011181
N	-1.251452	-14.927955	-1.665669
C	-1.034701	-12.733975	-1.239045
C	-0.130002	-13.447131	-0.430472
N	-0.306420	-14.737777	-0.722215
C	0.885381	-12.948437	0.520696
H	0.667980	-11.910521	0.754082
H	0.889904	-13.544711	1.429448
H	1.875640	-13.001787	0.066912
C	-2.703681	-13.538210	-3.079682
H	-3.064525	-14.496429	-3.443065
H	-3.522443	-12.928489	-2.705284
H	-2.245414	-12.988167	-3.901077
C	0.342238	-15.869776	-0.124079
H	1.417442	-15.704792	-0.078750
H	-0.050748	-16.030691	0.882927
H	0.125521	-16.737759	-0.741510
N	11.024686	-2.184293	1.216976
N	11.990213	-3.014724	1.109581
C	13.663275	-1.108525	0.996660
N	14.973385	-1.068795	0.903034
C	13.225640	-2.472936	1.007874
C	14.410589	-3.226882	0.919339
N	15.408151	-2.343401	0.856580
C	14.609753	-4.691583	0.909885
H	13.641597	-5.182513	0.929445
H	15.158037	-4.995855	0.020192
H	15.181666	-5.001610	1.784360
C	12.822202	0.103769	1.109044
H	13.448863	0.988538	1.167884
H	12.156616	0.169737	0.249785
H	12.198890	0.028368	1.998408
C	16.806988	-2.616706	0.699838
H	17.114811	-3.400351	1.390455
H	17.006158	-2.930797	-0.326487
H	17.349542	-1.699448	0.912991
N	11.234448	-2.210121	-2.533111
N	11.870254	-1.121653	-2.341824
C	14.090149	-2.354472	-2.288367
N	15.335324	-1.954137	-2.159326
C	13.217902	-1.219131	-2.266764
C	14.076469	-0.113247	-2.126033
N	15.312861	-0.610259	-2.065346
C	13.772595	1.331899	-2.075195
H	14.200270	1.836997	-2.941171
H	12.696470	1.473247	-2.082161
H	14.196953	1.777410	-1.177626
C	13.709987	-3.775795	-2.451391
H	14.597936	-4.401368	-2.455299
H	13.039141	-4.073104	-1.646684
H	13.166937	-3.900472	-3.387004
C	16.532786	0.115907	-1.864616
H	16.597781	0.943494	-2.570049
H	16.566275	0.501332	-0.844156
H	17.358434	-0.572322	-2.024713
N	1.949219	10.792383	1.851491
N	2.739082	11.789499	1.943390
C	0.793368	13.414560	1.781373
N	0.714743	14.725184	1.832903
C	2.162470	13.012528	1.912835
C	2.875374	14.218335	2.051160
N	1.968023	15.193997	1.993551

C	4.321758	14.449941	2.248763
H	4.843647	13.499074	2.201894
H	4.709701	15.120835	1.484660
H	4.501060	14.903209	3.223743
C	-0.390066	12.536923	1.643274
H	-1.299650	13.127991	1.691176
H	-0.341394	12.000824	0.696403
H	-0.384114	11.793903	2.438891
C	2.203792	16.608126	2.029605
H	2.913583	16.848539	2.819244
H	2.597798	16.937340	1.066080
H	1.253163	17.098781	2.221235
N	-10.901425	2.087210	2.209252
N	-11.876100	2.909084	2.262012
C	-13.556367	1.010762	2.111626
N	-14.869688	0.974292	2.121707
C	-13.115326	2.368291	2.235036
C	-14.301415	3.120556	2.326463
N	-15.302950	2.243433	2.250889
C	-14.495868	4.575764	2.494032
H	-13.529004	5.069096	2.476054
H	-15.124789	4.969499	1.697810
H	-14.982251	4.784635	3.446855
C	-12.714171	-0.202034	2.011094
H	-12.040806	-0.247050	2.865505
H	-13.340553	-1.088914	1.986879
H	-12.097507	-0.149402	1.115127
C	-16.708274	2.526674	2.228448
H	-16.965805	3.214242	3.032747
H	-16.975971	2.970083	1.267691
H	-17.238587	1.587087	2.358920
N	-2.822359	-11.220565	1.800560
N	-3.502926	-12.041997	1.088465
C	-2.254421	-14.014275	2.093378
N	-2.243622	-15.298786	1.814501
C	-3.215921	-13.351807	1.261910
C	-3.791546	-14.383056	0.496123
N	-3.172932	-15.507815	0.861009
C	-4.890782	-14.338009	-0.489839
H	-5.115212	-13.301183	-0.720807
H	-4.613531	-14.869530	-1.397161
H	-5.783552	-14.807845	-0.075822
C	-1.406687	-13.416478	3.149615
H	-2.036550	-13.117352	3.987079
H	-0.674536	-14.140702	3.495879
H	-0.926635	-12.517545	2.771673
C	-3.353840	-16.820794	0.312899
H	-4.409941	-17.087558	0.314061
H	-2.970244	-16.847156	-0.709205
H	-2.794809	-17.516697	0.933159
O	4.571081	-6.820288	2.576276
C	4.556958	-6.708950	3.993814
H	4.331382	-7.701398	4.390177
C	5.964290	-6.255529	4.411987
H	5.903322	-5.519696	5.217189
O	6.821570	-7.283135	4.834689
C	7.698212	-7.635391	3.771106
C	7.171143	-8.849726	3.003198
H	7.088330	-9.691453	3.683670
H	6.195720	-8.636167	2.574904
H	7.865815	-9.092029	2.205503
C	9.089229	-7.897075	4.341934
H	9.424975	-7.021762	4.890337
H	9.047769	-8.748490	5.012677
H	9.777991	-8.095055	3.527827
C	3.503571	-5.713978	4.501927
H	3.675288	-4.734836	4.040250

H	2.512443	-6.066360	4.203575
O	3.587624	-5.542268	5.876004
Si	3.216570	-6.598847	7.088306
C	4.391755	-8.102293	7.164573
H	4.031441	-8.923813	6.552010
H	5.386252	-7.843503	6.812229
H	4.488225	-8.483148	8.175755
C	1.428567	-7.237193	6.883477
H	1.082389	-7.767102	7.763975
H	0.739508	-6.418832	6.697379
H	1.357242	-7.924103	6.045399
C	3.430232	-5.453072	8.618852
C	3.115994	-6.223611	9.901564
H	3.788552	-7.068557	10.021518
H	3.228201	-5.576041	10.770673
H	2.096006	-6.598287	9.887668
C	4.870251	-4.939116	8.697739
H	5.116305	-4.372397	7.803711
C	2.487297	-4.249542	8.533284
H	2.644086	-3.582312	9.380812
H	2.665770	-3.690330	7.618501
H	1.449403	-4.572714	8.543309
H	4.995825	-4.287798	9.562717
H	5.571486	-5.765090	8.786958
O	7.758225	-6.479460	2.933344
C	6.596881	-5.721930	3.112968
H	6.879291	-4.670145	3.166571
C	5.524895	-5.973545	2.016461
H	5.960739	-6.495900	1.153600
O	-6.948945	-4.424992	2.354174
C	-6.967519	-4.268789	3.765441
H	-7.979410	-3.957932	4.040027
C	-6.630779	-5.644773	4.360995
H	-5.987005	-5.535308	5.236715
O	-7.737186	-6.419721	4.738933
C	-7.992417	-7.403147	3.743219
C	-9.091097	-6.941053	2.783745
H	-10.002224	-6.764916	3.347375
H	-8.797563	-6.023791	2.280315
H	-9.260753	-7.716681	2.043698
C	-8.362450	-8.716550	4.426861
H	-7.567878	-9.004792	5.108817
H	-9.283625	-8.583871	4.983710
H	-8.486605	-9.488997	3.675514
C	-5.977884	-3.207767	4.270109
H	-4.948553	-3.548526	4.109207
H	-6.126832	-2.285817	3.699653
O	-6.229897	-2.985417	5.618389
Si	-5.189290	-2.799789	6.877417
C	-4.377087	-1.072779	6.832997
H	-3.776817	-0.950092	5.935326
H	-5.124988	-0.285906	6.835112
H	-3.724348	-0.909354	7.683588
C	-3.793210	-4.102651	6.832164
H	-3.130930	-4.010479	7.685767
H	-4.180379	-5.117573	6.826832
H	-3.185389	-3.977258	5.939885
C	-6.366000	-3.036408	8.378154
C	-5.605480	-2.793077	9.682749
H	-5.209761	-1.781365	9.715272
H	-6.266411	-2.927358	10.538231
H	-4.775649	-3.487903	9.782949
C	-7.533473	-2.049464	8.301340
H	-8.083994	-2.189107	7.375441
C	-6.933338	-4.459239	8.399678
H	-7.636703	-4.572805	9.224025
H	-7.454876	-4.678748	7.471079

H	-6.139172	-5.190221	8.532052
H	-8.215279	-2.202579	9.137467
H	-7.175054	-1.023693	8.338962
O	-6.749902	-7.579940	3.059227
C	-5.985634	-6.418347	3.193701
H	-4.950900	-6.709602	3.379630
C	-6.099728	-5.459609	1.974688
H	-6.573172	-5.958875	1.119364
O	-4.384448	6.831103	2.075298
C	-4.061308	6.992270	3.450562
H	-3.853712	8.054098	3.609813
C	-5.298127	6.552489	4.250192
H	-4.997641	5.995478	5.141315
O	-6.142745	7.594197	4.661340
C	-7.279677	7.652874	3.807246
C	-7.095979	8.711906	2.718285
H	-6.969173	9.682075	3.188950
H	-6.223598	8.491267	2.109096
H	-7.976914	8.721726	2.084090
C	-8.520198	7.930412	4.651572
H	-8.621849	7.159113	5.409263
H	-8.414732	8.896218	5.133727
H	-9.397085	7.923842	4.013445
C	-2.828182	6.183195	3.872276
H	-3.042003	5.109870	3.800836
H	-2.003530	6.409758	3.190363
O	-2.502745	6.534571	5.177697
Si	-1.490068	5.744032	6.206651
C	0.275883	5.669426	5.484230
H	0.296366	5.038097	4.599505
H	0.628577	6.654464	5.193498
H	0.989041	5.257689	6.189699
C	-2.092761	3.956083	6.501484
H	-1.490325	3.437235	7.238842
H	-3.123346	3.934944	6.843520
H	-2.041048	3.378524	5.582170
C	-1.623250	6.835645	7.781814
C	-0.769307	6.238978	8.901800
H	0.273953	6.179530	8.602780
H	-0.831952	6.856592	9.796979
H	-1.108818	5.238258	9.155855
C	-1.131106	8.256511	7.494922
H	-1.713245	8.701331	6.692813
C	-3.075943	6.913705	8.260423
H	-3.151337	7.558105	9.135716
H	-3.711522	7.320136	7.477871
H	-3.447253	5.928707	8.533232
H	-1.235100	8.879029	8.383180
H	-0.083448	8.251545	7.204440
O	-7.394157	6.344831	3.242607
C	-6.137349	5.737992	3.245628
H	-6.263890	4.689979	3.523147
C	-5.377208	5.875844	1.895109
H	-6.045195	6.251974	1.108644
O	6.759024	4.314137	2.250379
C	6.715247	4.071802	3.650288
H	7.728716	3.799692	3.957529
C	6.276249	5.389566	4.305660
H	5.608356	5.193056	5.148483
O	7.326164	6.199965	4.764616
C	7.549662	7.261000	3.842755
C	8.709559	6.935571	2.899338
H	9.617901	6.812450	3.480993
H	8.509930	6.021281	2.347075
H	8.829754	7.754255	2.197108
C	7.808279	8.547783	4.621463
H	6.964532	8.749997	5.274627
H	8.705406	8.429548	5.219421
H	7.927559	9.370376	3.924699
C	5.751997	2.937069	4.028933
H	4.732626	3.213900	3.732856
H	6.036667	2.036760	3.479358
O	5.746627	2.725937	5.399572
Si	6.524667	1.591622	6.311327
C	6.856120	0.024815	5.272551
H	5.921491	-0.368027	4.881662
H	7.506131	0.234999	4.428531
H	7.321982	-0.762001	5.854868
C	5.235495	1.250001	7.668390
H	5.568599	0.468999	8.341941
H	5.039491	2.137875	8.260523
H	4.291831	0.933831	7.234758
C	8.174581	2.270502	7.062964
C	8.534898	1.475215	8.322652
H	8.642688	0.417498	8.096426
H	9.475374	1.833284	8.739100
H	7.764632	1.585382	9.080518
C	9.344681	2.150581	6.079874
H	9.132086	2.677377	5.152259
C	8.014158	3.743326	7.453905
H	8.928566	4.113257	7.916774
H	7.798798	4.360285	6.584905
H	7.201258	3.865128	8.165722
H	10.245182	2.583617	6.514010
H	9.546396	1.108043	5.847777
O	6.323477	7.401802	3.122985
C	5.631972	6.189104	3.156650
H	4.573612	6.402555	3.310165
C	5.860448	5.313734	1.890411
H	6.331177	5.899842	1.089189
O	-6.068728	6.417462	-3.429058
C	-5.128268	7.396712	-3.865511
H	-5.702465	8.163677	-4.396662
C	-4.485656	7.986026	-2.592798
H	-3.399393	8.009230	-2.670004
O	-4.939076	9.274235	-2.279382
C	-6.016837	9.176134	-1.359223
C	-7.374355	9.165458	-2.069964
H	-7.469325	10.065065	-2.670078
H	-7.463575	8.293830	-2.712488
H	-8.166448	9.136383	-1.328163
C	-5.911677	10.344771	-0.384083
H	-4.976171	10.272485	0.161562
H	-6.741209	10.320643	0.312873
C	-4.105467	6.824125	-4.850748
H	-3.277827	7.533318	-4.943976
H	-3.721534	5.875009	-4.466920
O	-4.634303	6.654039	-6.126594
Si	-5.788783	5.607241	-6.649840
C	-7.552439	6.245703	-6.289963
H	-7.711672	6.351417	-5.222222
H	-7.719288	7.218035	-6.743815
H	-8.309770	5.568972	-6.671214
C	-5.559984	3.880873	-5.871843
H	-6.301305	3.168726	-6.215454
H	-4.578518	3.471521	-6.094999
H	-5.647233	3.939349	-4.791634
H	-4.935428	7.700419	-8.614080
H	-6.618891	7.354272	-9.014900
O	-5.807341	7.942473	-0.662153
C	-4.988312	7.102639	-1.435015
H	-4.194485	6.704209	-0.800015
C	-5.788465	5.981330	-2.123183
H	-6.748749	5.827113	-1.613862

C	-5.438360	5.598133	-8.542844
C	-6.419988	4.664058	-9.250227
H	-6.233798	4.656096	-10.324034
H	-6.316966	3.647333	-8.880390
C	-5.595616	7.004790	-9.124761
C	-4.013927	5.114816	-8.827463
H	-3.289099	5.754624	-8.330576
H	-3.872117	4.095317	-8.475657
H	-5.348016	7.007734	-10.186331
H	-3.812095	5.133831	-9.898655
H	-5.927520	11.269597	-0.951045
H	-7.444864	4.985752	-9.085732
O	6.050344	6.454234	-3.559165
C	7.022724	5.548658	-4.067584
H	7.746958	6.140935	-4.633851
C	7.699472	4.890818	-2.852368
H	7.779370	3.813138	-2.994807
O	8.975756	5.394011	-2.562209
C	8.866736	6.399003	-1.565737
C	8.766604	7.797769	-2.182628
H	9.655183	7.988427	-2.776419
H	7.888435	7.873496	-2.818141
H	8.692105	8.535379	-1.389528
C	10.072193	6.285365	-0.637555
H	10.080978	5.301465	-0.178777
H	10.012628	7.043519	0.134771
C	6.402530	4.519509	-5.018317
H	5.786323	3.816676	-4.448919
H	5.767619	5.053993	-5.734006
O	7.379170	3.785704	-5.678788
Si	8.408776	4.309809	-6.855299
C	9.838937	5.363554	-6.145117
H	9.611950	6.425832	-6.175926
H	10.036666	5.097546	-5.110154
H	10.757762	5.222538	-6.704706
C	7.468871	5.351447	-8.152271
H	8.120639	5.702439	-8.944793
H	6.666902	4.781361	-8.613320
H	7.020673	6.227495	-7.690773
H	9.421243	1.766145	-5.642287
H	10.834795	2.555738	-6.341093
O	7.674515	6.082608	-0.837826
C	6.833374	5.297244	-1.643119
H	6.469429	4.453084	-1.055842
C	5.673833	6.113793	-2.246679
H	5.535011	7.052207	-1.693619
C	9.059024	2.653036	-7.580580
C	9.869160	2.924030	-8.848843
H	10.272108	1.993671	-9.248202
H	9.247568	3.380228	-9.614557
C	9.962193	1.948451	-6.565997
C	7.899357	1.718437	-7.933478
H	7.299185	1.507883	-7.052255
H	7.256920	2.166020	-8.688463
H	10.303779	0.990663	-6.958927
H	8.277642	0.774468	-8.326213
H	10.976962	6.418714	-1.220933
N	4.406805	5.420637	-2.223078
O	-6.035601	-5.820457	-3.408486
C	-7.001037	-4.908500	-3.921661
H	-7.728551	-5.496887	-4.488335
C	-7.681726	-4.243531	-2.710431
H	-7.704107	-3.160617	-2.826872
O	-8.987945	-4.690752	-2.471053
C	-8.950088	-5.759895	-1.539550
C	-8.912860	-7.123662	-2.237364
H	-9.783899	-7.219022	-2.878278
H	-8.011575	-7.223169	-2.836010
H	-8.923552	-7.909459	-1.488326
C	-10.166631	-5.637120	-0.626455
H	-10.104249	-4.707599	-0.069120
H	-10.195103	-6.471095	0.065094
C	-6.373570	-3.895111	-4.883498
H	-5.773649	-3.172306	-4.321747
H	-5.720910	-4.442906	-5.573440
O	-7.340770	-3.187809	-5.585909
Si	-8.431677	-3.788425	-6.668571
C	-9.931189	-4.594660	-5.795979
H	-9.860107	-5.678746	-5.790997
H	-10.000155	-4.265384	-4.762983
H	-10.865551	-4.343408	-6.286569
C	-7.605242	-5.089992	-7.798490
H	-8.290665	-5.483046	-8.541540
H	-6.752565	-4.671656	-8.326370
H	-7.242758	-5.931922	-7.214470
H	-8.952774	-0.915364	-5.912785
H	-10.521552	-1.642114	-6.258941
O	-7.754084	-5.557962	-0.777158
C	-6.875077	-4.721352	-1.487157
H	-6.538133	-3.919624	-0.827154
C	-5.693069	-5.507921	-2.081031
H	-5.553570	-6.456471	-1.546605
C	-8.950383	-2.214497	-7.640840
C	-9.929436	-2.590892	-8.753304
H	-10.241245	-1.702163	-9.301381
H	-9.468630	-3.278752	-9.457103
C	-9.630941	-1.210419	-6.708040
C	-7.731064	-1.541261	-8.275746
H	-7.002172	-1.277737	-7.513721
H	-7.253939	-2.202506	-8.995397
H	-9.924185	-0.316831	-7.259430
H	-8.028168	-0.631949	-8.798113
H	-11.061631	-5.631870	-1.239523
O	5.899650	-5.735560	-3.674587
C	4.947900	-6.728362	-4.038519
H	5.469744	-7.458850	-4.666480
C	4.476779	-7.398165	-2.736098
H	3.391343	-7.498467	-2.728618
O	5.039424	-8.663500	-2.509315
C	6.168158	-8.529369	-1.660437
C	7.470074	-8.413386	-2.459630
H	7.603131	-9.311468	-3.054939
H	7.440995	-7.549092	-3.117625
H	8.303829	-8.304298	-1.772830
C	6.203169	-9.724086	-0.712684
H	5.306742	-9.722025	-0.100333
H	7.077761	-9.658501	-0.075538
C	3.785576	-6.129388	-4.841001
H	3.235229	-5.429828	-4.203521
H	4.197139	-5.581750	-5.694718
O	2.902016	-7.121634	-5.246409
Si	2.662616	-7.808399	-6.724218
C	2.718881	-6.478026	-8.093239
H	2.008111	-5.686114	-7.873654
H	3.700930	-6.020206	-8.172927
H	2.467727	-6.880383	-9.068346
C	0.906914	-8.501949	-6.512096
H	0.562485	-8.973897	-7.424847
H	0.865883	-9.239925	-5.718727
H	0.204981	-7.713695	-6.261405
H	5.692499	-7.993699	-6.730673
H	5.235237	-8.081111	-8.429903
O	5.931991	-7.335788	-0.904857
C	5.035592	-6.512605	-1.605580

H	4.274900	-6.147308	-0.914651
C	5.754140	-5.356436	-2.325621
H	6.762962	-5.215896	-1.915914
C	3.932113	-9.218329	-7.118248
C	3.430274	-10.072200	-8.288233
H	4.147069	-10.860070	-8.516044
H	2.479222	-10.536869	-8.043999
C	5.302690	-8.650166	-7.505802
C	4.099046	-10.131059	-5.898117
H	4.430304	-9.572204	-5.026091
H	3.154417	-10.607767	-5.648425
H	6.018354	-9.457945	-7.657080
H	4.829498	-10.912649	-6.106090
H	6.237085	-10.635425	-1.300121
N	5.074994	-4.089310	-2.200938
H	-10.817758	-3.065697	-8.345495
H	3.295316	-9.466550	-9.180590
H	10.700751	3.592566	-8.642248

Electronic energy = -19512.6269054

713

Z-G3$_8$ (H-to-H)

O	-0.206364	-2.312354	1.258487
N	1.917060	-3.104631	1.314290
C	2.844444	-4.101745	1.383013
N	2.519879	-5.377283	1.507432
C	1.202147	-5.621113	1.525823
C	0.193595	-4.658906	1.435001
C	0.552393	-3.284052	1.326354
N	0.575676	-6.832145	1.627479
C	-0.786821	-6.561022	1.601705
N	-1.028589	-5.277292	1.475591
H	4.458271	-2.798577	1.304537
H	4.797216	-4.507506	1.469609
H	2.224495	-2.128194	1.223061
N	3.790503	3.938154	1.611046
O	2.343946	-0.372069	1.107271
N	3.132093	1.738200	1.371366
C	4.125995	2.653144	1.558103
N	5.399347	2.318910	1.681975
C	5.645133	1.005472	1.575518
C	4.685726	0.012475	1.361313
C	3.312643	0.378685	1.262167
N	6.853170	0.368436	1.644161
C	6.583818	-0.984180	1.476980
N	5.302812	-1.208332	1.297096
H	2.819705	4.268188	1.567089
H	4.520083	4.594162	1.828165
H	2.155518	2.052782	1.311522
N	-3.924097	3.633516	1.596292
O	0.398848	2.177641	1.268313
N	-1.717080	2.968696	1.464461
C	-2.637053	3.964865	1.603370
N	-2.305471	5.238054	1.736013
C	-0.988463	5.482269	1.678821
C	0.010227	4.520191	1.509567
C	-0.354379	3.147034	1.399432
N	-0.353524	6.691842	1.751935
C	1.004040	6.419560	1.629993
N	1.232634	5.135976	1.475796
H	-4.253117	2.661852	1.550537
H	-4.586763	4.365793	1.781512
H	-2.029837	1.992670	1.385576
N	-3.596198	-4.082408	1.609374
O	-2.139541	0.241262	1.311464
N	-2.933717	-1.876207	1.477367
C	-3.931179	-2.797171	1.604662
N	-5.205875	-2.465237	1.730626
C	-5.447350	-1.146788	1.677881
C	-4.485607	-0.149065	1.502101
C	-3.110939	-0.513565	1.414411
N	-6.654656	-0.509389	1.759814
C	-6.384026	0.847037	1.626204
N	-5.101090	1.073679	1.462902
H	-2.626205	-4.412597	1.555522
H	-4.329144	-4.752628	1.759134
H	-1.958088	-2.190362	1.401357
N	4.129992	-3.771797	1.314104
N	-4.485698	3.098362	-1.735345
O	-2.055901	-0.757799	-1.326734
N	-3.324517	1.117244	-1.528690
C	-4.510579	1.769598	-1.697543
N	-5.671428	1.145550	-1.821457
C	-5.595631	-0.190562	-1.734373
C	-4.423344	-0.922844	-1.532015
C	-3.173520	-0.247143	-1.444770
C	-6.011937	-2.350861	-1.688305
N	-4.719295	-2.258040	-1.497434
H	-3.616910	3.639612	-1.669395
H	-5.346990	3.571831	-1.944969
H	-2.448401	1.652456	-1.467397
N	3.133355	4.491782	-1.722936
O	-0.759560	2.114349	-1.372590
N	1.129286	3.354500	-1.562684
C	1.805742	4.531903	-1.687650
N	1.199918	5.704799	-1.767313
C	-0.136955	5.651885	-1.690571
C	-0.898566	4.488830	-1.553386
C	-0.238102	3.228219	-1.483679
C	-2.286144	6.125540	-1.612921
N	-2.226812	4.818267	-1.502950
H	3.667609	3.615467	-1.702523
H	3.611933	5.353931	-1.918114
H	1.648858	2.468008	-1.521393
N	4.515996	-3.112133	-1.904032
O	2.144873	0.779193	-1.511068
N	3.384225	-1.108431	-1.724012
C	4.560711	-1.784457	-1.861134
N	5.733737	-1.179166	-1.951444
C	5.682262	0.157297	-1.854454
C	4.519928	0.917192	-1.696995
C	3.257893	0.258613	-1.630430
N	6.723932	1.043648	-1.863891
C	6.157073	2.305495	-1.722724
N	4.850383	2.243415	-1.612467
H	3.641775	-3.645309	-1.858265
H	5.377056	-3.598528	-2.082414
H	2.502610	-1.630230	-1.648872
N	-3.099500	-4.522834	-1.515956
O	0.814498	-2.145158	-1.455083
N	-1.086884	-3.381156	-1.516206
C	-1.774214	-4.557411	-1.601594
N	-1.177791	-5.726702	-1.750301
C	0.159989	-5.675341	-1.781553
C	0.934888	-4.518752	-1.669042
C	0.284955	-3.257462	-1.535563
N	1.032157	-6.718644	-1.918469
C	2.302997	-6.158133	-1.889791
N	2.261851	-4.855494	-1.730442
H	-3.629926	-3.641798	-1.498582
H	-3.592434	-5.379757	-1.702267
H	-1.597299	-2.495473	-1.421062
K	0.057328	-0.041425	-0.097628

C	-1.831872	-7.565590	1.657585
C	-2.984273	-7.375384	0.880345
C	-1.732881	-8.718798	2.438776
C	-3.965544	-8.332733	0.833683
C	-2.734410	-9.663714	2.424429
C	-3.862776	-9.502967	1.604412
H	-3.060300	-6.481741	0.283222
H	-0.884765	-8.862698	3.087971
H	-4.829533	-8.208626	0.200722
H	-2.635468	-10.563538	3.014472
C	-7.382267	1.898658	1.571470
C	-8.562024	1.892073	2.318894
C	-7.155744	2.968154	0.691052
C	-9.505186	2.881585	2.152013
C	-8.107735	3.935927	0.496483
C	-9.316342	3.908065	1.212389
H	-8.729399	1.133140	3.064799
H	-6.233768	2.988447	0.135552
H	-10.426220	2.847857	2.716179
H	-7.946430	4.733726	-0.210889
C	2.060456	7.412300	1.597813
C	2.029263	8.598238	2.334591
C	3.162545	7.170151	0.762917
C	3.040788	9.524814	2.215586
C	4.153277	8.106567	0.615435
C	4.114103	9.311613	1.336136
H	1.231246	8.783534	3.034192
H	3.191434	6.249229	0.205442
H	2.990979	10.450850	2.769859
H	4.978787	7.935173	-0.056600
C	7.579385	-2.037650	1.444270
C	7.374138	-3.117010	0.571277
C	8.735102	-2.022590	2.227755
C	8.321006	-4.099582	0.431947
C	9.673362	-3.023326	2.112658
C	9.501592	-4.070491	1.193151
H	6.474077	-3.134317	-0.020880
H	8.886428	-1.244769	2.957786
H	8.179207	-4.910192	-0.265062
H	10.578020	-2.984068	2.701948
C	-3.537107	6.856305	-1.563078
C	-3.763746	8.048565	-2.254157
C	-4.569357	6.334643	-0.768353
C	-4.964567	8.711360	-2.133351
C	-5.756033	7.006456	-0.624235
C	-5.983001	8.212689	-1.306510
H	-3.017184	8.439551	-2.925039
H	-4.394564	5.410798	-0.243673
H	-5.118429	9.646561	-2.652053
H	-6.537004	6.618020	0.009767
C	-6.665334	-3.638623	-1.854974
C	-7.481316	-3.896409	-2.957600
C	-6.332102	-4.693227	-0.995885
C	-7.925071	-5.175099	-3.216449
C	-6.779001	-5.966063	-1.246586
C	-7.566596	-6.244661	-2.378502
H	-7.743138	-3.093843	-3.628884
H	-5.708988	-4.484535	-0.141276
H	-8.502243	-5.372662	-4.108257
H	-6.524200	-6.782034	-0.589612
C	3.543417	-6.901984	-1.986287
C	4.648191	-6.456883	-1.245467
C	3.685124	-8.037800	-2.786137
C	5.832718	-7.148809	-1.265785
C	4.880211	-8.719855	-2.828054
C	5.977156	-8.297077	-2.061496
H	4.534126	-5.578824	-0.631590

H	2.872505	-8.366376	-3.412806
H	6.676081	-6.821588	-0.678747
H	4.970935	-9.611100	-3.431992
C	6.898659	3.548126	-1.622447
C	6.399608	4.544932	-0.769105
C	8.093156	3.793052	-2.304512
C	7.110891	5.693769	-0.536209
C	8.794095	4.960180	-2.097250
C	8.336902	5.923277	-1.183309
H	5.465804	4.366527	-0.264340
H	8.459590	3.085075	-3.029160
H	6.744819	6.438456	0.152254
H	9.734009	5.120514	-2.605311
N	-7.688519	-7.555809	-2.749498
N	-8.558711	-8.145828	-3.398464
C	-10.578186	-8.366907	-4.715571
N	-11.806491	-7.903257	-4.719521
C	-9.833540	-7.762932	-3.653303
C	-10.760188	-6.916876	-3.009483
N	-11.912606	-7.057939	-3.680661
C	-10.642956	-6.107311	-1.775338
H	-9.892991	-6.544619	-1.120387
H	-10.324592	-5.089147	-2.007936
H	-11.595074	-6.067735	-1.252881
C	-10.060155	-9.322741	-5.721267
H	-10.882230	-9.881215	-6.158809
H	-9.542535	-8.786437	-6.515964
H	-9.348459	-9.993610	-5.247251
C	-13.181533	-6.447332	-3.398023
H	-13.571640	-6.798168	-2.441167
H	-13.086106	-5.362012	-3.377478
H	-13.862342	-6.741869	-4.192951
N	7.080115	-9.088065	-1.874757
N	7.717699	-9.773103	-2.683161
C	8.152539	-10.789994	-4.836680
N	8.095860	-10.468731	-6.108530
C	7.697106	-9.694055	-4.037923
C	7.395294	-8.677581	-4.968944
N	7.667530	-9.197356	-6.174585
C	6.982247	-7.266734	-4.785994
H	5.893772	-7.185304	-4.785306
H	7.346412	-6.895420	-3.831666
H	7.378385	-6.642885	-5.582908
C	8.563073	-12.126479	-4.347014
H	9.244445	-12.586854	-5.056530
H	9.034141	-12.022981	-3.373024
H	7.690773	-12.770716	-4.239331
C	7.564421	-8.556401	-7.455075
H	6.555041	-8.175234	-7.611275
H	8.277970	-7.733779	-7.529934
H	7.798359	-9.304369	-8.208624
N	9.147843	6.914937	-0.704158
N	9.970014	7.643814	-1.271609
C	11.284485	8.464756	-3.130397
N	11.129191	8.720254	-4.408760
C	10.074762	7.920243	-2.594070
C	9.177050	7.909771	-3.683205
N	9.860450	8.409691	-4.722304
C	7.738729	7.563485	-3.759802
H	7.614187	6.498484	-3.963781
H	7.254831	7.780868	-2.810619
H	7.247998	8.131541	-4.545625
C	12.562289	8.655569	-2.405627
H	13.113741	9.484364	-2.840187
H	12.355091	8.840942	-1.355149
H	13.175631	7.758393	-2.483893
C	9.389745	8.655369	-6.057003

H	8.961116	7.748529	-6.483200
H	8.642186	9.450476	-6.059954
H	10.248598	8.969682	-6.645093
N	10.534536	-4.890755	0.829133
N	11.412841	-5.436017	1.507540
C	12.635797	-5.919756	3.543690
N	12.381877	-6.163611	4.808770
C	11.415396	-5.653754	2.846304
C	10.409337	-5.798321	3.824708
N	11.047944	-6.114567	4.959955
C	8.932416	-5.761063	3.721358
H	8.625491	-6.053733	2.720725
H	8.478050	-6.434265	4.443296
H	8.563586	-4.750203	3.903559
C	14.004059	-5.865926	2.978338
H	14.679067	-6.457215	3.589851
H	13.986509	-6.237290	1.956915
H	14.362992	-4.837161	2.960359
C	10.472187	-6.411397	6.241685
H	9.816611	-5.601640	6.561288
H	9.903937	-7.342050	6.200740
H	11.294277	-6.522513	6.944419
N	-7.071192	8.965915	-0.952461
N	-7.791321	9.704625	-1.634712
C	-8.456986	10.896176	-3.631030
N	-8.512025	10.697808	-4.927771
C	-7.899459	9.746892	-2.986466
C	-7.661919	8.832549	-4.035059
N	-8.058834	9.453807	-5.154779
C	-7.197044	7.425997	-4.030783
H	-7.661446	6.864677	-4.837489
H	-6.113259	7.383135	-4.153181
H	-7.445878	6.955195	-3.083459
C	-8.857439	12.164494	-2.978811
H	-9.612738	12.664587	-3.577792
H	-9.235201	11.952493	-1.982042
H	-7.997270	12.826976	-2.885266
C	-8.054769	8.940664	-6.495370
H	-7.049479	8.631515	-6.782832
H	-8.733635	8.090388	-6.581423
H	-8.396263	9.740723	-7.147427
N	-4.713483	-10.539762	1.332599
N	-5.205832	-11.399424	2.071336
C	-5.543193	-12.581153	4.158440
N	-5.689026	-12.304822	5.433808
C	-5.313050	-11.375717	3.423705
C	-5.369129	-10.352811	4.393742
N	-5.609214	-10.969761	5.559744
C	-5.319524	-8.878083	4.262155
H	-5.733385	-8.580693	3.301763
H	-5.883994	-8.399261	5.057963
H	-4.287064	-8.526512	4.303959
C	-5.552186	-13.958213	3.612572
H	-4.536068	-14.348833	3.563079
H	-6.139077	-14.606646	4.256510
H	-5.960987	-13.943452	2.605552
C	-5.798484	-10.374501	6.852968
H	-4.940842	-9.755501	7.116101
H	-6.703843	-9.765770	6.867571
H	-5.899909	-11.187532	7.567691
N	-10.345187	4.701873	0.787671
N	-11.253087	5.263001	1.411690
C	-12.560152	5.832139	3.368126
N	-12.363050	6.126982	4.632205
C	-11.310104	5.535355	2.738525
C	-10.348456	5.717928	3.755326
N	-11.037510	6.080927	4.846054

C	-8.868375	5.672260	3.721428
H	-8.513580	4.663291	3.939882
H	-8.513947	5.944147	2.730316
H	-8.443486	6.356866	4.450951
C	-13.900931	5.759387	2.742305
H	-14.280452	4.738768	2.784475
H	-14.592128	6.406088	3.274628
H	-13.826699	6.053143	1.698484
C	-10.522844	6.425342	6.141631
H	-9.908740	5.614930	6.534216
H	-9.927918	7.338415	6.088725
H	-11.377925	6.589830	6.792688
N	4.960925	10.320509	0.964537
N	5.505547	11.207810	1.631879
C	5.967561	12.487847	3.634156
N	6.168864	12.275704	4.914143
C	5.686454	11.249445	2.975103
C	5.774728	10.277318	3.994341
N	6.077209	10.950283	5.113318
C	5.695533	8.799006	3.947970
H	6.014581	8.442402	2.971952
H	6.326778	8.353851	4.712738
H	4.667438	8.468293	4.106779
C	5.968099	13.834335	3.016413
H	4.954546	14.233417	2.985236
H	6.586214	14.508565	3.601821
H	6.337380	13.762216	1.996728
C	6.317102	10.418426	6.425391
H	5.463699	9.827190	6.757650
H	7.213337	9.796174	6.429786
H	6.462376	11.264624	7.092404
O	8.542578	0.893145	3.200384
C	8.360703	2.156232	3.828942
H	9.203740	2.299997	4.510127
C	8.380906	3.216464	2.714600
H	7.578877	3.941038	2.857192
O	9.590108	3.918103	2.601055
C	10.388067	3.309461	1.597944
C	11.348259	2.279509	2.204055
H	11.944597	2.767449	2.969225
H	10.798523	1.455231	2.649184
H	12.003129	1.891088	1.430141
C	11.151470	4.394750	0.846601
H	10.460425	5.166294	0.519631
H	11.890885	4.837278	1.504978
H	11.639682	3.955623	-0.017018
C	7.065836	2.195630	4.647081
H	6.205516	2.215662	3.970145
H	7.021824	1.284946	5.255611
O	6.995964	3.324232	5.453147
Si	7.979953	3.702120	6.724205
C	9.636793	4.454900	6.138527
H	9.944446	5.289111	6.760161
H	10.442578	3.727072	6.171338
H	9.563404	4.813486	5.115369
C	8.333579	2.152122	7.782830
H	8.921356	2.384583	8.664142
H	7.412818	1.681211	8.116245
H	8.887902	1.413200	7.210106
C	6.918134	4.994218	7.674187
C	7.646973	5.432664	8.944882
H	8.613144	5.869946	8.707925
H	7.060022	6.176141	9.483307
H	7.810989	4.586893	9.606891
C	6.676203	6.225778	6.798883
H	6.154130	5.938589	5.890856
C	5.561479	4.404910	8.070382

H	4.954028	5.154931	8.576880
H	5.023257	4.063164	7.190018
H	5.688768	3.562305	8.745626
H	6.071428	6.956585	7.335555
H	7.620814	6.689118	6.525767
O	9.456327	2.689597	0.705147
C	8.269154	2.414118	1.403172
H	7.423002	2.707431	0.783210
C	8.166522	0.942321	1.843390
H	8.873393	0.325752	1.272069
O	-0.897603	8.419070	3.259813
C	-2.165171	8.247399	3.881877
H	-2.311252	9.097782	4.553195
C	-3.215404	8.256288	2.758944
H	-3.957147	7.473386	2.920998
O	-3.892494	9.475701	2.601785
C	-3.285035	10.216790	1.554142
C	-2.237998	11.193544	2.098713
H	-2.721671	11.890513	2.776197
H	-1.455137	10.660619	2.630445
H	-1.792377	11.738447	1.272248
C	-4.370630	10.950208	0.773487
H	-5.121151	10.239487	0.440461
H	-4.839314	11.688456	1.414922
H	-3.925992	11.435490	-0.088739
C	-2.215477	6.958425	4.709813
H	-2.208129	6.092340	4.039995
H	-1.322613	6.926274	5.344604
O	-3.367466	6.884981	5.481017
Si	-3.768884	7.837085	6.769010
C	-4.431867	9.541958	6.214245
H	-5.239886	9.888532	6.849833
H	-3.657840	10.303607	6.249809
H	-4.804891	9.503310	5.194303
C	-2.258028	8.096196	7.907942
H	-2.507194	8.671410	8.792949
H	-1.841547	7.148306	8.237456
H	-1.471095	8.633019	7.384680
C	-5.140852	6.784492	7.611371
C	-5.623273	7.477881	8.885943
H	-6.022306	8.464153	8.664747
H	-6.408534	6.893078	9.363956
H	-4.808039	7.593225	9.594899
C	-6.331367	6.616316	6.664783
H	-6.011257	6.123748	5.751430
C	-4.614841	5.394889	7.982163
H	-5.408143	4.793697	8.426295
H	-4.245775	4.879935	7.098908
H	-3.803855	5.467833	8.702668
H	-7.105460	6.011942	7.136974
H	-6.753502	7.584566	6.408613
O	-2.682544	9.237479	0.703641
C	-2.404902	8.087689	1.458864
H	-2.691339	7.212280	0.878367
C	-0.934760	8.007770	1.912641
H	-0.318779	8.706369	1.330022
O	-8.278203	-0.957712	3.395203
C	-8.032692	-2.181738	4.074784
H	-8.824172	-2.299262	4.819982
C	-8.118962	-3.296022	3.018793
H	-7.314206	-4.018732	3.153763
O	-9.339272	-3.989497	3.018314
C	-10.195046	-3.417566	2.040117
C	-11.104821	-2.341471	2.641828
H	-11.685232	-2.781509	3.447180
H	-10.520024	-1.514234	3.032496
H	-11.775904	-1.967289	1.874638

C	-11.018540	-4.533713	1.406943
H	-10.348480	-5.290034	1.011934
H	-11.656969	-4.977554	2.163217
H	-11.621872	-4.124315	0.604256
C	-6.685297	-2.169026	4.804678
H	-5.868724	-2.204599	4.076503
H	-6.615583	-1.234477	5.373166
O	-6.545383	-3.264854	5.645667
Si	-7.400752	-3.582219	7.021860
C	-9.117792	-4.327602	6.628617
H	-9.904797	-3.582423	6.703843
H	-9.146746	-4.734124	5.621270
H	-9.377882	-5.126324	7.315173
C	-7.634558	-1.990192	8.049785
H	-8.137496	-2.181536	8.991427
H	-6.681129	-1.520208	8.275015
H	-8.233721	-1.265396	7.504842
C	-6.262747	-4.857922	7.901606
C	-6.862544	-5.247948	9.252892
H	-7.851835	-5.680474	9.129744
H	-6.230584	-5.981683	9.752184
H	-6.950055	-4.380291	9.901021
C	-6.121320	-6.118178	7.045276
H	-5.691745	-5.864328	6.080535
C	-4.867639	-4.273368	8.139402
H	-4.219388	-5.014170	8.607450
H	-4.417000	-3.967503	7.198576
H	-4.917977	-3.407438	8.794964
H	-5.470411	-6.838739	7.540132
H	-7.092159	-6.579788	6.884170
O	-9.315697	-2.868206	1.054465
C	-8.086332	-2.561560	1.661183
H	-7.281828	-2.881536	0.999961
C	-7.959932	-1.071344	2.025882
H	-8.689261	-0.480473	1.456917
O	1.283924	-8.579176	3.044295
C	2.619345	-8.441786	3.513627
H	2.825850	-9.305845	4.153841
C	3.528768	-8.466124	2.275228
H	4.323411	-7.725503	2.369354
O	4.120205	-9.712227	2.010817
C	3.386648	-10.375751	0.991825
C	2.352545	-11.336047	1.588176
H	2.867690	-12.128883	2.121673
H	1.693920	-10.812697	2.275353
H	1.755462	-11.759974	0.786758
C	4.361716	-11.109033	0.076936
H	5.076120	-10.400488	-0.331905
H	4.894246	-11.860954	0.648971
H	3.812439	-11.576623	-0.733137
C	2.796353	-7.156228	4.333557
H	2.749582	-6.292492	3.660974
H	1.974689	-7.087875	5.052924
O	4.035208	-7.153103	4.964607
Si	4.402767	-7.225387	6.568661
C	3.474879	-5.854195	7.520954
H	3.612309	-4.896520	7.026886
H	2.405926	-6.044015	7.564295
H	3.828464	-5.750152	8.540659
C	6.267761	-6.873575	6.488846
H	6.718112	-6.927995	7.472842
H	6.766242	-7.592129	5.847826
H	6.453302	-5.882287	6.090692
C	4.040409	-8.950160	7.369094
C	4.525454	-8.984410	8.822570
H	4.006891	-8.240878	9.421865
H	4.338773	-9.964469	9.259659

H	5.592141	-8.785529	8.877933
C	2.538313	-9.257611	7.363966
H	2.139262	-9.259546	6.352040
C	4.770751	-10.052874	6.594199
H	4.508998	-11.033944	6.990060
H	4.512300	-10.029087	5.537753
H	5.847224	-9.929808	6.679448
H	2.352205	-10.238530	7.801154
H	1.989984	-8.521039	7.947398
O	2.753074	-9.332460	0.247985
C	2.579891	-8.221690	1.085772
H	2.812924	-7.320835	0.520881
C	1.170481	-8.149021	1.707029
H	0.493060	-8.839392	1.185881
O	0.693777	-8.509334	-3.416385
C	-0.645978	-8.630871	-3.882122
H	-0.678187	-9.490655	-4.556979
C	-1.525148	-8.880254	-2.646687
H	-2.443138	-8.293992	-2.706812
O	-1.874768	-10.224585	-2.442359
C	-1.002314	-10.794951	-1.479575
C	0.183873	-11.497280	-2.148628
H	-0.185961	-12.314701	-2.759854
H	0.737826	-10.804427	-2.775146
H	0.848452	-11.885215	-1.382778
C	-1.793891	-11.761199	-0.604924
H	-2.656523	-11.249886	-0.188211
H	-1.160388	-12.118093	0.200131
C	-1.086462	-7.379629	-4.650139
H	-1.078325	-6.520826	-3.971923
H	-0.370866	-7.202808	-5.461342
O	-2.375025	-7.502967	-5.149406
Si	-2.910288	-8.514952	-6.337463
C	-3.105483	-10.315709	-5.732430
H	-2.247083	-10.926166	-5.998334
H	-3.212482	-10.355255	-4.652086
H	-3.977436	-10.792802	-6.166885
C	-1.696309	-8.491598	-7.815213
H	-2.063508	-9.072386	-8.654273
H	-1.518873	-7.479296	-8.167928
H	-0.734704	-8.910342	-7.530162
H	-5.112122	-7.212037	-4.733962
H	-5.711310	-8.786279	-5.242737
O	-0.563069	-9.694776	-0.677432
C	-0.629608	-8.522325	-1.444972
H	-1.032885	-7.723669	-0.825629
C	0.727192	-8.126998	-2.059924
H	1.538284	-8.682006	-1.569339
C	-4.603476	-7.718302	-6.776433
C	-5.252484	-8.484956	-7.928855
H	-6.215472	-8.043833	-8.184178
H	-4.622818	-8.458295	-8.814252
C	-5.538705	-7.764217	-5.566673
C	-4.429664	-6.257968	-7.203009
H	-3.953077	-5.684633	-6.411963
H	-3.817747	-6.186240	-8.099198
H	-6.504021	-7.320646	-5.811139
H	-5.398868	-5.807327	-7.417748
H	-2.134500	-12.595720	-1.207855
H	-5.416633	-9.524338	-7.658955
O	-8.457598	-0.986914	-3.280731
C	-8.885711	0.278448	-3.768512
H	-9.771670	0.100853	-4.382351
C	-9.228939	1.135941	-2.545613
H	-8.912203	2.169101	-2.700698
O	-10.591786	1.144906	-2.197684
C	-10.794769	0.343590	-1.041924

C	-11.275358	-1.063084	-1.418374
H	-12.330002	-1.030704	-1.671971
H	-10.718645	-1.436155	-2.273941
H	-11.110814	-1.726557	-0.573370
C	-11.781584	1.045314	-0.115448
H	-11.389585	2.023473	0.147283
H	-11.913089	0.453343	0.783985
C	-7.813967	0.955399	-4.629757
H	-6.959259	1.223676	-3.998380
H	-7.484375	0.247053	-5.397307
O	-8.303422	2.119593	-5.211120
Si	-9.443119	2.236229	-6.400729
C	-11.203579	1.791275	-5.804244
H	-11.428194	0.738738	-5.954621
H	-11.325583	2.007753	-4.746120
H	-11.960466	2.351238	-6.343762
C	-8.983177	1.097032	-7.863178
H	-9.676191	1.196808	-8.691251
H	-7.986544	1.312542	-8.238337
H	-8.995981	0.054139	-7.557161
H	-9.249278	4.785396	-4.801186
H	-10.884994	4.694078	-5.446601
O	-9.522195	0.291889	-0.394518
C	-8.527007	0.435819	-1.367347
H	-7.702194	1.009138	-0.946357
C	-8.035647	-0.912651	-1.939861
H	-8.498615	-1.747034	-1.391812
C	-9.342686	4.102597	-6.852377
C	-10.212574	4.382198	-8.078654
H	-10.180984	5.440356	-8.337044
H	-9.864221	3.814471	-8.937061
C	-9.856943	4.950559	-5.686463
C	-7.905056	4.519766	-7.172646
H	-7.254348	4.321488	-6.325642
H	-7.528458	3.967840	-8.030155
H	-9.828481	6.009930	-5.942706
H	-7.860825	5.582339	-7.411452
H	-12.730208	1.168066	-0.626723
N	-6.608573	-1.107962	-1.820436
O	8.580091	0.958004	-3.304437
C	8.890301	-0.286271	-3.917395
H	9.780496	-0.127066	-4.530086
C	9.177229	-1.280656	-2.785675
H	8.762416	-2.261574	-3.023413
O	10.539940	-1.447083	-2.482363
C	10.853774	-0.714887	-1.306350
C	11.415696	0.671056	-1.644704
H	12.444139	0.575657	-1.977970
H	10.832132	1.139044	-2.432307
H	11.366404	1.296431	-0.757885
C	11.833374	-1.523699	-0.463638
H	11.394024	-2.488305	-0.226703
H	12.046566	-0.986363	0.454153
C	7.755434	-0.782926	-4.819825
H	6.892326	-1.057957	-4.203399
H	7.468054	0.029515	-5.495796
O	8.146438	-1.907800	-5.537068
Si	9.218744	-1.965770	-6.790987
C	11.026378	-1.650239	-6.257721
H	11.280569	-0.594091	-6.287726
H	11.202645	-2.005037	-5.245560
H	11.728335	-2.158540	-6.910807
C	8.738819	-0.682633	-8.122310
H	9.397610	-0.722369	-8.982773
H	7.723197	-0.836740	-8.476089
H	8.793779	0.326065	-7.721385
H	9.099016	-4.638766	-5.412953

H	10.661331	-4.547442	-6.220305
O	9.617668	-0.607635	-0.596880
C	8.570572	-0.628543	-1.527972
H	7.734640	-1.183353	-1.105934
C	8.142050	0.784796	-1.975015
H	8.646255	1.535676	-1.349338
C	9.010672	-3.778187	-7.397864
C	9.751942	-3.963103	-8.722669
H	9.671998	-4.994312	-9.065687
H	9.335695	-3.317961	-9.491414
C	9.603458	-4.744760	-6.369459
C	7.535372	-4.125402	-7.612847
H	6.979238	-4.005623	-6.687284
H	7.093466	-3.474219	-8.362954
H	9.501821	-5.775586	-6.709663
H	7.431722	-5.154065	-7.958223
H	12.747024	-1.678874	-1.027027
O	-0.873103	8.550850	-3.163559
C	0.402785	8.715692	-3.769502
H	0.340481	9.601465	-4.409361
C	1.407239	8.948846	-2.630869
H	2.334426	8.405873	-2.819599
O	1.725804	10.298659	-2.409165
C	0.968268	10.787917	-1.313055
C	-0.312724	11.482556	-1.786681
H	-0.048558	12.357003	-2.373348
H	-0.911700	10.812138	-2.396147
H	-0.895358	11.783066	-0.921336
C	1.839339	11.735669	-0.495717
H	2.752156	11.225831	-0.202221
H	1.296906	12.049433	0.389641
C	0.783136	7.500562	-4.625637
H	0.917541	6.632195	-3.971127
H	-0.033646	7.295556	-5.325155
O	1.981839	7.718863	-5.291636
Si	2.280611	8.144675	-6.855250
C	1.141472	7.170542	-8.038223
H	1.230429	6.103942	-7.851451
H	0.096986	7.438433	-7.905630
H	1.390621	7.340808	-9.079759
C	4.085215	7.569047	-7.012931
H	4.463835	7.737997	-8.014028
H	4.724317	8.100556	-6.317025
H	4.173137	6.509579	-6.797419
H	0.089252	10.205815	-6.406687
H	0.173050	10.001973	-8.155446
O	0.665613	9.634775	-0.523391
C	0.666145	8.504933	-1.355278
H	1.154680	7.685566	-0.831096
C	-0.746639	8.110241	-1.830222
H	-1.504284	8.631008	-1.228218
C	2.117386	10.044776	-7.190421
C	2.809787	10.408635	-8.508911
H	2.707632	11.474805	-8.707280
H	3.868915	10.171288	-8.466043
C	0.649703	10.474364	-7.299801
C	2.786519	10.842028	-6.065147
H	2.359734	10.601233	-5.094380
H	3.850382	10.623083	-6.025152
H	0.580992	11.554407	-7.428018
H	2.667892	11.911867	-6.235663
H	2.095334	12.598196	-1.101302
N	-1.022533	6.693927	-1.721681
H	10.806629	-3.725321	-8.613335
H	2.371049	9.864793	-9.341482
H	-11.248148	4.112626	-7.889934

Electronic energy = -19512.515129

References

1. Moreno, J.; Gerecke, M.; Grubert, L.; Kovalenko, S. A.; Hecht, S., Sensitized Two-NIR-Photon Z→E Isomerization of a Visible-Light-Addressable Bistable Azobenzene Derivative. *Angew. Chem. Int. Ed.* **2016,** *55* (4), 1544-1547.
2. González-Rodríguez, D.; Janssen, P. G. A.; Martín-Rapún, R.; Cat, I. D.; Feyter, S. D.; Schenning, A. P. H. J.; Meijer, E. W., Persistent, Well-Defined, Monodisperse, π-Conjugated Organic Nanoparticles via G-Quadruplex Self-Assembly. *J. Am. Chem. Soc.* **2010,** *132* (13), 4710-4719.
3. Mutruc, D.; Goulet-Hanssens, A.; Fairman, S.; Wahl, S.; Zimathies, A.; Knie, C.; Hecht, S., Modulating Guest Uptake in Core–Shell MOFs with Visible Light. *Angew. Chem. Int. Ed.* **2019,** *58* (37), 12862-12867.
4. Gubala, V.; Betancourt, J. E.; Rivera, J. M., Expanding the Hoogsteen Edge of 2'-Deoxyguanosine: Consequences for G-Quadruplex Formation. *Org. Lett.* **2004,** *6* (25), 4735-4738.
5. Gibalova, A.; Kortekaas, L.; Simke, J.; Ravoo, B. J., Multi-responsive Electropolymer Surface Coatings Based on Azo Molecular Switches and Carbazoles: Light, pH, and Electrochemical Control of Z→E Isomerization in Thin Films. *Chem. Eur. J.* **2023,** *29* (63), e202302215.
6. Das, G.; Prakasam, T.; Addicoat, M. A.; Sharma, S. K.; Ravaux, F.; Mathew, R.; Baias, M.; Jagannathan, R.; Olson, M. A.; Trabolsi, A., Azobenzene-Equipped Covalent Organic Framework: Light-Operated Reservoir. *J. Am. Chem. Soc.* **2019,** *141* (48), 19078-19087.
7. M. J. Frisch, G. W. T., H. B. Schlegel, G. E. Scuseria, M. A. Robb, J. R. Cheeseman, G. Scalmani, V. Barone, G. A. Petersson, H. Nakatsuji, X. Li, M. Caricato, A. V. Marenich, J. Bloino, B. G. Janesko, R. Gomperts, B. Mennucci, H. P. Hratchian, J. V. Ortiz, A. F. Izmaylov, J. L. Sonnenberg, Williams, F. Ding, F. Lipparini, F. Egidi, J. Goings, B. Peng, A. Petrone, T. Henderson, D. Ranasinghe, V. G. Zakrzewski, J. Gao, N. Rega, G. Zheng, W. Liang, M. Hada, M. Ehara, K. Toyota, R. Fukuda, J. Hasegawa, M. Ishida, T. Nakajima, Y. Honda, O. Kitao, H. Nakai, T. Vreven, K. Throssell, J. A. Montgomery Jr., J. E. Peralta, F. Ogliaro, M. J. Bearpark, J. J. Heyd, E. N. Brothers, K. N. Kudin, V. N. Staroverov, T. A. Keith, R. Kobayashi, J. Normand, K. Raghavachari, A. P. Rendell, J. C. Burant, S. S. Iyengar, J. Tomasi, M. Cossi, J. M. Millam, M. Klene, C. Adamo, R. Cammi, J. W. Ochterski, R. L. Martin, K. Morokuma, O. Farkas, J. B. Foresman, D. J. Fox, 2016.
8. Adamo, C.; Barone, V., Toward reliable density functional methods without adjustable parameters: The PBE0 model. *J. Phys. Chem.* **1999,** *110* (13), 6158-6170.
9. Grimme, S.; Antony, J.; Ehrlich, S.; Krieg, H., A consistent and accurate ab initio parametrization of density functional dispersion correction (DFT-D) for the 94 elements H-Pu. *J. Phys. Chem.* **2010,** *132* (15).
10. Grimme, S.; Ehrlich, S.; Goerigk, L., Effect of the damping function in dispersion corrected density functional theory. *J. Comput. Chem.* **2011,** *32* (7), 1456-1465.
11. Weigend, F.; Ahlrichs, R., Balanced basis sets of split valence, triple zeta valence and quadruple zeta valence quality for H to Rn: Design and assessment of accuracy. *Phys. Chem. Chem. Phys.* **2005,** *7* (18), 3297-3305.
12. Pracht, P.; Bohle, F.; Grimme, S., Automated exploration of the low-energy chemical space with fast quantum chemical methods. *Phys. Chem. Chem. Phys.* **2020,** *22* (14), 7169-7192.

13. Grimme, S., Exploration of Chemical Compound, Conformer, and Reaction Space with Meta-Dynamics Simulations Based on Tight-Binding Quantum Chemical Calculations. *J. Chem. Theory Comput.* **2019,** *15* (5), 2847-2862.
14. Pracht, P.; Grimme, S.; Bannwarth, C.; Bohle, F.; Ehlert, S.; Feldmann, G.; Gorges, J.; Müller, M.; Neudecker, T.; Plett, C.; Spicher, S.; Steinbach, P.; Wesołowski, P. A.; Zeller, F., CREST—A program for the exploration of low-energy molecular chemical space. *J. Phys. Chem.* **2024,** *160* (11), 114110.
15. Bannwarth, C.; Ehlert, S.; Grimme, S., GFN2-xTB—An Accurate and Broadly Parametrized Self-Consistent Tight-Binding Quantum Chemical Method with Multipole Electrostatics and Density-Dependent Dispersion Contributions. *J. Chem. Theory Comput.* **2019,** *15* (3), 1652-1671.
16. Ehlert, S.; Stahn, M.; Spicher, S.; Grimme, S., Robust and Efficient Implicit Solvation Model for Fast Semiempirical Methods. *J. Chem. Theory Comput.* **2021,** *17* (7), 4250-4261.
17. Miertuš, S.; Scrocco, E.; Tomasi, J., Electrostatic interaction of a solute with a continuum. A direct utilizaion of AB initio molecular potentials for the prevision of solvent effects. *Chem. Phys.* **1981,** *55* (1), 117-129.
18. Pascual-Ahuir, J. L.; Silla, E.; Tuñón, I., GEPOL: An improved description of molecular surfaces. III. A new algorithm for the computation of a solvent-excluding surface. *J. Comput. Chem.* **1994,** *15*.
19. Barone, V.; Cossi, M., Quantum Calculation of Molecular Energies and Energy Gradients in Solution by a Conductor Solvent Model. *J. Phys. Chem. A* **1998,** *102* (11), 1995-2001.
20. Runge, E.; Gross, E. K. U., Density-Functional Theory for Time-Dependent Systems. *Phys. Rev. Lett.* **1984,** *52* (12), 997-1000.
21. Chai, J.-D.; Head-Gordon, M., Long-range corrected hybrid density functionals with damped atom–atom dispersion corrections. *Phys. Chem. Chem. Phys.* **2008,** *10* (44), 6615-6620.
22. CYLview, b. L., C. Y., Université de Sherbrooke, 2009 (http://www.cylview.org).
